# The Gastrointestinal Safety of Orforglipron, a GLP‐1 Receptor Agonist, in Adults With or Without Type 2 Diabetes: A Network Meta‐Analysis of Randomized Controlled Trials

**DOI:** 10.1002/edm2.70222

**Published:** 2026-05-11

**Authors:** Ahmed W. Hageen, Ahmed Farid Gadelmawla, Ahmad Omar Saleh, Abdallfatah Abdallfatah, Mohamed Reyad Mohamed, Amira Fahmy El‐Nemr, Odai Maihoub, Ahmed Elsekhary, Safir Eladawi, Biruk Demisse Ayalew, Ahmed Mansour, Ayoup Ahmed Radi, Mohamed Abuelazm, Mohamed Galal Flefel

**Affiliations:** ^1^ Faculty of Medicine Tanta University Tanta Egypt; ^2^ Faculty of Medicine Menoufia University Menoufia Egypt; ^3^ Medical Research Group of Egypt (MRGE) Negida Academy Arlington Massachusetts USA; ^4^ Faculty of Medicine The University of Jordan Amman Jordan; ^5^ Faculty of Medicine October 6 University Giza Egypt; ^6^ Department of Medicine University of Arizona College of Medicine – Phoenix Phoenix Arizona USA; ^7^ Faculty of Medicine Al‐Azhar University Cairo Egypt; ^8^ Department of Pathology National Hospital Latakia Syria; ^9^ Kasr Alainy School of Medicine Cairo University Cairo Egypt; ^10^ Faculty of Medicine Ain Shams University Cairo Egypt; ^11^ Department of Internal Medicine, St. Paul's Hospital Millennium Medical College Addis Ababa University School of Medicine Addis Ababa Ethiopia; ^12^ Faculty of Medicine Minia University Minia Egypt; ^13^ Department of Internal Medicine, Faculty of Medicine Tanta University Tanta Egypt

**Keywords:** glucagon‐like peptide‐1 receptor agonists, liver, obesity, orforglipron, pancreas

## Abstract

**Background and Aim:**

Orforglipron (OFG), an oral small‐molecule glucagon‐like peptide‐1 receptor agonist (GLP‐1 RA), has demonstrated significant weight loss and glycemic benefits in adults with or without type 2 diabetes (T2DM). However, its gastrointestinal (GI), hepatic and pancreatic safety profile has not been systematically evaluated. This network meta‐analysis aimed to assess GI adverse events (AEs), hepatic and pancreatic outcomes, and enzyme changes across different OFG doses.

**Methods:**

A frequentist network meta‐analysis was conducted in accordance with the PRISMA guidelines. PubMed, Embase, Scopus and Web of Science (WOS) were searched for randomized controlled trials (RCTs) that assess the GI effects of OFG in adults with or without T2DM. We considered random‐effects models to express treatment effects as odds ratios (OR) and mean differences (MD) with 95% confidence intervals (95% CI). RStudio software (version 4.5.1) was used for analysis.

**Results:**

All OFG doses (3, 12, 24, 36 and 45 mg) increased GI AEs compared to placebo, with a clear dose–response trend. High‐dose OFG (45 mg) markedly increased nausea (OR 11.48, 95% CI: 6.52–20.21), vomiting (OR 11.48, 95% CI: 6.52–20.21), diarrhoea (OR 3.99, 95% CI: 2.07–7.70) and discontinuation due to GI AEs (OR 10.22, 95% CI: 4.99–20.94). No dose increased pancreatitis risk (*p* > 0.05). Higher doses significantly reduced ALT, with the most significant reduction observed at 24 mg (MD –11.19 IU/L, 95% CI: −19.18 to −3.21). Doses ≥ 12 mg increased lipase (e.g., 24 mg: +28.52 IU/L, 95% CI: 12.02–45.01) and pancreatic amylase (45 mg: +18.20 IU/L, 95% CI: 9.49–26.91), without corresponding increases in clinical events. AST and ALP levels remained similar to those of the placebo. Subgroup analyses showed consistent effects in patients with or without T2DM.

**Conclusion:**

Oral OFG showed dose‐dependent GI adverse effects over 26 weeks. Higher doses improve ALT and elevate pancreatic enzymes without clinical manifestations. The safety profile aligns with established GLP‐1 RAs.

## Graphical abstract

In a network meta‐analysis of five RCTs including 3594 adults with or without type 2 diabetes (T2DM), orforglipron demonstrated dose‐dependent gastrointestinal adverse events (GI AE) and modest ALT reduction at higher doses, without increasing pancreatitis or hepatic event risk over 26 weeks, supporting a safety profile consistent with the Glucagon‐Like Peptide‐1 receptor agonist (GLP‐1 RAs) class.

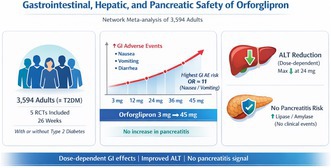

## 1 Introduction

Obesity and type 2 diabetes mellitus (T2DM) are highly prevalent and frequently overlapping conditions that drive a wide spectrum of cardiometabolic and hepatopancreatobiliary complications [1]. Global obesity and T2DM rates continue to rise, and many affected adults have coexisting metabolic dysfunction–associated steatotic liver disease (MASLD) and are at increased risk of pancreatitis [2]. In this setting, pharmacological strategies for weight loss and glycemic control must be evaluated not only for their metabolic efficacy but also for their hepatic and pancreatic safety profiles [3].

Glucagon‐like peptide‐1 receptor agonists (GLP‐1 RAs) have revolutionized the management of obesity and type 2 diabetes mellitus (T2DM) [4, 5]. Across multiple trials, injectable and oral GLP‐1 RAs have produced substantial reductions in body weight and glycated haemoglobin, along with cardiovascular benefits in high‐risk populations [6, 7]. Emerging evidence also suggests that GLP‐1 RAs improve liver enzymes, reduce hepatic fat content, and may lower the risk of progression to advanced MASLD and cirrhosis [5, 8]. At the same time, concerns have been raised regarding possible associations between GLP‐1 RAs and pancreatitis or gallbladder disease [9]. While recent meta‐analyses of randomized trials have not shown a clear increase in pancreatitis risk at the class level, the pancreas and biliary tree remain key organs of interest in ongoing safety surveillance [10, 11, 12].

Orforglipron (OFG) is a once‐daily, orally administered, small‐molecule, non‐peptide GLP‐1 RA developed for the treatment of obesity and T2DM [13, 14]. Phase 2 and phase 3 trials have shown dose‐dependent reductions in body weight and HbA1c in adults with obesity, with or without T2DM, and an overall adverse‐event profile dominated by gastrointestinal symptoms typical of the GLP‐1 RA class [15, 16, 17, 18]. However, available reports provide only limited detail on hepatic and pancreatic laboratory changes, and organ‐specific outcomes have not been systematically characterized in adults stratified by diabetes status.

As oral GLP‐1 RAs move toward broad use in populations with a high background prevalence of MASLD and pancreatitis risk factors, a focused assessment of hepatic and pancreatic safety is needed for individual agents, rather than relying solely on class data [6, 8]. Accordingly, the present study evaluates the pancreatic and hepatic safety, as well as gastrointestinal (GI) adverse events. Both overall GI adverse events (e.g., nausea, vomiting, diarrhoea, constipation) and those specifically leading to drug discontinuation were captured and analysed in adults with or without T2DM. We describe the incidence, severity and temporal patterns of liver and pancreatic enzyme abnormalities, as well as clinically relevant hepatic and pancreatic events, to better define the risk–benefit profile of OFG in this high‐risk metabolic population.

## 2 Methodology

### 2.1 Data Sources and Search Strategy

This network meta‐analysis (NMA) was conducted following the Cochrane Handbook for Systematic Reviews of Interventions (Chapter 11: Undertaking network meta‐analyses) [19] and adhered to the PRISMA guidelines [20]. The protocol for this review was prospectively registered at PROSPERO with (ID: CRD420251249505). The ethical approval and institutional review board were waived because the investigators relied on the data from published articles. We searched on PubMed, Embase, Scopus and Web of Science (WOS) through 21 September 2025 without any automated filters or language restrictions with the following search terms (‘orforglipron’ OR ‘LY3502970’) AND (‘type 2 diabetes’ OR ‘T2DM’ OR ‘diabetes mellitus’ OR ‘diabetes’ OR ‘obesity’ OR ‘obese’ OR ‘overweight’) to retrieve the available RCTs that assess the hepatic and pancreatic safety of OFG (3, 12, 24, 36 and 45 mg) in adults with or without type 2 diabetes mellitus (T2DM). The detailed search strategy with records of each database is shown in (Table S1) and the PRISMA checklist is summarized in (Table S2). A further manual search was conducted using the search strategy on Google Scholar, and the first 200 hits were screened. Additionally, it was applied to the references and citations of included studies to identify any studies that were missed during the primary search.

### 2.2 Selection Criteria and Data Extraction

After retrieving the records from databases, two independent authors (A.A. and A.W.H.) screened the records using Covidance software [21] in two phases: title and abstract screening, followed by full‐text screening. RCTs that evaluate the hepatic and pancreatic safety of OFG in individuals with or without T2DM were included in the analysis. We excluded the non‐randomized studies, non‐human or in vitro studies, conference abstracts, book chapters, reviews, meta‐analyses, editorials, guidelines and duplicate publications or studies with overlapping patient cohorts. The other four independent authors (B.D.A., A.F.E., O.M., A.E. and M.R.M) extracted the study features, baseline characteristics (e.g., number of participants, age in years, sex, weight, race/ethnicity, OFG rout of administration, eligibility criteria and outcomes), hepatic enzymes [e.g., alanine aminotransaminase (ALT), aspartate aminotransferase (AST) and serum alkaline phosphatase (ALP)], pancreatic enzymes (pancreatic amylase and total serum lipase) and study outcomes (%change from baseline in hepatic and pancreatic enzymes at Week 26 IU/L). In addition, the same authors extracted safety outcomes, including cardiac events, mortality, any serious adverse events and adverse events leading to drug discontinuation. Any discrepancies were resolved through consensus with a third reviewer (A.W.H.) through a discussion.

### 2.3 Assessment of the Quality of Included Studies

The Cochrane risk of bias assessment tool (ROB 2.0) [22] was used by two independent authors (M.R. and A.W.H.) to assess the quality of included RCTs. We assessed risk of bias across five domains: randomization process, deviations from intended interventions, missing outcome data, outcome measurement and selective reporting. The RCTs were assessed by two decisions: low risk of bias, some concerns and high risk of bias.

### 2.4 The Geometry of the Network

We presented a network graph with six nodes representing the doses of OFG. The edges represent available direct comparisons between pairs of routes. The line thickness indicates the number of studies involved in these comparisons. The plotted number represents the number of comparisons between each node.

### 2.5 Data Synthesis and Analysis

The frequentist method was employed to conduct NMA of the included studies [23]. Comparing different doses of OFG. Random effects models were used in our research to express treatment effects as odds ratios (OR) and mean differences (MD) with corresponding 95% confidence intervals (95% CI). We calculated the surface under the cumulative ranking curve (SUCRA) for each outcome. The relative ranking of the different doses to determine efficacy and safety was estimated based on the distribution of the SUCRAs. Higher SUCRA values indicated better performance (i.e., fewer gastrointestinal adverse events (GI AEs) and greater reductions in serum enzyme levels from baseline). We utilized the *I* ^2^ statistic to assess heterogeneity in the network analysis. Subgroup analyses were conducted to explore potential differences in treatment effects according to diabetes status (patients with and without DM). Comparisons were made among various OFG dose groups (3, 12, 24, 36 and 45 mg) and placebo, using random‐effects models. Inconsistency in the model was evaluated by comparing estimates from direct and indirect comparisons. In the forest plots, OFG doses were ordered according to effect size rather than nominal dose to facilitate visual interpretation. For adverse event outcomes, doses were arranged from the lowest to the highest odds ratio. For continuous outcomes assessing change from baseline in pancreatic enzymes, doses were ordered according to the magnitude of reduction compared with placebo, with the greatest reduction presented first and the smallest reduction presented last.

We compared the distributions of key study characteristics across studies grouped by contrast to evaluate transitivity. Statistical inconsistencies between direct and indirect evidence were examined using a combination of global and local approaches. As an international approach, we applied a design‐by‐treatment interaction model to investigate inconsistencies from all sources in the entire network [24, 25]. Local inconsistency was evaluated based on the node‐splitting method [26]. Statistical analyses were conducted using the ‘netmeta’ package (version 4.5.1) within RStudio software [27, 28]. A *p*‐value of less than 0.05 was considered statistically significant. Moreover, we relied on the web plot digitizer software [29] to obtain the missing raw data from the figures for the percentage change in hepatic and pancreatic enzymes from baseline in the previously mentioned follow‐ups. Additionally, we have utilized the Meta‐Accelerator web tool to convert the values to mean and standard deviation, ensuring consistent convergence of values [30].

## 3 Result

### 3.1 Search Results and Study Selection

**FIGURE 1:**
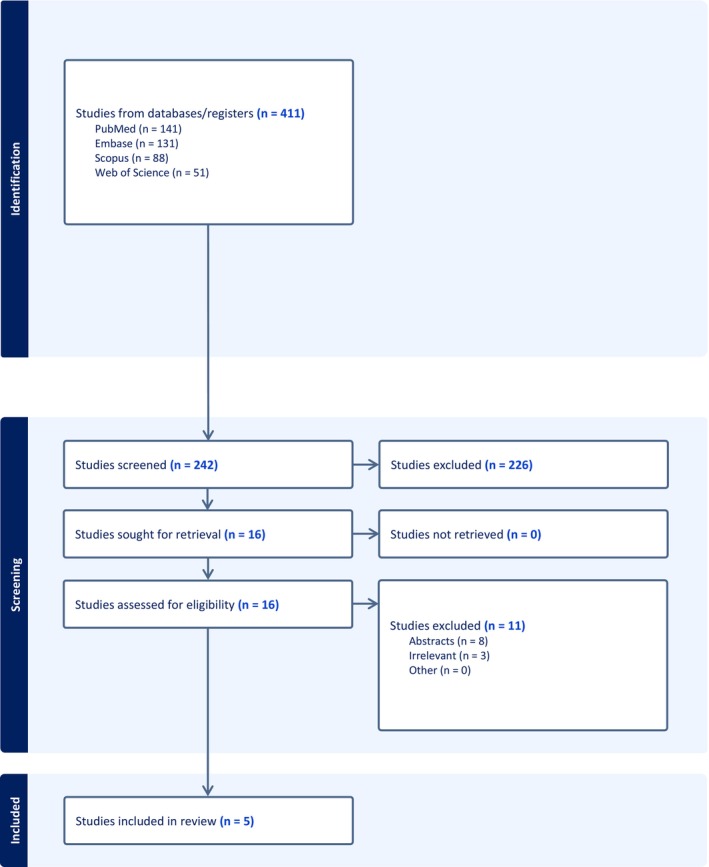
PRISMA flow chart of the screening process.

An electronic search across four databases resulted in 411 references. One hundred sixty‐nine references were removed after Covidence identified duplications; no duplicate references were removed manually. From a total of 242 references, 226 references were excluded after screening the title and abstract. A total of 16 references were retained for full‐text screening; however, 11 studies were excluded after full‐text screening, and five studies met our eligibility criteria (Figure 1).

### 3.2 Characteristics of the Included Studies

Five RCTs included 3594 patients in our data synthesis [30, 31, 32, 33, 34]. A summary of characteristics of included trials is presented in (Table 1), and baseline characteristics of participants are presented in Table 2. The OFG group consisted of 2385 patients, while the control group consisted of 1209 patients. The mean age of patients in the OFG group was 54.9 years, with a range of 44.9–62.8 years. However, the mean age for each OFG dose (3, 12, 24, 36 and 45 mg) was 56.2, 51.7, 58.8, 53.3 and 58.4 years, respectively. Besides, the placebo group had a mean age of 53.3 years, ranging from 45.1 to 58.3 years.

**TABLE 1:** Summary and characteristics of included studies.

Study ID	Design, centres	Trial registry ID	Region (s)	Intervention details	Control details	Inclusion criteria	Outcomes	Sample size* (OFG/Placebo)	Follow‐up, weeks	Main conclusion
Rosenstock et al. 2025 [13]	RCT, multicenter	NCT05971940	China, India, Japan, Mexico and USA	**Oral** OFG at 1 mg, escalating every 4 weeks (to 3, 6, 12, 24 and 36 mg) until they reached their specific assigned maintenance dose (3, 12, or 36 mg once daily), with dose pauses permitted for GI AEs (but no de‐escalation).	Placebo once daily following the same dose escalation schedule as the active group, and like the OFG group, they had a 2‐week safety follow‐up period with no de‐escalation permitted, though temporary OFG pauses were advised for GI AEs	T2DM participants age ≥ 18 years were required to be on diet/exercise only (no insulin or other glucose‐lowering drugs in 3 months), with HbA1c 7.0%–9.5%, BMI ≥ 23 kg/m^2^, and stable weight (≤ 5% change) for 3 months.	GI adverse events.	(421/138)	40 weeks of OFG treatment followed by a 2‐week safety follow‐up (42 weeks)	OFG reduced HbA1c (glycated) over 40 weeks in adults with T2DM
Wharton et al. 2023 (GZGI) [15]	RCT, multicenter	NCT05051579	Canada, USA and Hungary.	**Oral** OFG doses (12, 24, 36, or 45 mg), with dose escalation up to 16 weeks (starting at 2 or 3 mg for 36/45 mg cohorts). Once daily without meal restrictions, and all participants received lifestyle counselling	Placebo once daily for 36 weeks, following the same dose escalation schedule as the OFG groups, with no meal restrictions, and all participants were provided lifestyle education	Adults aged ≥ 18 years had no T2DM (HbA1c < 6.5%) but were either obese (BMI ≥ 30 kg/m^2^) or overweight (BMI: 27 to < 30 kg/m^2^) with at least one coexisting condition (e.g., hypertension, dyslipidemia), and had maintained stable weight (≤ 5% change) for 3 months.	Change from baseline in ALT, AST, ALP, pancreatic lipase and pancreatic amylase at Week 26—GI adverse events.	(222/50)	36 weeks of OFG treatment and a 2‐week post‐treatment follow‐up (38 weeks)	OFG resulted in weight reduction, with AEs similar to injectable GLP‐1RAs
Frias et al. 2023 [17]	RCT, multicenter	NCT05048719	USA, Hungary, Poland and Slovakia	**Oral** OFG (3, 12, 24, 36, or 45 mg maintenance doses) were administered. OFG 36 and 45 mg cohorts were divided into subgroups to test different initial doses and escalation speeds, and all participants received lifestyle and safety education.	Placebo once daily and followed the same dose escalation schedule as the OFG groups	Participants aged ≥ 18 years with T2DM were included if their HbA1c was 7.0%–10.5%. They were on diet/exercise ± stable metformin (≥ 3 months), and had a BMI ≥ 23 kg/m^2^ and stable weight (≤ 5% change) for 3 months.	Change from baseline in ALT, AST, ALP, P‐AMY and pancreatic lipase at Week 26—GI adverse events.	(278/55)	40‐week treatment period and a 2‐week post‐treatment follow‐up (42 weeks)	OFG (≥ 12 mg) lowered HbA1c and weight with an AEs profile similar to other GLP‐1RAs, positioning it as a potentially easier oral treatment for T2DM
Pratt et al. 2023 (Phase 1B) [31]	RCT, multicenter	NCT04426474	USA and Germany	**Oral** OFG was given once daily, with an initial cohort escalating weekly from 3 mg up to 21 mg over 4 weeks. Subsequent cohorts also started at 3 mg, escalated weekly, reaching maintenance doses of 9, 15, 27, or 45 mg between 4 and 6 weeks	Placebo once daily and followed the same dose escalation schedule as the OFG group.	T2D participants aged ≥ 18 years were eligible if treated with diet/exercise ± stable metformin, with HbA1c 7.0%–10.5%, BMI: 18.5–45 kg/m^2^, and stable weight for 3 months.	Safety and tolerability of multiple doses of OFG in participants with T2D—GI adverse events.	(9/17)	12‐week OFG treatment period and a 1‐ to 2‐week follow‐up period (14 weeks)	OFG reduced HbA1c and weight with typical GLP‐1RAs AEs, providing a safe, effective, once‐daily oral alternative to injectable GLP‐1RAs or peptide oral formulations without water and food restrictions
Wharton et al. 2025 (ATTAIN‐1) [16]	RCT, multicenter	NCT05869903	Brazil, China, India, Japan, Korea, Slovakia, Spain, Taiwan and USA	**Oral** OFG was administered at dosages of 6, 12 and 36 mg once daily for 72 weeks, focusing on weight management and lifestyle modifications	Placebo once daily to allow a clear assessment of OFG's efficacy and safety against a non‐active treatment for weight loss and health outcomes.	Adults aged ≥ 18 years were eligible if they were obese (BMI ≥ 30 kg/m^2^) or overweight (BMI: 27–30 kg/m^2^) with an obesity‐related condition (e.g., hypertension), and had a history of unsuccessful dietary weight‐loss attempts.	Change in body weight from baseline to Week 72—GI adverse events.	(1455/949)	72 weeks of OFG treatment and had an additional 2‐week safety follow‐up (74 weeks)	OFG caused significantly greater weight loss vs. placebo in adults with obesity, with a typical GLP‐1 RAs AEs profile

Abbreviations: ALP, serum alkaline phosphatase; ALT, alanine aminotransferase; AST, aspartate aminotransferase; BMI, body mass index; GI, gastrointestinal; GLP‐1RAs, glucagon‐like peptide‐1 receptor agonists; HA1c, haemoglobin, OFG, orforglipron; P‐AMY, pancreatic amylase; RCT; randomized controlled trial; T2DM, type 2 diabetes mellitus.

* Included doses of OFG: 3, 12, 24, 36 and 45 mg, along with placebo only.

**TABLE 2:** Baseline characteristics of the included studies.*

Study ID	Groups	Number of participants	Age (year)	Male, *n*(%)	Weight (kg)	Race/Ethnicity, *n* (%)					Pancreatic & Liver enzyme (IU/L)					Duration of T2DM (year)
						AI/AN	Asian	B/AA	H/L	White	ALT	AST	P‐AMY	ALP	Serum lipase	
Rosenstock et al. 2025 (ACHIEVE‐1) [13]	OFG 3 mg	143	53.3 ± 11.3	80 (56)	90.3 ± 25.7	35 (24.5)	63 (44.1)	8 (5.6)	57 (39.9)	37 (25.9)	26.1 ± 14.4^**†**^	22.8 ± 9.6^**†**^	23.6 ± 10.8^**†**^	NA	33.7 ± 15.6^**†**^	4.0 ± 4.8
	OFG 12 mg	137	54.1 ± 11.8	66 (48)	90.6 ± 23.1	38 (27.7)	59 (43.1)	9 (6.6)	55 (40.1)	30 (21.9)	23.0 ± 12.9^**†**^	20.7 ± 9.4^**†**^	22.5 ± 10.5^**†**^	NA	32.5 ± 15.2^**†**^	5.1 ± 6.0
	OFG 36 mg	141	52.8 ± 11.8	69 (49)	90.1 ± 22.9	35 (24.8)	62 (44.0)	4 (2.8)	55 (39.0)	40 (28.4)	25.0 ± 14.3^**†**^	22.2 ± 9.5^**†**^	22.5 ± 9.5^**†**^	NA	34.2 ± 15.4^**†**^	4.2 ± 5.1
	Placebo	138	53.3 ± 12.5	75 (54)	90.0 ± 20.7	35 (25.4)	61 (44.2)	3 (2.2)	56 (40.6)	38 (27.5)	25.0 ± 14.1^**†**^	21.8 ± 9.4^**†**^	22.5 ± 9.4^**†**^	NA	32.4 ± 15.3^**†**^	4.4 ± 5.6
Wharton et al. 2023 (GZGI) [15]	OFG 12 mg	50	49.8 ± 10.5	19 (38)	107.5 ± 25.3	0 (0)	0 (0)	3 (6)	NA	47 (94)	24.3 ± 12.7^**†**^	19.9 ± 7.1^**†**^	23.9 ± 13.4^**†**^	82.2 ± 24.0^**†**^	30.8 ± 14.9^**†**^	NA
	OFG 24 mg	53	57.0 ± 9.1	23 (43)	112.1 ± 30.2	1 (2)	0 (0)	6 (11)	NA	46 (87)	24.3 ± 12.4^**†**^	20.9 ± 7.8^**†**^	24.90 ± 13.9^**†**^	84 ± 24.8^**†**^	32.2 ± 17.0^**†**^	NA
	OFG 36 mg	58	55.9 ± 11.3	22 (38)	108.3 ± 25.45	0 (0)	0 (0)	8 (14)	NA	25 (86)	23.2 ± 11.8^**†**^	20.8 ± 7.3^**†**^	24.5 ± 12.6^**†**^	79.8 ± 22.2^**†**^	33.4 ± 16.1^**†**^	NA
	OFG 45 mg	61	53.8 ± 11.91	26 (43)	108 ± 24.5	0 (0)	0 (0)	1 (1.64)	NA	59 (97)	22.9 ± 11.7^**†**^	19.42 ± 6.7^**†**^	23.7 ± 11.9^**†**^	81.94 ± 24.5^**†**^	31.9 ± 14.9^**†**^	NA
	Placebo	50	54.0 ± 8.8	21 (42)	107.6 ± 25.2	0 (0)	2 (4)	1 (2)	NA	45 (90)	24.7 ± 12.7^**†**^	20.1 ± 7.07^**†**^	22.8 ± 12.0^**†**^	87.2 ± 24.0^**†**^	30.3 ± 14.9^**†**^	NA
Frias et al. 2023 [17]	OFG 3 mg	51	59.0 ± 9.4	26 (51)	99.3 ± 25.4	0 (0)	1 (2)	2 (4)	7 (14)	47 (92)	26.8 ± 14.5	20.8 ± 8.6	22.0 ± 11.1	77.2 ± 26.3	35.4 ± 19.8	6.6 ± 6.9
	OFG 12 mg	56	57.4 ± 9.2	36 (64)	99.3 ± 18.1	1 (2)	1 (2)	5 (9)	15 (27)	49 (88)	28.5 ± 15.7	22.2 ± 9.4	24.7 ± 12.8	74.3 ± 25.7	38.0 ± 21.9	7.8 ± 6.8
	OFG 24 mg	47	60.5 ± 9.1	30 (64)	98.5 ± 22.9	1 (2)	1 (2)	2 (4)	5 (11)	43 (91)	25.1 ± 13.8	21.1 ± 8.8	25.3 ± 13.0	69.9 ± 24.1	39.7 ± 22.7	6.4 ± 5.1
	OFG 36 mg	61	59.7 ± 9.2	36 (59)	98.9 ± 17.5	0 (0)	1 (2)	0 (0)	13 (21)	58 (95)	26.0 ± 14.3	21.3 ± 8.9	23.4 ± 12.2	75.1 ± 26.0	36.4 ± 21.0	6.1 ± 4.7
	OFG 45 mg	63	58.5 ± 9.4	40 (63)	104.6 ± 25.1	1 (2)	0 (0)	5 (8)	13 (21)	57 (90)	25.7 ± 13.9	20.4 ± 8.5	22.1 ± 11.4	78·8 ± 26.8	36.9 ± 21.0	6.8 ± 5.8
	Placebo	55	58.3 ± 9.5	28 (51)	102.0 ± 18.8	1 (2)	0 (0)	4 (7)	14 (25)	50 (91)	28.4 ± 15.4	22.5 ± 9.3	20.6 ± 10.6	76.2 ± 25.9	37.3 ± 21.3	8.1 ± 6.5
Pratt et al. 2023 (Phase 1B) [31]	OFG 45 mg	9	62.8 ± 4.4	4 (44.4)	81.5 ± 10.24	NA	NA	NA	NA	NA	23.3 ± 11.5	16.7 ± 7.2	58.8 ± 15.3	NA	35.9 ± 16.0	10.4 ± 4.8
	Placebo	17	56.0 ± 6.0	10 (58.8)	90.3 ± 20.04	NA	NA	NA	NA	NA	23.9 ± 7.7	18.2 ± 4.5	63.7 ± 22.7	NA	42.7 ± 13.2	8.6 ± 4.9
Wharton et al. 2025 (ATTAIN‐1) [16]	OFG 12 mg	725	45.4 ± 12.6	258 (35.6)	102.2 ± 21.6	NA	201 (28.1)	60 (8.4)	275 (37.9)	405 (56.6)	22.7 ± 13.5^**§**^	20.3 ± 8.1^**§**^	23.4 ± 8.1^**§**^	NA	29.9 ± 10.8^**§**^	NA
	OFG 36 mg	730	44.9 ± 11.9	265 (36.3)	103.1 ± 23.2	NA	214 (29.6)	67 (9.3)	258 (35.3)	394 (54.4)	22.8 ± 13.5^**§**^	20.1 ± 8.1^**§**^	23.2 ± 8.1^**§**^	NA	28.7 ± 10.8^**§**^	NA
	Placebo	949	45.1 ± 11.9	341 (35.9)	103.9 ± 22.0	NA	267 (28.5)	72 (7.7)	369 (38.9)	539 (57.5)	22.7 ± 12.3^**§**^	20.4 ± 6.2^**§**^	23.5 ± 9.2^**§**^	NA	28.7 ± 9.2^**§**^	NA

Abbreviations: AI/AN, American Indian or Alaska Native; ALP, serum alkaline phosphatase; ALT, alanine aminotransferase; AST, aspartate aminotransferase; B/AA, Black or African American; H/L, Hispanic or Latino; NA, not applicable; OFG, orforglipron; P‐AMY, pancreatic amylase; T2DM, type 2 diabetes mellitus.

* Plus–minus values are means ± SD

^**†**^ LSM ± SD; least square mean ± standard deviation

^**§**^ MBE ± SD; Model‐Based Estimates ± standard deviation.

### 3.3 Risk of Bias Assessment

**FIGURE 2:**
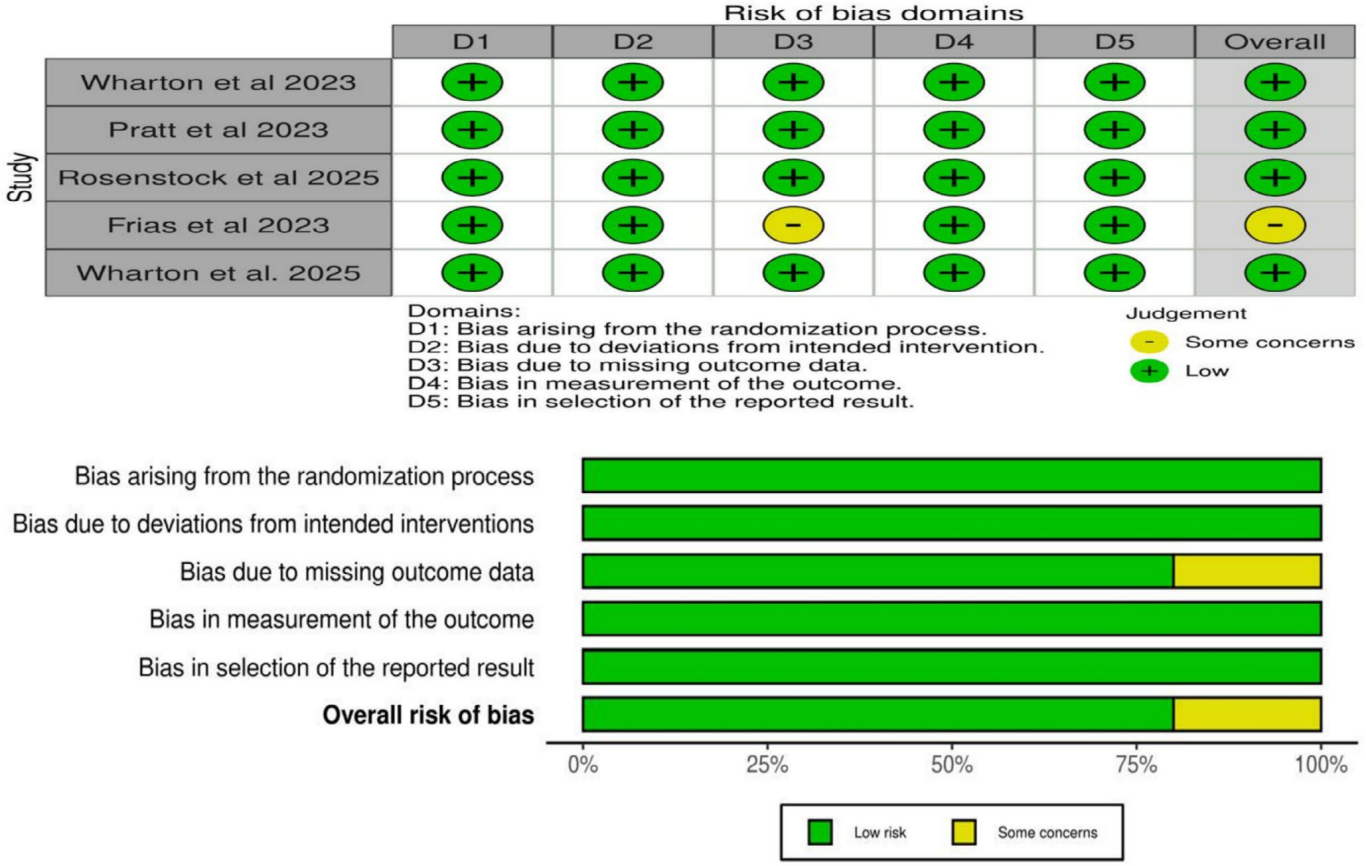
Quality assessment of risk of bias in the included trials. The upper panel presents a schematic representation of risks (low = green and some concerns = yellow) for specific types of biases of each study in the review. The lower panel presents risks (low = green and some concerns = yellow) for the subtypes of biases of the combination of studies included in this review.

Overall, four RCTs were rated as having a low risk of bias [13, 15, 31], while one RCT was rated as having some concerns [17] (Figure 2). The source of bias in Frias et al. [17] is mainly related to the uneven distribution of patient loss, which occurred in three groups (Domain 3: bias due to missing outcome data). All studies have shown a low risk due to adequate sequence generation and balanced baselines.

### 3.4 Meta‐Analysis

#### 3.4.1 Gastrointestinal Adverse Events

GI AEs leading to discontinuation, nausea, vomiting, diarrhoea, dyspepsia and eructation were evaluated in five studies involving 3603 patients, and six doses were assessed. Four studies and six doses evaluated constipation in 3577 patients, decreased appetite in 3257 patients and hepatic events in 3044 patients. Three studies and six doses were assessed for gastroesophageal reflux disease (GERD) in 3231 patients, abdominal pain in 3231 patients, abdominal distension in 2985 patients and pancreatitis in 3305 patients.

All evaluated OFG doses (3, 12, 24, 36 and 45 mg) were associated with significantly increased odds of gastrointestinal adverse events compared with placebo (*p* < 0.05), including GI AEs leading to discontinuation (Figure S1), nausea (Figure S2), vomiting (Figure S3), diarrhoea (Figure S4), eructation (Figure S5) and constipation (Figure S6).

Importantly, most GI AEs were mild to moderate in severity and did not lead to treatment discontinuation. Nausea, vomiting, diarrhoea, constipation, dyspepsia and eructation demonstrated a consistent dose–response relationship, with progressively higher odds observed at doses ≥ 12 mg. Although discontinuations due to GI AEs increased at higher doses, the majority of reported GI events were managed conservatively and did not necessitate treatment withdrawal.

The 12 and 36 mg doses were associated with significantly increased odds of abdominal pain compared to placebo (OR: 2.36, 95% CI: [1.49; 3.76], *p* = 0.0003, SUCRA: 0.34; and OR: 2.63, 95% CI: [1.67; 4.15], *p* < 0.0001, SUCRA: 0.24, respectively; Figure S7). Similarly, both doses were associated with higher odds of GERD relative to placebo (OR: 2.86, 95% CI: [1.75; 4.67], *p* < 0.0001, SUCRA: 0.37; and OR: 3.08, 95% CI: [1.89; 5.00], *p* < 0.0001, SUCRA: 0.29, respectively; Figure S9). In contrast, the 3, 24 and 45 mg doses did not reach statistical significance for either abdominal pain or GERD.

The 12, 24, 36 and 45 mg doses were demonstrated significantly higher odds of decreased appetite (Figure S8) and dyspepsia (Figure S10) compared with placebo (*p* < 0.05), whereas no statistically significant association was observed for the 3‐mg dose (*p* > 0.05). However, all doses (3, 12, 24, 36 and 45 mg) showed not significant differences compared to placebo in the odds of pancreatitis (Figure S11), abdominal distension (Figure S12), or hepatic events (Figure S13).

A network graph summarizing all direct comparisons between OFG doses in our study regarding safety outcomes at Week 26 is illustrated in (Figure S14).

There were no observed heterogeneities in all GI adverse events (*I* ^2^ = 0%), except for discontinuation due to GI adverse events (*I* ^2^ = 12.2%), diarrhoea (*I* ^2^ = 38.2%) and abdominal distention (*I* ^2^ = 75.8%). A network graph summarizing all direct comparisons between OFG doses in our study regarding safety outcomes at Week 26 is illustrated in (Figure S14).

#### 3.4.2 Percentage (%) Change From Baseline in Enzymes at Week 26 IU/L

The network meta‐analysis evaluated the effect of different doses of OFG on the % change from baseline in enzymes at Week 26. ALT, AST, total serum lipase, serum alkaline phosphatase were assessed.

**FIGURE 3:**
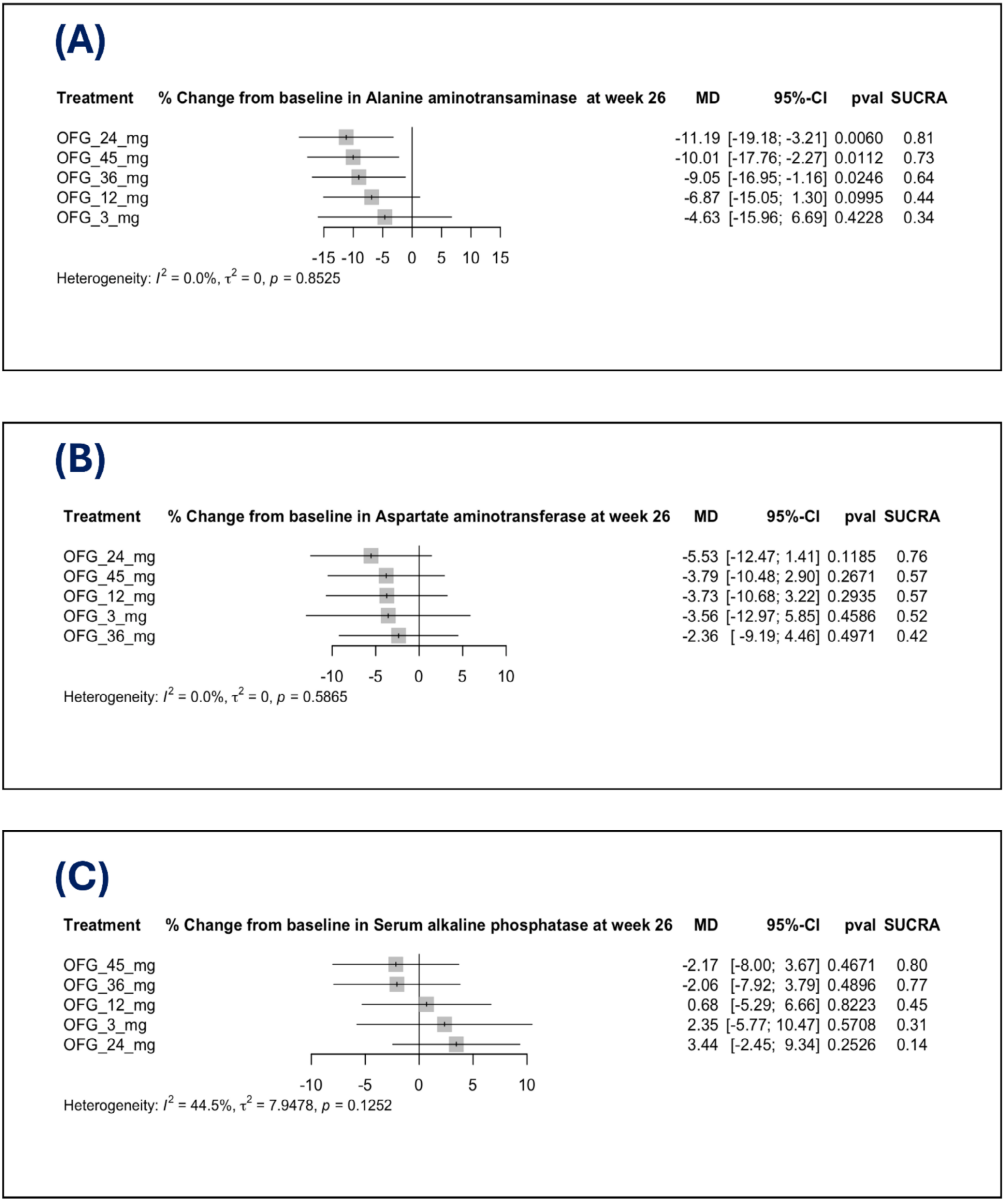
Forest plots for percentage (%) change from baseline hepatic enzymes at Week 26: (A) % change from baseline in ALT, (B) % change from baseline in AST, (C) % change from baseline in ALP.

Doses of 24, 45 and 36 mg resulted in a significantly greater reduction in ALT compared to the placebo. The 24 mg dose showed the most significant decrease in ALT (MD: −11.19, 95% CI: [−19.18; −3.21], *p* = 0.0060; SUCRA: 0.81), followed by 45 mg (MD: −10.01, 95% CI: [−17.76; −2.27], *p* = 0.0112; SUCRA: 0.73) and 36 mg (MD: −9.05, 95% CI: [−16.95; −1.16], *p* = 0.0246; SUCRA: 0.64) (Figure 3).

**FIGURE 4:**
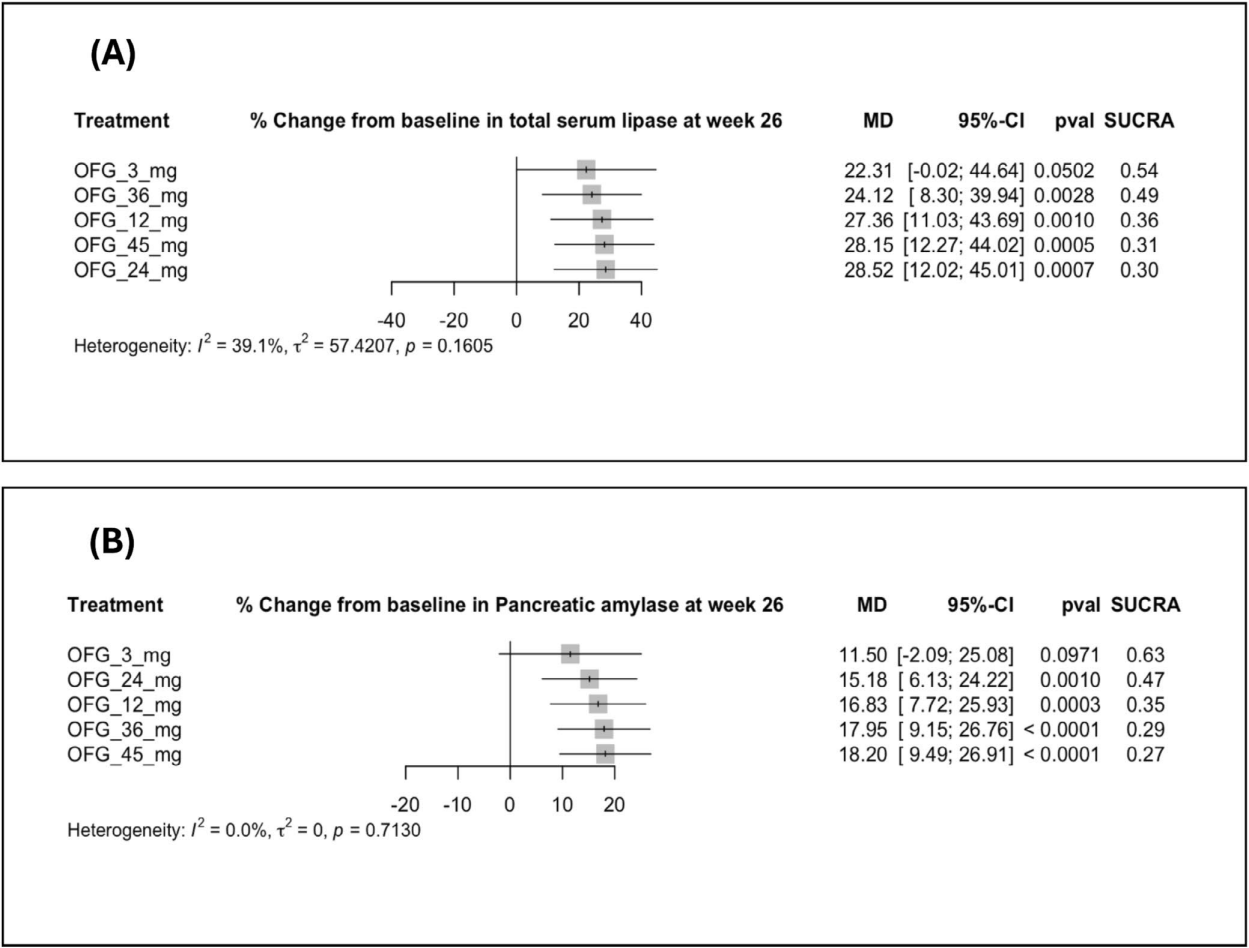
Forest plots for percentage (%) change from baseline in pancreatic enzymes at Week 26: (A) % change from baseline in total serum lipase, (B) % change from baseline in pancreatic amylase.

Doses of 12, 24, 36 and 45 mg increased total serum lipase (Figure 4) and pancreatic amylase (Figure 4) compared with the placebo. In contrast, the 3 mg dose did not reach statistical significance. Total serum lipase rose with 24 mg (MD: 28.52, 95% CI: [12.02; 45.01], *p* = 0.0007; SUCRA: 0.30), 45 mg (MD: 28.15, 95% CI: [12.27; 44.02], *p* = 0.0005; SUCRA: 0.31) and 12 mg (MD: 27.36, 95% CI: [11.03; 43.69], *p* = 0.0010; SUCRA: 0.36). Pancreatic amylase increased with 45 mg (MD: 18.20, 95% CI: [9.49; 26.91], *p* < 0.0001; SUCRA: 0.27), 36 mg (MD: 17.95, 95% CI: [9.15; 26.76], *p* < 0.0001; SUCRA: 0.29) and 12 mg (MD: 16.83, 95% CI: [7.72; 25.93], *p* = 0.0003; SUCRA: 0.35) (Figure 4). However, all doses showed no significant differences compared to the placebo in the odds of ALP (Figure 3) and AST (Figure 3).

**FIGURE 5:**
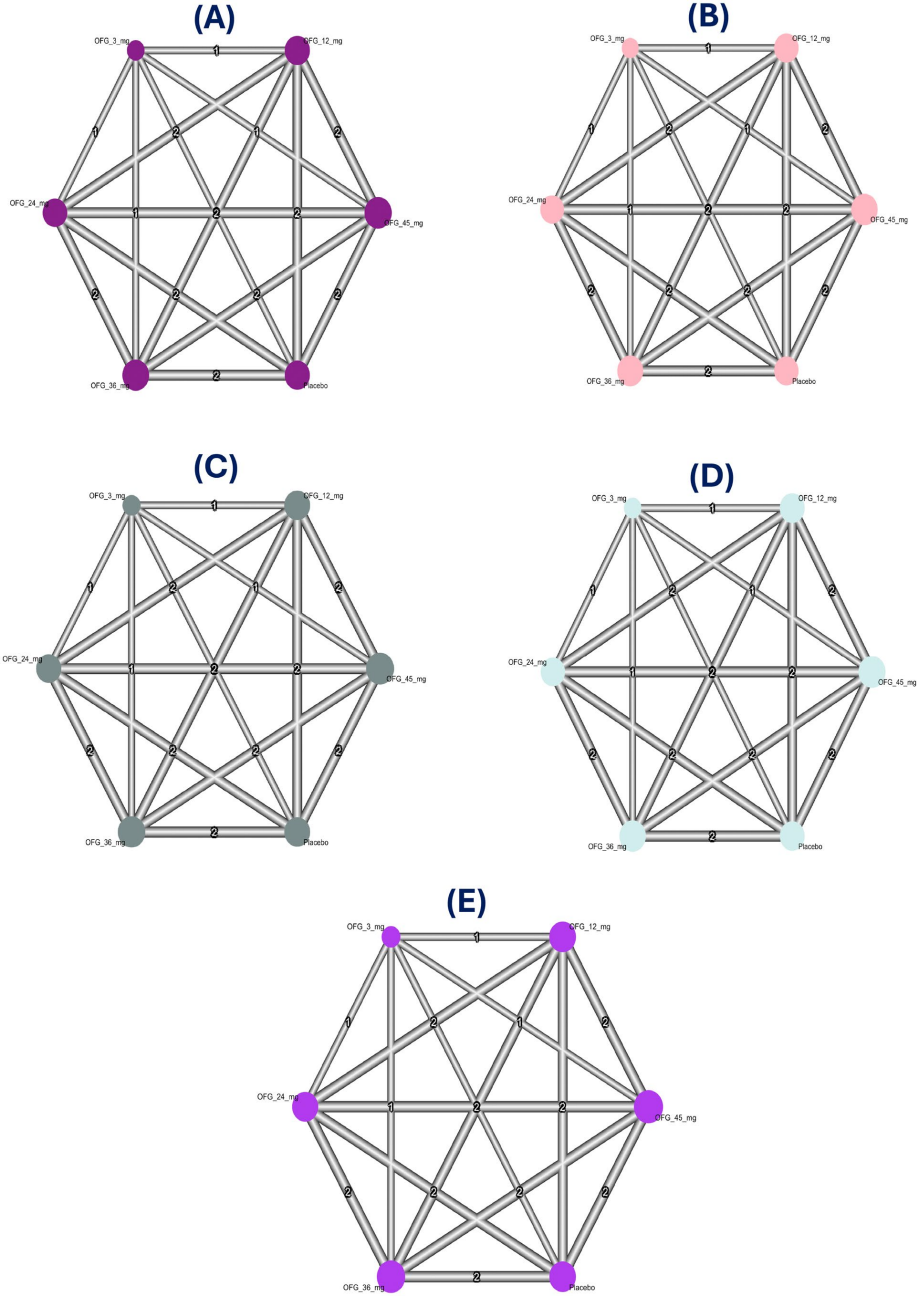
Network graphs for % change from baseline in hepatic and pancreatic enzymes at Week 26: (A) % change from baseline in ALT, (B) % change from baseline in AST, (C) % change from baseline in ALP, (D) % change from baseline in total serum lipase, (E) % change from baseline in pancreatic amylase.

There was no observed heterogeneity for pancreatic amylase (*I* ^2^ = 0%), while the moderate heterogeneity for total serum lipase (*I* ^2^ = 39.1%) (Figure 4), and for ALP (*I* ^2^ = 44.5%) (Figure 3). A network graph summarizing all direct comparisons between OFG doses in our study regarding %change from baseline in hepatic and pancreatic enzymes at Week 26 is illustrated in (Figure 5). All pairwise comparisons of % changes in enzymes at Week 26 are demonstrated in (Table 3).

**TABLE 3:** League table of network meta‐analysis displaying relative OFG effects for all pairwise comparisons in % change in hepatic and pancreatic enzymes at Week 26.

**(A) % Change in ALT at Week 26**					
OFG 36 mg	2.22 (−5.10; 9.54)	0.94 (−6.11; 8.00)	−2.21 (−9.74; 5.32)	−4.90 (−17.03; 7.23)	−9.05 (−16.95; −1.16)
2.14 (−5.18; 9.46)	OFG 24 mg	−1.18 (−8.33; 5.97)	−4.32 (−11.94; 3.29)	−9.60 (−22.08; 2.88)	−11.27 (−19.25; −3.28)
0.96 (−6.09; 8.01)	−1.18 (−8.33; 5.97)	OFG 45 mg	−3.14 (−10.50; 4.22)	−4.10 (−16.15; 7.95)	−9.98 (−17.72; −2.24)
−2.18 (−9.71; 5.34)	−4.32 (−11.94; 3.30)	−3.14 (−10.51; 4.22)	OFG 12 mg	−0.10 (−12.86; 12.66)	−6.82 (−15.00; 1.35)
−4.42 (−15.28; 6.44)	−6.56 (−17.53; 4.41)	−5.38 (−16.17; 5.41)	−2.24 (−13.34; 8.86)	OFG 3 mg	−4.80 (−17.92; 8.32)
**−9.05 (−16.95; −1.16)**	−11.19 (−19.18; −3.21)	−10.01 (−17.76; −2.27)	−6.87 (−15.05; 1.30)	−4.63 (−15.96; 6.69)	Placebo
**(B) % Change in AST at Week 26**					
OFG 36 mg	3.15 (−3.51; 9.82)	1.42 (−4.97; 7.82)	1.38 (−5.29; 8.05)	3.40 (−7.09; 13.89)	−2.32 (−9.15; 4.50)
3.17 (−3.50; 9.83)	OFG 24 mg	−1.72 (−8.25; 4.80)	−1.80 (−8.59; 4.99)	−6.00 (−16.63; 4.63)	−5.60 (−12.55; 1.34)
1.42 (−4.97; 7.82)	−1.74 (−8.27; 4.78)	OFG 45 mg	−0.05 (−6.59; 6.48)	0.80 (−9.48; 11.08)	−3.77 (−10.47; 2.92)
1.36 (−5.31; 8.03)	−1.81 (−8.60; 4.99)	−0.06 (−6.60; 6.47)	OFG 12 mg	0.90 (−9.76; 11.56)	−3.72 (−10.67; 3.24)
1.20 (−8.04; 10.44)	−1.97 (−11.29; 7.35)	−0.23 (−9.37; 8.91)	−0.17 (−9.49; 9.16)	OFG 3 mg	−3.00 (−13.76; 7.76)
−2.36 (−9.19; 4.46)	−5.53 (−12.47; 1.41)	−3.79 (−10.48; 2.90)	−3.73 (−10.68; 3.22)	−3.56 (−12.97; 5.85)	Placebo
**(C) % Change in ALP at Week 26**					
OFG 36 mg	−5.47 (−11.23; 0.29)	0.10 (−5.60; 5.80)	−2.78 (−8.62; 3.05)	−4.50 (−13.49; 4.49)	−2.06 (−7.92; 3.79)
−5.51 (−11.26; 0.25)	OFG 24 mg	5.60 (−0.14; 11.34)	2.76 (−3.12; 8.63)	4.30 (−4.69; 13.29)	3.41 (−2.49; 9.31)
0.10 (−5.60; 5.80)	5.61 (−0.13; 11.35)	OFG 45 mg	−2.86 (−8.67; 2.96)	−4.60 (−13.52; 4.32)	−2.17 (−8.01; 3.67)
−2.75 (−8.58; 3.09)	2.76 (−3.12; 8.63)	−2.85 (−8.67; 2.96)	OFG 12 mg	−5.10 (−14.21; 4.01)	0.71 (−5.26; 6.68)
−4.41 (−12.43; 3.61)	1.09 (−6.94; 9.13)	−4.51 (−12.51; 3.48)	−1.66 (−9.76; 6.43)	OFG 3 mg	2.10 (−7.08; 11.28)
−2.06 (−7.92; 3.79)	3.44 (−2.45; 9.34)	−2.17 (−8.00; 3.67)	0.68 (−5.29; 6.66)	2.35 (−5.77; 10.47)	Placebo
**(D) % Change in P‐AMY at Week 26**					
OFG 36 mg	2.74 (−6.77; 12.26)	−0.33 (−9.52; 8.86)	1.05 (−8.52; 10.61)	7.50 (−8.69; 23.69)	17.95 (9.14; 26.76)
2.78 (−6.73; 12.28)	OFG 24 mg	−3.25 (−12.68; 6.19)	−1.58 (−11.37; 8.20)	3.80 (−13.23; 20.83)	15.14 (6.09; 24.19)
−0.25 (−9.43; 8.94)	−3.02 (−12.44; 6.40)	OFG 45 mg	1.37 (−8.12; 10.85)	2.70 (−12.99; 18.39)	18.29 (9.58; 27.00)
1.12 (−8.44; 10.69)	−1.65 (−11.43; 8.13)	1.37 (−8.11; 10.85)	OFG 12 mg	10.90 (−6.00; 27.80)	16.89 (7.78; 26.00)
6.45 (−7.46; 20.37)	3.68 (−10.44; 17.80)	6.70 (−7.09; 20.49)	5.33 (−8.80; 19.46)	OFG 3 mg	12.50 (−2.80; 27.80)
17.95 (9.15; 26.76)	15.18 (6.13; 24.22)	18.20 (9.49; 26.91)	16.83 (7.72; 25.93)	11.50 (−2.09; 25.08)	Placebo
**(E) % Change in serum lipase at Week 26**					
OFG 36 mg	−4.72 (−21.87; 12.43)	−4.56 (−21.12; 12.00)	−3.30 (−20.28; 13.68)	3.20 (−22.67; 29.07)	24.16 (8.33; 39.98)
−4.40 (−21.53; 12.74)	OFG 24 mg	−0.41 (−17.68; 16.87)	1.52 (−16.08; 19.13)	12.60 (−15.26; 40.46)	28.93 (12.41; 45.44)
−4.02 (−20.57; 12.52)	0.37 (−16.83; 17.58)	OFG 45 mg	1.34 (−15.72; 18.40)	−4.90 (−30.07; 20.27)	28.50 (12.62; 44.38)
−3.23 (−20.21; 13.74)	1.16 (−16.44; 18.76)	0.79 (−16.24; 17.82)	OFG 12 mg	9.30 (−17.53; 36.13)	27.48 (11.15; 43.82)
1.81 (−21.03; 24.65)	6.21 (−17.26; 29.67)	5.83 (−16.88; 28.55)	5.04 (−18.19; 28.28)	OFG 3 mg	20.50 (−4.22; 45.22)
**24.12 (8.30; 39.94)**	28.52 (12.02; 45.01)	28.15 (12.27; 44.02)	27.36 (11.03; 43.69)	22.31 (−0.02; 44.64)	Placebo

*Note:* All values are presented as MD with 95% CI; bolded values are significant finding in the row.

Abbreviations: ALP, alkaline phosphatase; ALT, alanine aminotransferase; AST, aspartate aminotransferase; CI, confidence interval; MD, mean difference; OFG, orforglipron; P‐AMY, pancreatic amylase.

#### 3.4.3 Subgroup Analysis

Subgroup analyses were conducted to explore potential differences in treatment effects according to diabetes status (patients with and without DM). Comparisons were made among various OFG dose groups (12, 24, 36 and 45 mg) and placebo, using random‐effects models.

Subgroup analysis showed no significant difference in all GI AEs, including GI AEs leading to discontinuation (Figure S15), nausea (Figure S16), vomiting (Figure S17), diarrhoea (Figure S18), dyspepsia (Figure S19), eructation (Figure S20), constipation (Figure S21), decreased appetite (Figure S22), hepatic events (Figure S23), GERD (Figure S24), abdominal pain (Figure S25) and pancreatitis (Figure S26). There were no significant interactions between the subgroup and treatment (all *p* > 0.05).

There were significant differences in abdominal distension between patients with and without DM when comparing the 12 mg dose to placebo (*Q* = 8.20, *p* = 0.0042). Participants without DM had higher odds of abdominal distension with both OFG doses compared to placebo. In contrast, those with DM generally had neutral or slightly better responses, although most confidence intervals were wide due to the data being from single‐study subgroups. The OFG 12 mg vs. placebo comparison showed significant heterogeneity, suggesting that DM status may affect treatment outcomes at this dose (Figure S27).

For ALT, AST, pancreatic amylase and pancreatic lipase, all comparisons showed no significant subgroup–treatment interactions (all *p* > 0.05); however, the comparison of 45 mg versus placebo in patients without diabetes revealed a statistically significant increase in serum lipase. This means treatment effects were generally similar for patients with and without DM (Figures S28, S29, S30 and S31, respectively).

ALP levels were not significantly affected by diabetes status in terms of treatment effect. Some subgroup differences were observed, primarily in ALP; however, this was inconsistent, derived from single studies, and lacked clinical significance. Overall, the subgroup results show that diabetes did not influence the safety‐related biochemical responses to OFG treatment at Week 26. (Figure S32).

Subgroup analyses for ALP resulted in no significant differences between diabetic and non‐diabetic participants for all dose and placebo comparisons (all *p* > 0.05). Although comparisons of OFG 12 mg vs. OFG 24 mg showed statistically significant between‐subgroup heterogeneity (*Q* = 3.93, *p* = 0.0474). Among patients with diabetes, OFG 12 mg vs. OFG 24 mg demonstrated a significant reduction in ALP (MD: −9.40, 95% CI: [−16.18; −2.62]) (Figure S32).

#### 3.4.4 Inconsistency

A design‐by‐treatment analysis did not reveal any significant global inconsistency (all *p* > 0.05) for GI AEs leading to discontinuation (Figure S33), nausea (Figure S34), vomiting (Figure S35), diarrhoea (Figure S36), dyspepsia (Figure S37), eructation (Figure S38), constipation (Figure S39), decreased appetite (Figure S40), hepatic events (Figure S41), GERD (Figure S42), abdominal pain (Figure S43), pancreatitis (Figure S44), ALT, AST, pancreatic amylase, total serum lipase and ALP (Figures S45, S46, S47, S48 and S49, respectively). However, global inconsistency was significant for abdominal distension (*p* = 0.0161). The node‐splitting method revealed no significant evidence of inconsistency for any of the outcomes (*p*‐value > 0.05). Only nausea showed significant inconsistency in comparison between OFG 24 mg and OFG 45 mg (*p* = 0.0494).

## 4 Discussion

The present network meta‐analysis provides a comprehensive assessment of gastrointestinal, hepatic and pancreatic safety across a wide range of OFG doses in adults with or without T2DM. All active doses increased the odds of GI AEs versus placebo, with a clear dose–response pattern for nausea, vomiting, diarrhoea, constipation, dyspepsia, eructation and discontinuation due to GI AEs. Notably, while discontinuations increased at higher doses, most GI AEs were transient and did not result in treatment cessation, reflecting a tolerability profile consistent with other GLP‐1 RAs. In contrast, the odds of pancreatitis, abdominal distension and hepatic events were not significantly different from those of the placebo, despite dose‐related increases in lipase and pancreatic amylase and modest dose‐dependent reductions in ALT. Subgroup analyses showed broadly similar safety profiles in patients with and without DM, with only isolated heterogeneity signals for abdominal distension. These observations indicate that OFG reproduces the class‐typical tolerability pattern of GLP‐1 RAs, without emergent signals of hepatotoxicity or clinically relevant pancreatic injury over 26 weeks.

The excess of GI AEs across all OFG doses is consistent with the known pharmacology of GLP‐1 RAs and with phase 2–3 OFG trials, where nausea, vomiting and diarrhoea were the leading causes of discontinuation [16, 31, 32]. GLP‐1–mediated delayed gastric emptying and central satiety likely underlie these events. The gradient observed in our analysis, with lower odds at 3 mg and progressively higher odds at 12–45 mg, mirrors dose–response patterns seen with semaglutide, liraglutide and tirzepatide, as well as with other oral small‐molecule GLP‐1 RAs, such as danuglipron and lotiglipron [14, 33, 34]. The higher SUCRA rankings for some intermediate doses (e.g., 36 mg for diarrhoea and 45 mg for constipation and eructation) suggest that side effects do not always increase linearly with higher doses. This pattern is likely related to differences in dose‐escalation schemes, patient characteristics and the reporting of adverse events, rather than true biological differences between doses. Clinically, this supports the use of slow dose escalation and tailoring the final dose to each patient to achieve weight loss while keeping side effects manageable.

For outcomes, such as abdominal pain, GERD and decreased appetite, the 3 mg dose showed odds similar to those of the placebo, whereas higher doses modestly increased the risk. Appetite suppression is a desired pharmacodynamic effect, closely linked to weight reduction, but GERD and abdominal pain can limit adherence. The pattern in our network is congruent with prior GLP‐1 RA meta‐analyses, which consistently report increased upper GI symptoms but no major safety concerns beyond tolerability [35, 36, 37].

Hepatic safety is a key question for oral GLP‐1 RAs, particularly after the discontinuation of danuglipron and lotiglipron programs following liver enzyme elevations and a potential drug‐induced liver injury case [34, 38]. In this context, the absence of a signal for excess hepatic events across all OFG doses is reassuring. Higher doses (24–45 mg) were associated with statistically significant reductions in ALT, while AST remained unchanged. This pattern is consistent with meta‐analyses showing that GLP‐1 RAs improve liver enzymes, hepatic fat, and, in some cases, histology in patients with MASLD or NASH, likely through weight loss, enhanced insulin sensitivity and reduced hepatic inflammation [39, 40, 41]. Our findings, therefore, align more closely with the beneficial hepatic effects reported for injectable GLP‐1 RAs than with the hepatotoxic profile that halted some oral small‐molecule programs. However, the short follow‐up and selective trial populations mean that rare idiosyncratic liver injury cannot be excluded.

Pancreatic safety is another central concern. In our analysis, all doses ≥ 12 mg increased lipase and pancreatic amylase levels, but none increased the odds of clinically adjudicated pancreatitis compared with the placebo. This dissociation aligns with multiple recent meta‐analyses involving tens of thousands of patients, which collectively show no significant increase in acute pancreatitis risk with GLP‐1 RAs overall, despite modest and reversible enzyme elevations [10, 11, 42]. Observational studies focused on high‐risk populations with a history of pancreatitis similarly do not demonstrate higher recurrence rates with GLP‐1 RAs [10, 43, 44]. Mechanistically, GLP‐1 receptor expression in ductal and acinar tissue may increase enzyme synthesis without inducing clinically relevant inflammation. Concurrent weight loss may ultimately reduce the risk of pancreatitis through improvements in triglyceride levels, insulin resistance and gallstone burden.

At the same time, GLP‐1 RAs have been associated with an increased risk of gallbladder and biliary disease, particularly at higher doses and when used for weight loss, as shown in a large meta‐analysis by He et al. and subsequent updates [12, 45, 46]. Although gallbladder‐related events, including cholecystectomy, are commonly reported in individual trials, their definitions, ascertainment methods, and reporting thresholds varied substantially across studies. As a result, these outcomes could not be harmonized or reliably incorporated into our NMA. Therefore, the neutral pancreatitis signal observed in our analysis should be interpreted cautiously and should not be taken to exclude an increased risk of biliary disease associated with GLP‐1 RAs.

Subgroup analyses demonstrated that diabetes status did not significantly modify GI, hepatic, or pancreatic responses to OFG, except for abdominal distension at 12 mg. These signals arose from small subgroups with wide CIs and no consistent directional pattern, suggesting chance findings rather than true effect modification.

Network heterogeneity and inconsistency were generally low. Global tests did not detect inconsistency for most GI, hepatic and pancreatic outcomes, and local node‐splitting analyses identified only a single discordant contrast for nausea. The higher heterogeneity observed for abdominal distension likely reflects sparse data and between‐study differences rather than true clinical divergence. Overall, the geometry and statistical behaviour of the network support the robustness of the main findings.

### 4.1 Clinical Implications

Current diabetes and obesity guidelines from the ADA, EASD, AACE and obesity societies recommend GLP‐1 RAs or dual GIP/GLP‐1 agonists for people with T2DM and high cardiovascular or renal risk, and as first‐line pharmacotherapy in many individuals with obesity who do not achieve sufficient weight loss with lifestyle measures alone [47, 48, 49]. These documents acknowledge GI intolerance, rare pancreatitis and gallbladder events, and thyroid C‐cell warnings, but still consider GLP‐1 RAs to have a favourable benefit–risk profile in appropriate candidates, and recent EASL–EASD–EASO MASLD guidelines similarly view them as promising for improving steatosis and ALT, without yet recommending them solely for liver disease [50]. Within this framework, our findings indicate that OFG behaves like other GLP‐1 RAs, with dose‐related GI AEs, modest ALT reductions at higher doses, pancreatic enzyme elevations without a clear pancreatitis signal, and no evidence of hepatotoxicity over 26 weeks. OFG may therefore be used as an oral alternative for patients who meet guideline criteria for GLP‐1 therapy but prefer non‐injectable treatment, without altering existing cautions regarding pancreatitis or gallbladder disease.

### 4.2 Strengths, Limitations and Recommendations

Our network meta‐analysis has several strengths. It synthesizes data from Phase 1b, 2 and 3 randomized, double‐blind trials of OFG in obese adults with or without T2DM, spanning a broad dose range and follow‐up to 72 weeks. Safety outcomes, including pancreatitis and hepatobiliary events, were prospectively collected and adjudicated in the pivotal studies. A prespecified frequentist network approach, utilizing random‐effects models, SUCRA rankings, and formal heterogeneity and inconsistency checks, provided a consistent analytic framework.

Key limitations should be noted. This study included only five RCTs, which restricts the ability to detect rare AEs and may increase uncertainty around estimates for infrequent outcomes. This reflects the limited availability of randomized evidence on this topic rather than selective study inclusion. Despite this, the pooled analysis provides valuable insights into the primary outcomes using the best available evidence. Future large‐scale RCTs are warranted to better evaluate rare and long‐term AEs. All trials were industry‐sponsored and enrolled selected, relatively low‐risk participants; individuals with prior pancreatitis, advanced liver disease, marked baseline enzyme elevation, or severe renal impairment were explicitly excluded according to trial eligibility criteria. However, these RCTs may limit the generalizability of the findings to higher‐risk populations. As a result, caution is warranted when extrapolating these results to patients with greater baseline risk, multiple comorbidities, or more advanced disease. Future independently funded RCTs that include more diverse and higher‐risk populations are needed to confirm the applicability of these findings across broader clinical settings.

Detailed liver and pancreatic enzyme data were available only for 12–26 weeks, and longer studies reported few clinical hepatic or pancreatic events, limiting conclusions on rare or delayed toxicity. Some dose–outcome estimates relied on small event numbers, and only study‐level data were available. Besides, the low sample size in each comparison should be acknowledged as a limitation that may reduce statistical power and the precision of the estimated effects. Importantly, the observed dissociation between elevations in pancreatic enzyme levels and the occurrence of clinical pancreatitis warrants cautious interpretation. The included trials were not designed to investigate underlying biological mechanisms or to capture long‐term pancreatic outcomes, limiting the ability to draw definitive conclusions regarding causality. Moreover, biliary outcomes were not systematically assessed, representing an important knowledge gap given the known class effects of GLP‐1 RAs. Future studies with longer follow‐up, mechanistic endpoints, and comprehensive evaluation of biliary events are needed to clarify these associations.

Overall, these factors support the use of OFG with the same precautions recommended for other GLP‐1 RAs, especially in patients with hepatobiliary or pancreatic risk factors, and underscore the need for longer‐term, individualized data and head‐to‐head studies in broader, higher‐risk populations. Some outcome data were extracted using WebPlot Digitizer, which may introduce minor measurement imprecision.

## 5 Conclusion

In adults with or without T2DM, OFG increased GI AEs in a dose‐dependent manner but did not raise pancreatitis or hepatic events versus placebo over 26 weeks. Higher doses resulted in modest reductions in ALT and increased pancreatic enzymes, without clear clinical evidence of pancreatic or hepatic injury. Overall, the safety profile is consistent with that of other GLP‐1 RAs and supports the use of OFG as an oral option, with careful dose titration and continued monitoring in patients at higher risk for hepatobiliary or pancreatic issues.

## Author Contributions

**Ahmed W. Hageen:** conceptualization, investigation, writing – original draft, methodology, validation, visualization, writing – review and editing, software, formal analysis, project administration, data curation, supervision, resources. **Ahmed Farid Gadelmawla:** writing – original draft, writing – review and editing. **Amira Fahmy El‐Nemr:** resources, data curation. **Ahmad Omar Saleh:** formal analysis, writing – original draft. **Safir Eladawi:** data curation, resources, project administration. **Abdallfatah Abdallfatah:** writing – original draft, resources. **Ahmed Elsekhary:** resources, data curation. **Biruk Demisse Ayalew:** investigation, writing – original draft, visualization, data curation, project administration. **Odai Maihoub:** resources, data curation. **Mohamed Galal Flefel:** supervision. **Ayoup Ahmed Radi:** data curation, resources. **Mohamed Reyad Mohamed:** writing – original draft, data curation, resources. **Mohamed Abuelazm:** supervision. **Ahmed Mansour:** resources, data curation.

## Funding

The authors have nothing to report.

## Ethics Statement

The authors have nothing to report.

## Consent

The authors have nothing to report.

## Conflicts of Interest

The authors declare no conflicts of interest.

## Supporting information


**Figure S1:** Forest plot for GI AEs leading to discontinuation.
**Figure S2:** Forest plot for nausea.
**Figure S3:** Forest plot for vomiting.
**Figure S4:** Forest plot for diarrhoea.
**Figure S5:** Forest plot for eructation.
**Figure S6:** Forest plot for constipation.
**Figure S7:** Forest plot for abdominal pain.
**Figure S8:** Forest plot for decreased appetite.
**Figure S9:** Forest plot for GERD.
**Figure S10:** Forest plot for dyspepsia.
**Figure S11:** Forest plot for pancreatitis.
**Figure S12:** Forest plot for abdominal distension.
**Figure S13:** Forest plot for hepatic events.
**Figure S14:** Net graphs for safety outcomes: (A) GI AEs leading to discontinuation, (B) nausea, (C) vomiting, (D) diarrhoea, (E) eructation, (F) constipation, (J) abdominal pain, (H) decreased appetite, (I) GERD, (J) dyspepsia, (K) abdominal distension, (L) hepatic events, **(M)** pancreatitis.
**Figure S15:** Subgroup analysis for GI AEs leading to discontinuation.
**Figure S16:** Subgroup analysis for nausea.
**Figure S17:** Subgroup analysis for vomiting.
**Figure S18:** Subgroup analysis for diarrhoea.
**Figure S19:** Subgroup analysis for dyspepsia.
**Figure S20:** Subgroup analysis for eructation.
**Figure S21:** Subgroup analysis for constipation.
**Figure S22:** Subgroup analysis for decreased appetite.
**Figure S23:** Subgroup analysis for hepatic events.
**Figure S24:** Subgroup analysis for GERD.
**Figure S25:** Subgroup analysis for abdominal pain.
**Figure S26:** Subgroup analysis for pancreatitis.
**Figure S27:** Subgroup analysis for abdominal distension.
**Figure S28:** Subgroup analysis for % change from baseline in ALT at Week 26.
**Figure S29:** Subgroup analysis for % change from baseline in AST at Week 26.
**Figure S30:** Subgroup analysis for % change from baseline in pancreatic amylase at Week 26.
**Figure S31:** Subgroup analysis for % change from baseline in pancreatic lipase at Week 26.
**Figure S32:** Subgroup analysis for % change from baseline in ALP at Week 26.
**Figure S33:** Side‐splitting method for GI AEs leading to discontinuation.
**Figure S34:** Side‐splitting method for nausea.
**Figure S35:** Side‐splitting method for vomiting.
**Figure S36:** Side‐splitting method for diarrhoea.
**Figure S37:** Side‐splitting method for dyspepsia.
**Figure S38:** Side‐splitting method for eructation.
**Figure S39:** Side‐splitting method for constipation.
**Figure S40:** Side‐splitting method for decreased appetite.
**Figure S41:** Side‐splitting method for hepatic events.
**Figure S42:** Side‐splitting method for GERD.
**Figure S43:** Side‐splitting method for abdominal pain.
**Figure S44:** Side‐splitting method for pancreatitis.
**Figure S45:** Side‐splitting method for % change from baseline in ALT at Week 26.
**Figure S46:** Side‐splitting method for % change from baseline in AST at Week 26.
**Figure S47:** Side‐splitting method for % change from baseline in pancreatic amylase at Week 26.
**Figure S48:** Side‐splitting method for % change from baseline in total serum lipase at Week 26.
**Figure S49:** Side‐splitting method for % change from baseline in ALP at Week 26.
**Table S1:** Detailed search strategy for each database.
**Table S2:** Preferred Reporting Items for Systematic Reviews and Meta‐Analysis (PRISMA) checklist.

## Data Availability

The data that support the findings of this study are available from the corresponding author upon reasonable request.
