# Supplementary material for: The Gastrointestinal Safety of Orforglipron, a GLP‐1 Receptor Agonist, in Adults With or Without Type 2 Diabetes: A Network Meta‐Analysis of Randomized Controlled Trials
Source: Endocrinol Diabetes Metab. 2026 May 11;9(3):e70222. doi: 10.1002/edm2.70222 (PMC13161550; doi:10.1002/edm2.70222)

Supplementary Appendix

**The Gastrointestinal Safety of Orforglipron, a GLP-1 Receptor Agonist, in Adults with or without Type 2 Diabetes: A Network Meta-analysis of Randomized Controlled Trials.**

**Authors.**

Ahmed W. Hageen, MBBCh^1*;^ Ahmed Farid Gadelmawla, MD^2,3^; Ahmad Omar Saleh, MD^4^; Abdallfatah Abdallfatah, MBBCh^5^; Mohamed Reyad Mohamed, MD^6^; Amira Fahmy El-Nemr, MBBCh^7^; Odai Maihoub, MD^8^; Ahmed Elsekhary, MBBCh^9^,  Safir Eladawi, MBBCh^10^; Biruk Demisse Ayalew, MD^11*^; Ahmed Mansour, MD^7^; Ayoup Ahmed Radi, MBBCh^11^; Mohamed Abuelazm, MBBCh^1^; Mohamed Galal Flefel, MD, PhD^12^.

**Affiliations.**

1. Faculty of Medicine, Tanta University, Tanta, Egypt.
2. Faculty of Medicine, Menoufia University, Menoufia, Egypt.
3. Medical Research Group of Egypt (MRGE), Negida Academy, Arlington, MA, USA.
4. Faculty of medicine, The university of Jordan, Amman, Jordan.
5. Faculty of Medicine, October 6 University, Giza, Egypt.
6. Department of Medicine, University of Arizona College of Medicine - Phoenix, Phoenix, AZ, USA.
7. Faculty of Medicine, Al-Azhar University, Cairo, Egypt.
8. Department of Pathology, National Hospital, Latakia, Syria.
9. Kasr Alainy School of Medicine, Cairo University, Cairo, Egypt.
10. Faculty of Medicine, Ain Shams University, Cairo, Egypt.
11. Department of Internal Medicine, St. Paul's Hospital Millennium Medical College, Addis Ababa University School of Medicine, Addis Ababa, Ethiopia.
12. Faculty of Medicine, Minia University, Minia, Egypt.
13. Department of Internal Medicine, Faculty of Medicine, Tanta University, Tanta, Egypt.

| **APPENDIX Page** | |
| --- | --- |
| **TABLES** | |
| **Table S1.** Detailed search strategy for each database. | 5 |
| **Table S2.** Preferred Reporting Items for Systematic Reviews and Meta-Analysis (PRISMA) checklist. | 6 |
| **FIGURES** | |
| **Figure S1.** Forest plot for GI AEs leading to discontinuation. | 10 |
| **Figure S2.** Forest plot for nausea. | 10 |
| **Figure S3.** Forest plot for vomiting. | 10 |
| **Figure S4.** Forest plot for diarrhea. | 11 |
| **Figure S5**. Forest plot for eructation. | 11 |
| **Figure S6**. Forest plot for constipation. | 11 |
| **Figure S7**. Forest plot for abdominal pain. | 12 |
| **Figure S8**. Forest plot for decreased appetite, | 12 |
| **Figure S9.** Forest plot for GERD. | 12 |
| **Figure S10**. Forest plot for dyspepsia. | 13 |
| **Figure S11**. Forest plot for pancreatitis. | 13 |
| **Figure S12**. Forest plot for abdominal distension. | 13 |
| **Figure S13**. Forest plot for hepatic events. | 14 |
| **Figure S14**. Net graphs for safety outcomes: **(A)** GI AEs leading to discontinuation,**(B)** nausea, **(C)** vomiting, **(D)** diarrhea, **(E)** eructation, **(F)** constipation, **(J)** abdominal pain, **(H)** decreased appetite, **(I)** GERD, **(J)** dyspepsia, **(K)** abdominal distension, **(L)** hepatic events, **(M)** pancreatitis. | 15 |
| **Figure S15**. Subgroup analysis for GI AEs leading to discontinuation. | 16 |
| **Figure S16**. Subgroup analysis for nausea. | 17 |
| **Figure S17**. Subgroup analysis for vomiting. | 18 |

| **Figure S18**. Subgroup analysis for diarrhea. | 19 |
| --- | --- |
| **Figure S19**. Subgroup analysis for dyspepsia. | 20 |
| **Figure S20**. Subgroup analysis for eructation. | 21 |
| **Figure S21**. Subgroup analysis for constipation. | 22 |
| **Figure S22**. Subgroup analysis for decreased appetite. | 23 |
| **Figure S23**. Subgroup analysis for hepatic events. | 24 |
| **Figure S24**. Subgroup analysis for GERD. | 25 |
| **Figure S25**. Subgroup analysis for abdominal pain. | 25 |
| **Figure S26**. Subgroup analysis for pancreatitis. | 25 |
| **Figure S27**. Subgroup analysis for abdominal distension. | 26 |
| **Figure S28**. Subgroup analysis for % change from baseline in ALT at week 26. | 27 |
| **Figure S29**. Subgroup analysis for % change from baseline in AST at week 26. | 28 |
| **Figure S30**. Subgroup analysis for % change from baseline in pancreatic amylase at week 26. | 29 |
| **Figure S31**. Subgroup analysis for % change from baseline in pancreatic lipase at week 26. | 30 |
| **Figure S32**. Subgroup analysis for % change from baseline in ALP at week 26. | 31 |
| **Figure S33**. Side-splitting method for GI AEs leading to discontinuation. | 32 |
| **Figure S34**. Side-splitting method for nausea. | 33 |
| **Figure S35**. Side-splitting method for vomiting. | 34 |
| **Figure S36**. Side-splitting method for diarrhea. | 35 |
| **Figure S37**. Side-splitting method for dyspepsia. | 36 |
| **Figure S38**. Side-splitting method for eructation. | 37 |
| **Figure S39**. Side-splitting method for constipation. | 38 |
| **Figure S40**. Side-splitting method for decreased appetite. | 39 |
| **Figure S41**. Side-splitting method for hepatic events. | 40 |
| **Figure S42**. Side-splitting method for GERD. | 41 |
| **Figure S43**. Side-splitting method for abdominal pain. | 42 |
| **Figure S44**. Side-splitting method for pancreatitis. | 43 |
| **Figure S45**. Side-splitting method for % change from baseline in ALT at week 26. | 44 |
| **Figure S46**. Side-splitting method for % change from baseline in AST at week 26. | 45 |
| **Figure S47**. Side-splitting method for % change from baseline in pancreatic amylase at week 26. | 46 |
| **Figure S48**. Side-splitting method for % change from baseline in total serum lipase at week 26. | 47 |
| **Figure S49**. Side-splitting method for % change from baseline in ALP at week 26. | 48 |
| ***Abbreviations.***  GI AEs; gastrointestinal adverse events, GERD; gastroesophageal reflux disease, ALT; alanine aminotransferase, AST; aspartate aminotransferase, ALP; alkaline phosphatase, NA; not applicable. | |

**Table S1:** Detailed search strategy for each database.

| **Database** | **Search Terms** | **Search Field** | | **Results** |
| --- | --- | --- | --- | --- |
| **PubMed** | ("orforglipron" OR "LY3502970" OR "small-molecule oral GLP-1RAs" OR "small-molecule oral GLP-1 receptor agonists" OR "small GLP-1RAs" OR "oral non-peptide glucagon-like peptide-1 receptor agonist" OR "oral active nonpeptide agonist") AND ("type 2 diabetes" OR "T2DM" OR "diabetes mellitus" OR "diabetes" OR "obesity" OR "obese" OR "weight" OR "overweight") | All Fields | 141 | |
| **WOS** | ("orforglipron" OR "LY3502970" OR "small-molecule oral GLP-1RAs" OR "small-molecule oral GLP-1 receptor agonists" OR "small GLP-1RAs" OR "oral non-peptide glucagon-like peptide-1 receptor agonist" OR "oral active nonpeptide agonist") AND ("type 2 diabetes" OR "T2DM" OR "diabetes mellitus" OR "diabetes" OR "obesity" OR "obese" OR "weight" OR "overweight") | All Fields | 51 | |
| **Scopus** | ("orforglipron" OR "LY3502970" OR "small-molecule oral GLP-1RAs" OR "small-molecule oral GLP-1 receptor agonists" OR "small GLP-1RAs" OR "oral non-peptide glucagon-like peptide-1 receptor agonist" OR "oral active nonpeptide agonist") AND ("type 2 diabetes" OR "T2DM" OR "diabetes mellitus" OR "diabetes" OR "obesity" OR "obese" OR "weight" OR "overweight") | Title, Abstract, Keywords | 88 | |
| **EMBASE** | ("orforglipron" OR "LY3502970" OR "small-molecule oral GLP-1RAs" OR "small-molecule oral GLP-1 receptor agonists" OR "small GLP-1RAs" OR "oral non-peptide glucagon-like peptide-1 receptor agonist" OR "oral active nonpeptide agonist") AND ("type 2 diabetes" OR "T2DM" OR "diabetes mellitus" OR "diabetes" OR "obesity" OR "obese" OR "weight" OR "overweight") | All Fields | 131 | |

**Table S2.** Preferred Reporting Items for Systematic Reviews and Meta-Analysis (PRISMA) checklist.

| **Section and Topic** | **Item #** | **Checklist item** | **Page where item is reported** |
| --- | --- | --- | --- |
| **TITLE** | | |  |
| Title | 1 | Identify the report as a systematic review. | 1 |
| **ABSTRACT** | | |  |
| Abstract | 2 | See the PRISMA 2020 for Abstracts checklist. | 4 |
| **INTRODUCTION** | | |  |
| Rationale | 3 | Describe the rationale for the review in the context of existing knowledge. | 6 |
| Objectives | 4 | Provide an explicit statement of the objective(s) or question(s) the review addresses. | 7 |
| **METHODS** | | |  |
| Eligibility criteria | 5 | Specify the inclusion and exclusion criteria for the review and how studies were grouped for the syntheses. | 8 |
| Information sources | 6 | Specify all databases, registers, websites, organisations, reference lists and other sources searched or consulted to identify studies. Specify the date when each source was last searched or consulted. | 7-8 |
| Search strategy | 7 | Present the full search strategies for all databases, registers and websites, including any filters and limits used. | 7 |
| Selection process | 8 | Specify the methods used to decide whether a study met the inclusion criteria of the review, including how many reviewers screened each record and each report retrieved, whether they worked independently, and if applicable, details of automation tools used in the process. | 8 |
| Data collection process | 9 | Specify the methods used to collect data from reports, including how many reviewers collected data from each report, whether they worked independently, any processes for obtaining or confirming data from study investigators, and if applicable, details of automation tools used in the process. | 7-8 |
| Data items | 10a | List and define all outcomes for which data were sought. Specify whether all results that were  compatible with each outcome domain in each study were sought (e.g. for all measures, time points, analyses), and if not, the methods used to decide which results to collect. | 8 |

| **Section and Topic** | **Item #** | **Checklist item** | **Page where item is**  **reported** |
| --- | --- | --- | --- |
|  | 10b | List and define all other variables for which data were sought (e.g. participant and intervention characteristics, funding sources). Describe any assumptions made about any missing or unclear information. | 8 |
| Study risk of bias assessment | 11 | Specify the methods used to assess risk of bias in the included studies, including details of the tool(s) used, how many reviewers assessed each study and whether they worked independently, and if applicable, details of automation tools used in the process. | 9 |
| Effect  measures | 12 | Specify for each outcome the effect measure(s) (e.g. risk ratio, mean difference) used in the synthesis or presentation of results. | 9 |
| Synthesis methods | 13a | Describe the processes used to decide which studies were eligible for each synthesis (e.g. tabulating the study intervention characteristics and comparing against the planned groups for each synthesis (item #5)). | 9 |
|  | 13b | Describe any methods required to prepare the data for presentation or synthesis, such as handling of missing summary statistics, or data conversions. | 9 |
|  | 13c | Describe any methods used to tabulate or visually display results of individual studies and syntheses. | 9 |
|  | 13d | Describe any methods used to synthesize results and provide a rationale for the choice(s). If  meta-analysis was performed, describe the model(s), method(s) to identify the presence and extent of statistical heterogeneity, and software package(s) used. | 9 |
|  | 13e | Describe any methods used to explore possible causes of heterogeneity among study results (e.g. subgroup analysis, meta-regression). | 9 |
|  | 13f | Describe any sensitivity analyses conducted to assess robustness of the synthesized results. | NA |
| Reporting bias assessment | 14 | Describe any methods used to assess risk of bias due to missing results in a synthesis (arising from reporting biases). | 8-9 |
| Certainty assessment | 15 | Describe any methods used to assess certainty (or confidence) in the body of evidence for an outcome. | NA |
| **RESULTS** | | |  |

| **Section and Topic** | **Item #** | **Checklist item** | **Page where item is**  **reported** |
| --- | --- | --- | --- |
| Study selection | 16a | Describe the results of the search and selection process, from the number of records identified in the search to the number of studies included in the review, ideally using a flow diagram. | 8-9 |
|  | 16b | Cite studies that might appear to meet the inclusion criteria, but which were excluded, and explain why they were excluded. | 8-9 |
| Study  characteristics | 17 | Cite each included study and present its characteristics. | 10 |
| Risk of bias in studies | 18 | Present assessments of risk of bias for each included study. | 10 |
| Results of individual studies | 19 | For all outcomes, present, for each study: (a) summary statistics for each group (where appropriate) and (b) an effect estimate and its precision (e.g. confidence/credible interval), ideally using structured tables or plots. | 10 |
| Results of syntheses | 20a | For each synthesis, briefly summarise the characteristics and risk of bias among contributing studies. | 10-11 |
|  | 20b | Present results of all statistical syntheses conducted. If meta-analysis was done, present for each the summary estimate and its precision (e.g. confidence/credible interval) and measures of statistical heterogeneity. If comparing groups, describe the direction of the effect. | 11-16 |
|  | 20c | Present results of all investigations of possible causes of heterogeneity among study results. | 16 |
|  | 20d | Present results of all sensitivity analyses conducted to assess the robustness of the synthesized results. | NA |
| Reporting biases | 21 | Present assessments of risk of bias due to missing results (arising from reporting biases) for each synthesis assessed. | 8&10-11 |
| Certainty of evidence | 22 | Present assessments of certainty (or confidence) in the body of evidence for each outcome assessed. | NA |
| **DISCUSSION** | | |  |
| Discussion | 23a | Provide a general interpretation of the results in the context of other evidence. | 16 |
|  | 23b | Discuss any limitations of the evidence included in the review. | 20 |

| **Section and Topic** | **Item #** | **Checklist item** | **Page where item is**  **reported** |
| --- | --- | --- | --- |
|  | 23c | Discuss any limitations of the review processes used. | 20 |
|  | 23d | Discuss implications of the results for practice, policy, and future research. | 20 |
| **OTHER INFORMATION** | | |  |
| Registration and protocol | 24a | Provide registration information for the review, including register name and registration number, or state that the review was not registered. | 7 |
|  | 24b | Indicate where the review protocol can be accessed, or state that a protocol was not prepared. | 7 |
|  | 24c | Describe and explain any amendments to information provided at registration or in the protocol. | 7 |
| Support | 25 | Describe sources of financial or non-financial support for the review, and the role of the funders or sponsors in the review. | 21 |
| Competing interests | 26 | Declare any competing interests of review authors. | 21 |
| Availability of data, code and other materials | 27 | Report which of the following are publicly available and where they can be found: template data collection forms; data extracted from included studies; data used for all analyses; analytic code; any other materials used in the review. | 22 |

**Reference:** Page MJ, McKenzie JE, Bossuyt PM, Boutron I, Hoffmann TC, Mulrow CD, et al. The PRISMA 2020 statement: an updated guideline for reporting systematic reviews. BMJ 2021;372:n71. doi: 10.1136/bmj.n7

**Figure S1**. Forest plot for GI AEs leading to discontinuation.


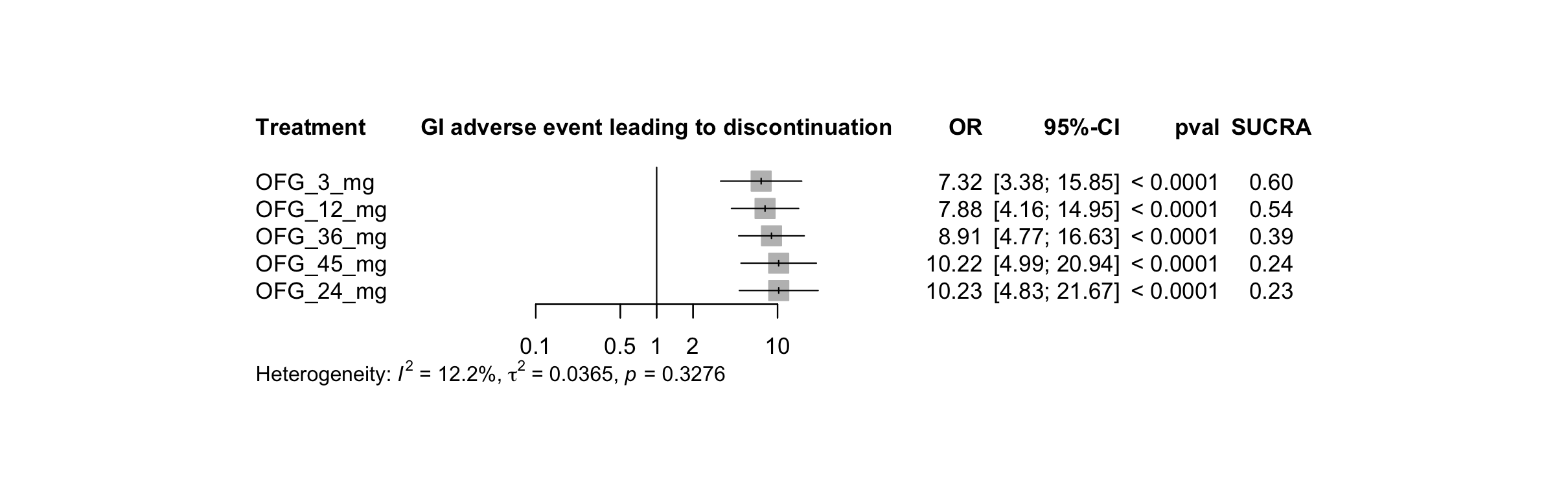


**Figure S2.** Forest plot for nausea.


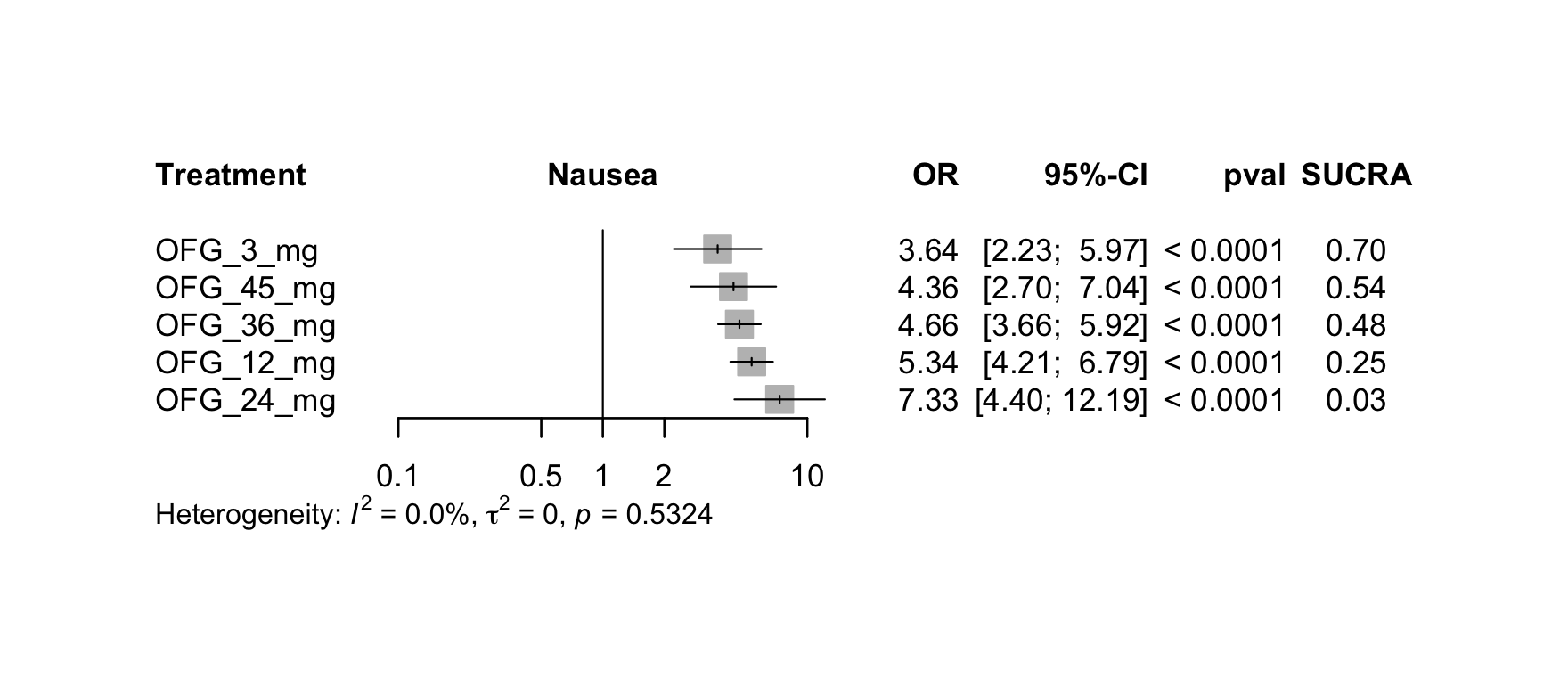


**Figure S3.** Forest plot for vomiting.


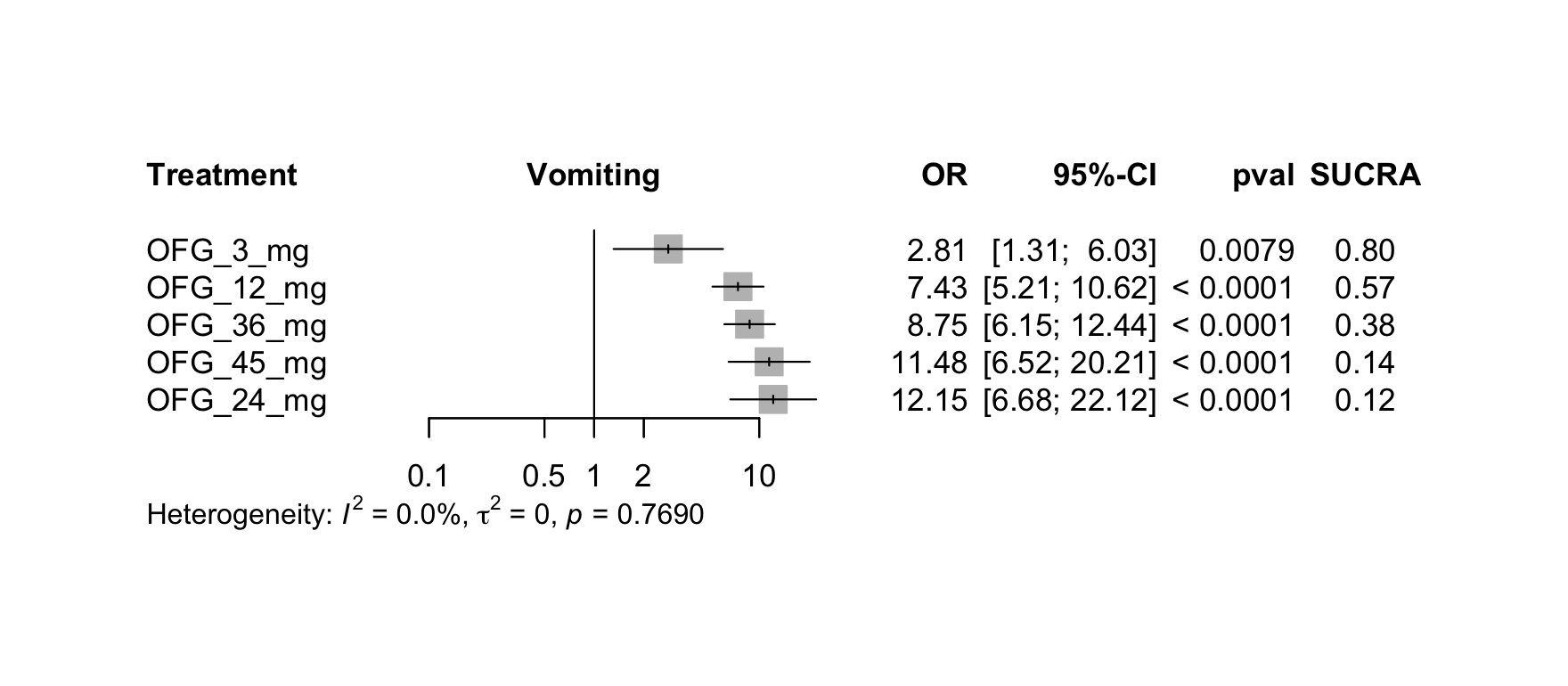


**Figure S4.** Forest plot for diarrhea.


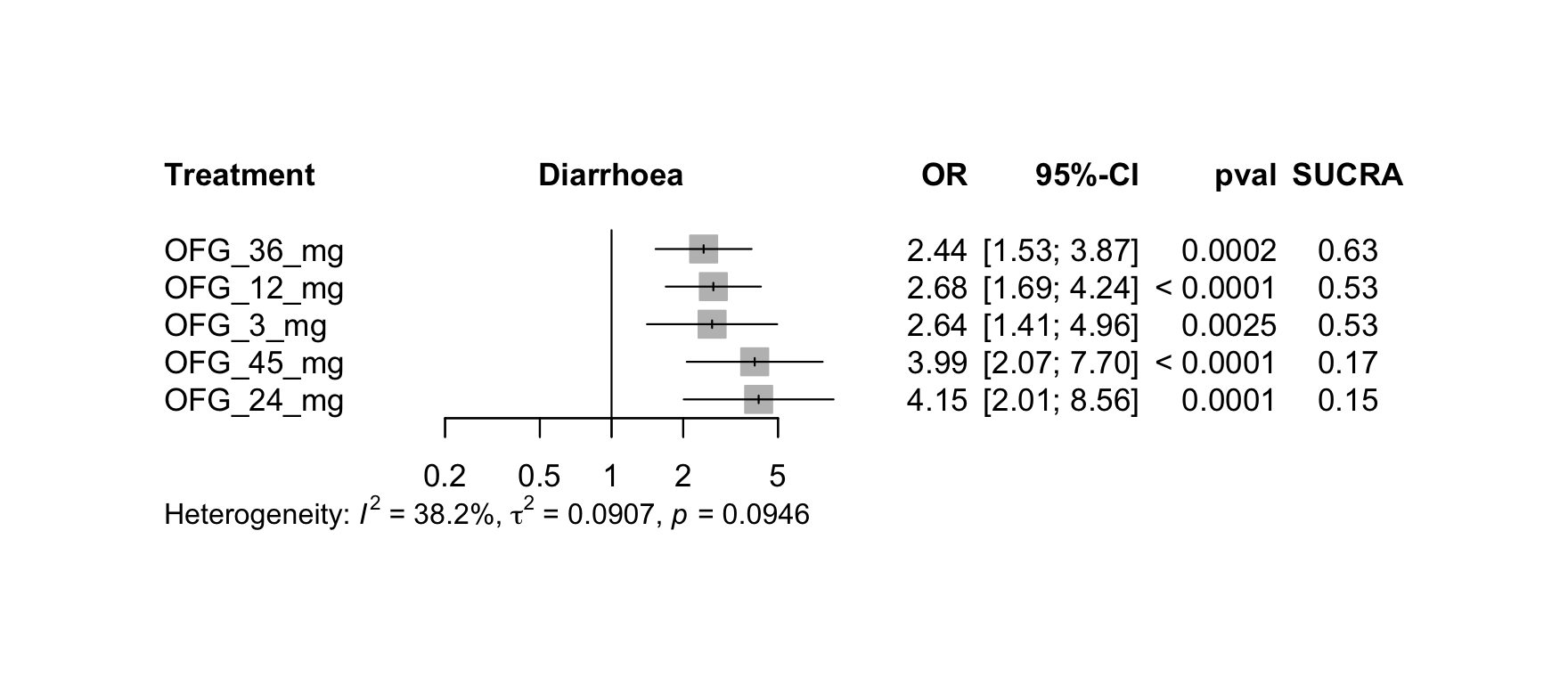


**Figure S5.** Forest plot for eructation.


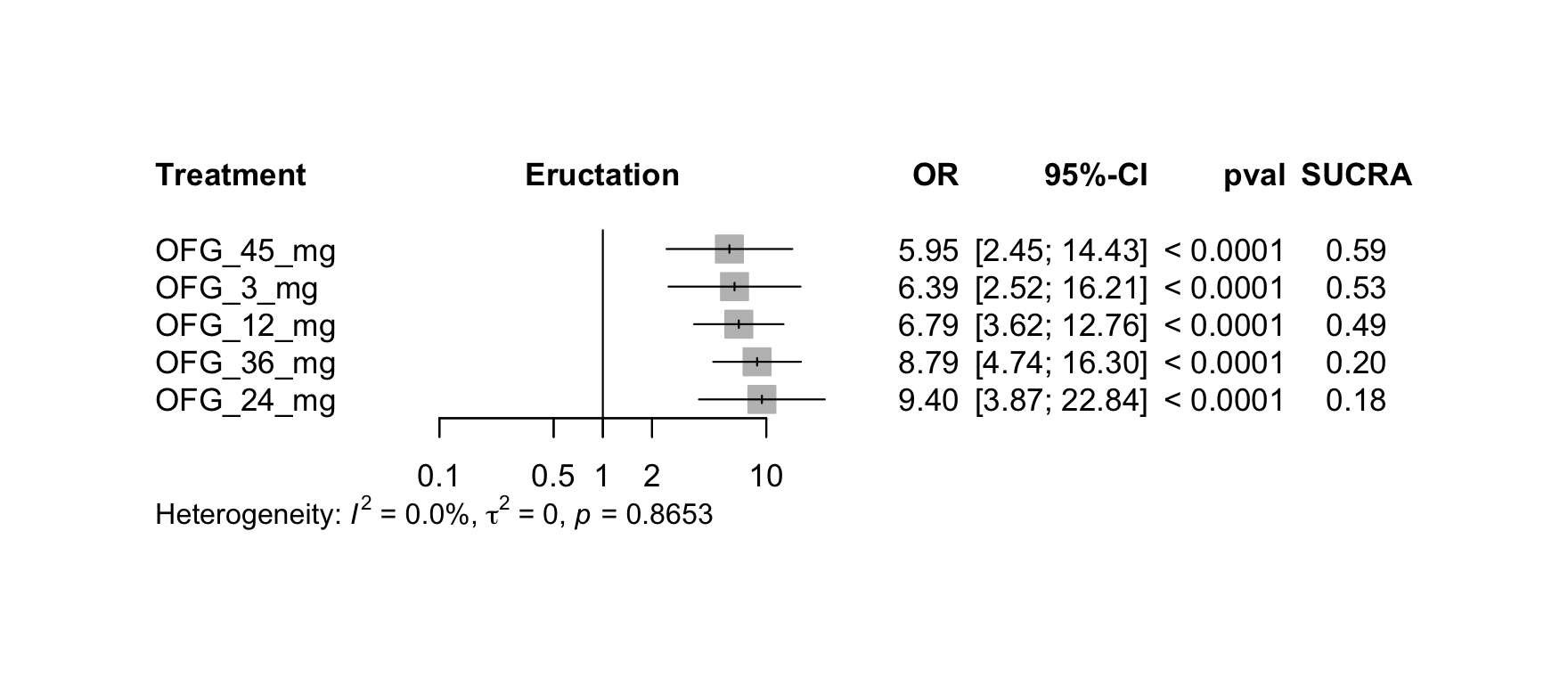


**Figure S6.** Forest plot for constipation.


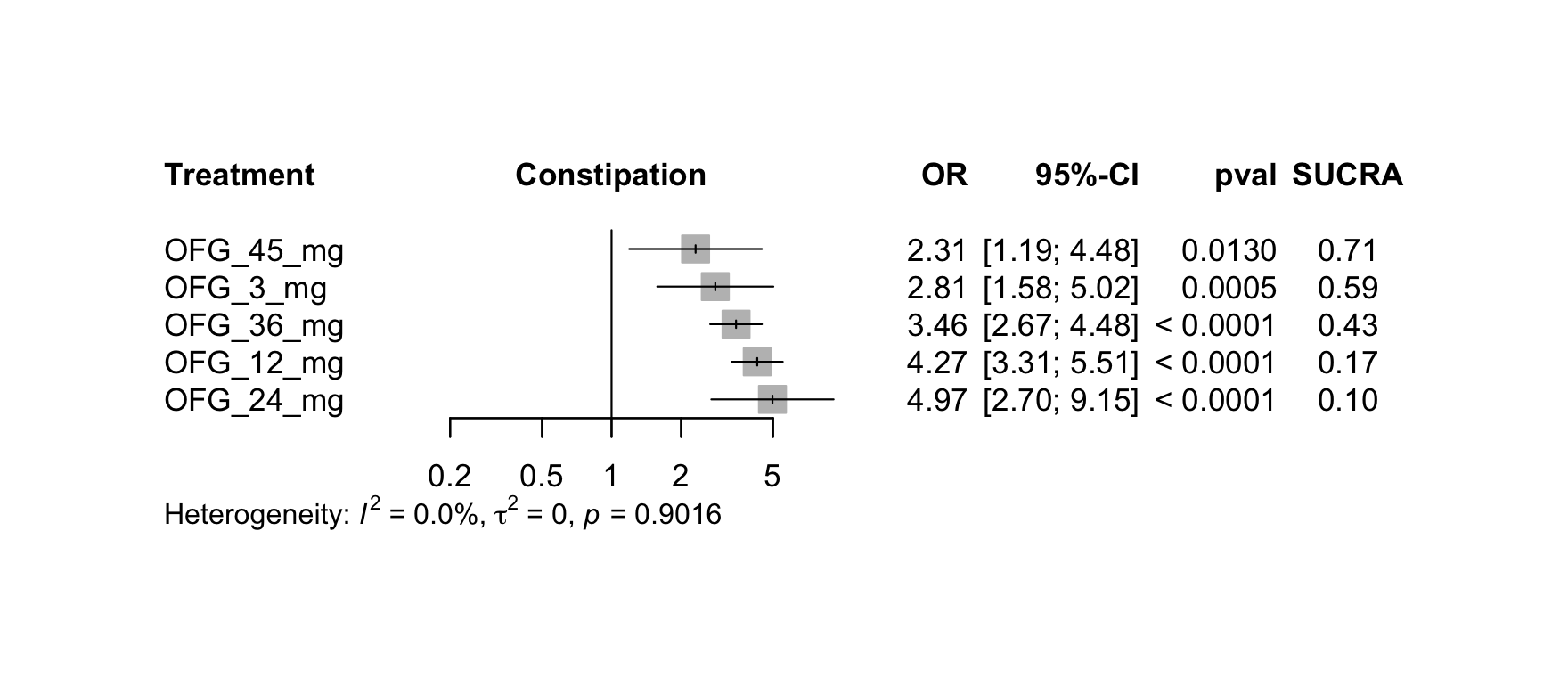


**Figure S7.** Forest plot for abdominal pain.


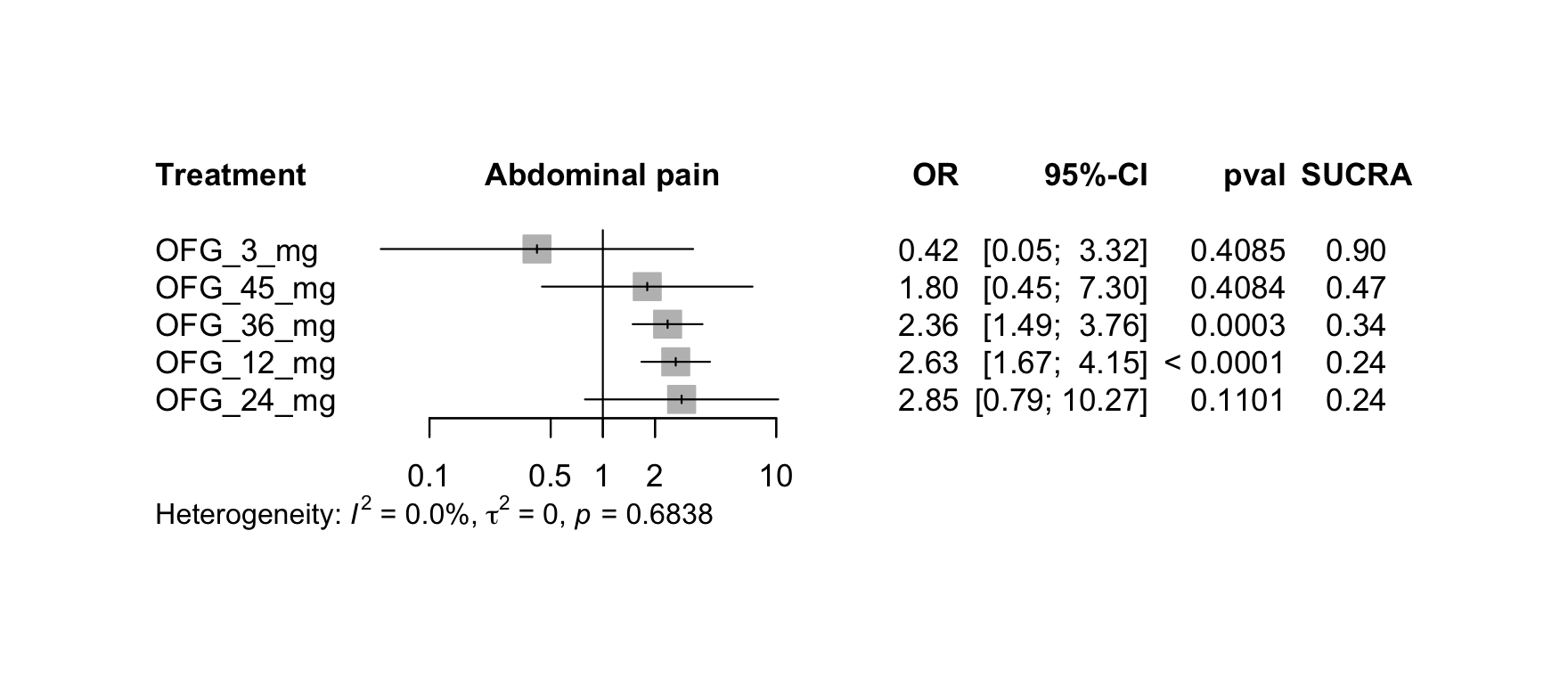


**Figure S8.** Forest plot for decreased appetite.


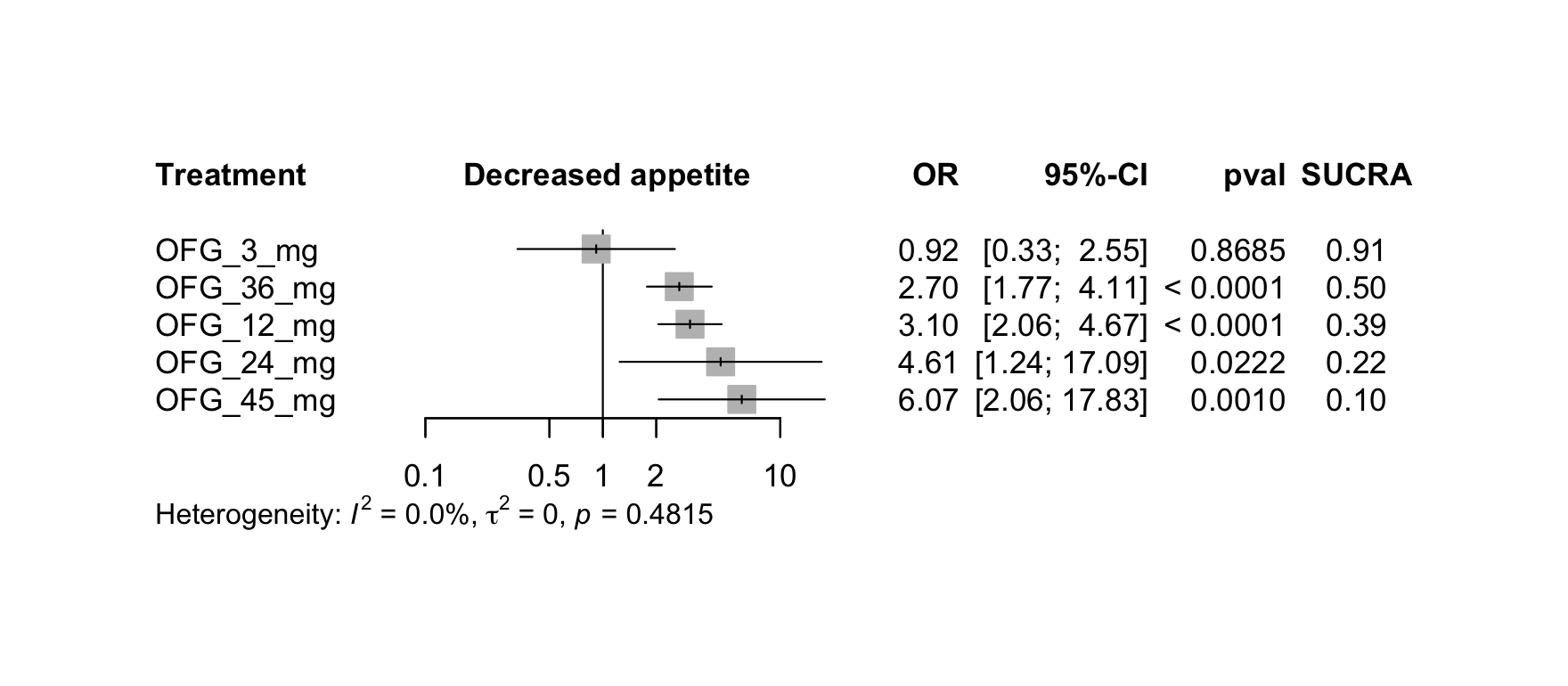


**Figure S9.** Forest plot for GERD.


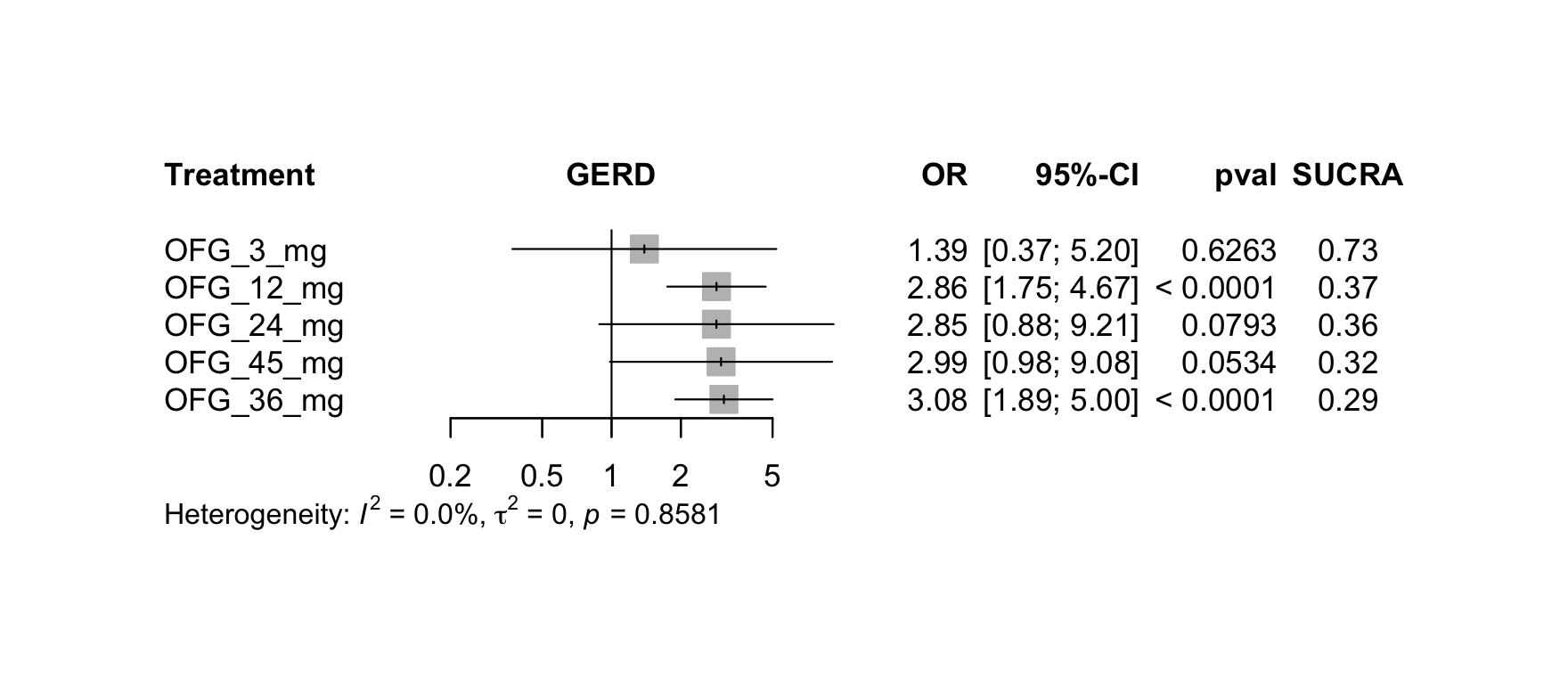


**Figure S10.** Forest plot for dyspepsia.


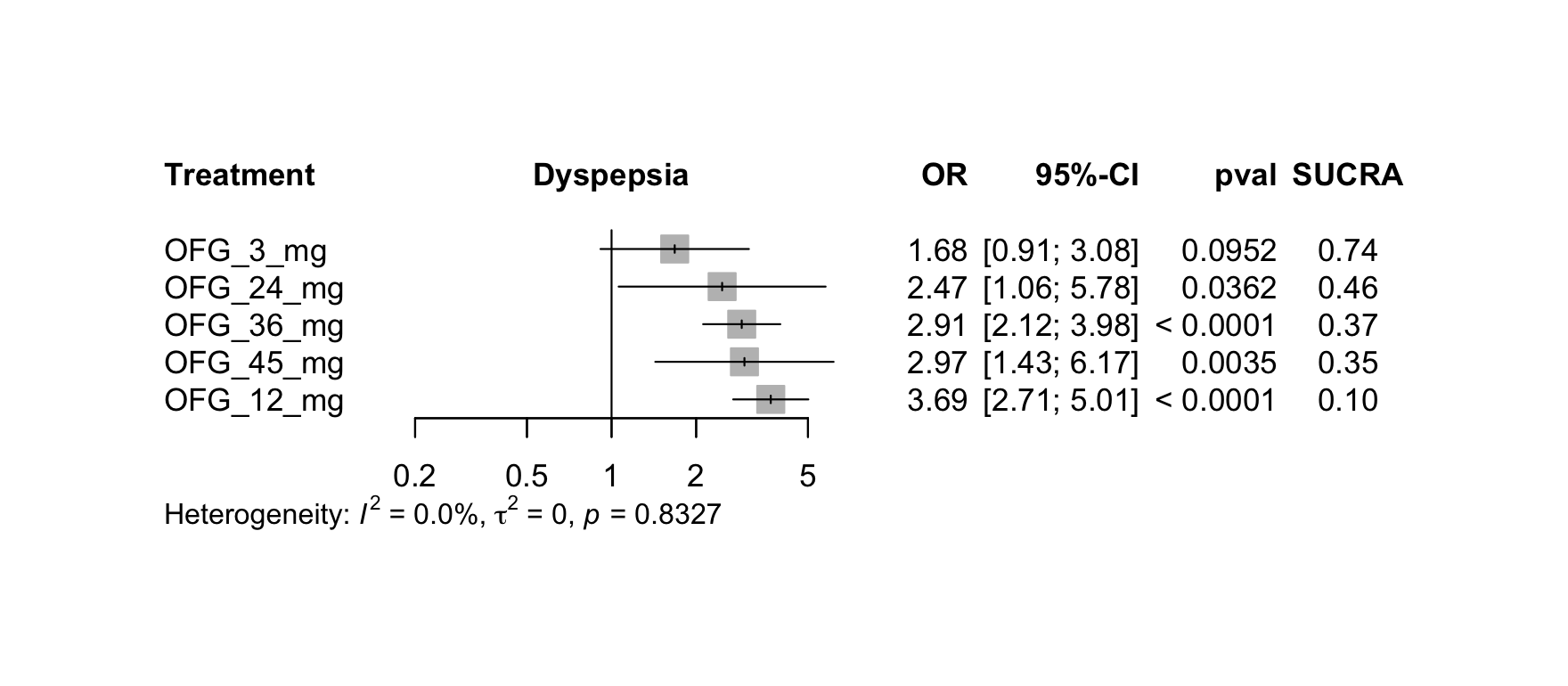


**Figure S11.** Forest plot for pancreatitis.


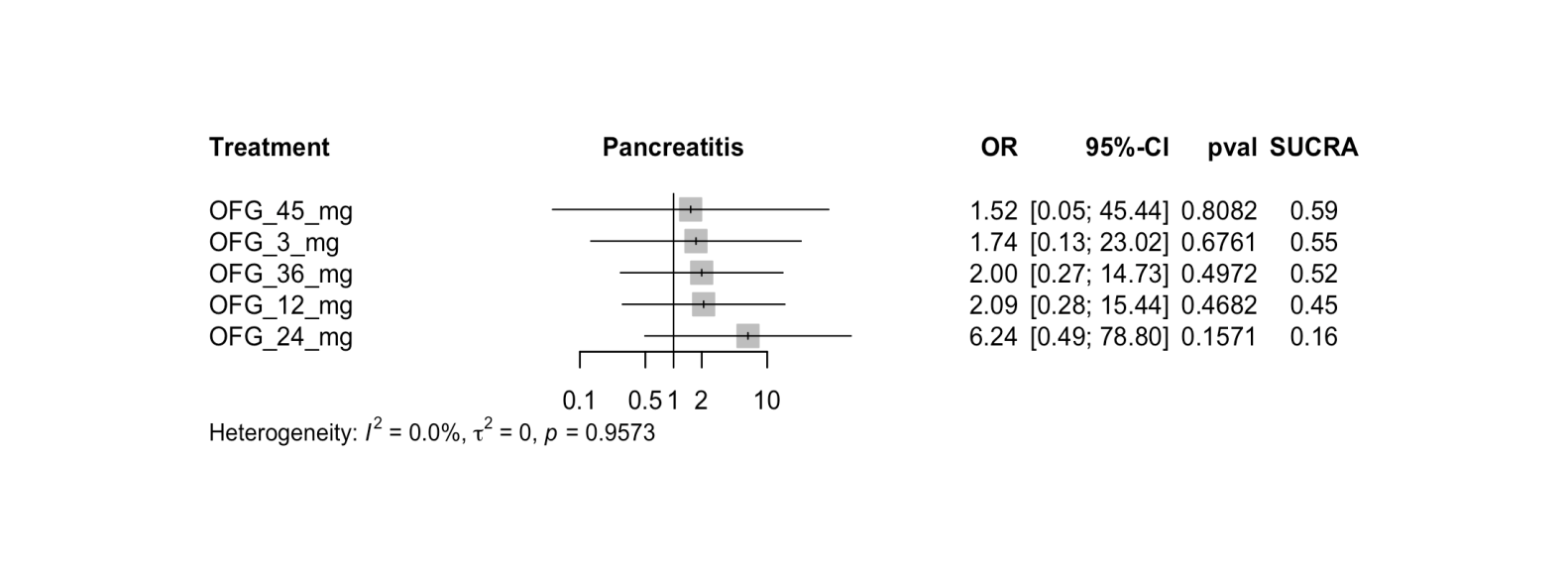
**Figure S12.** Forest plot for abdominal distension.


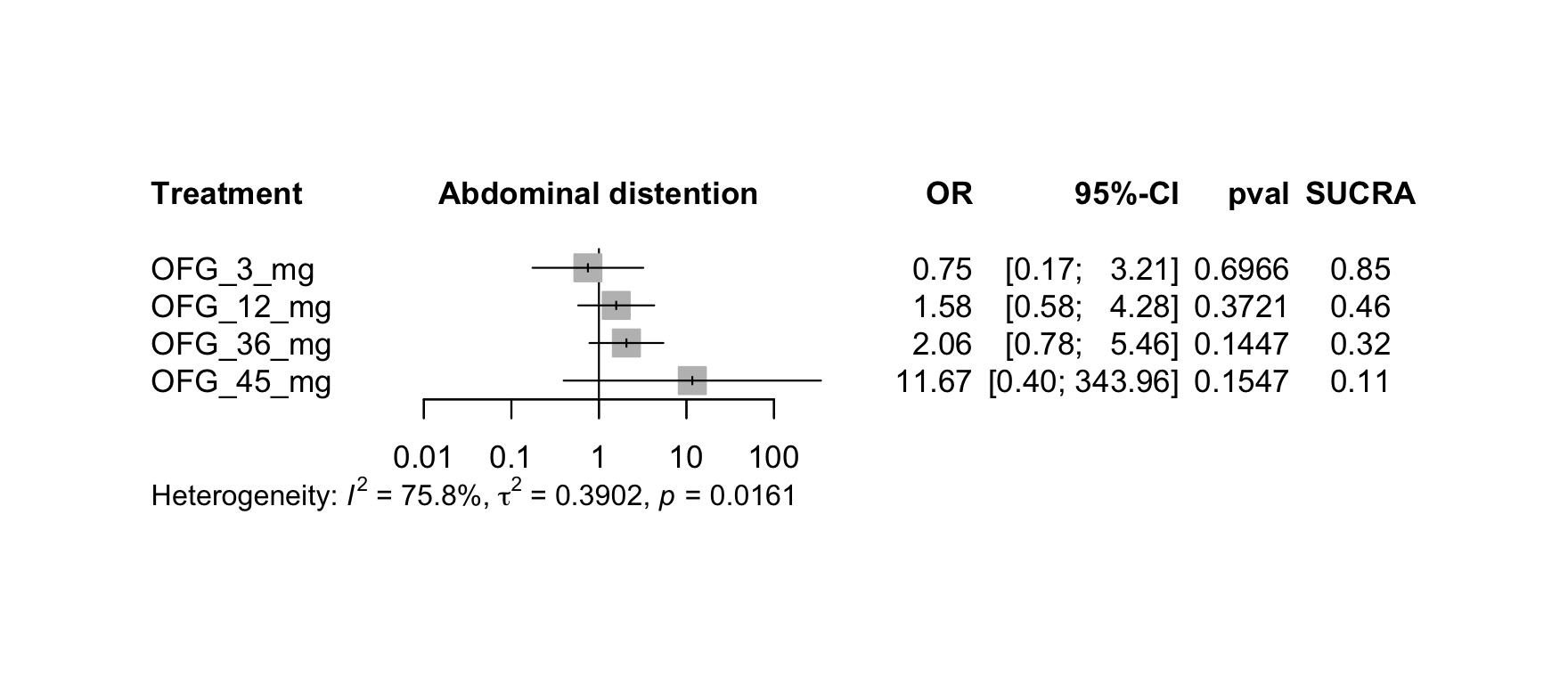


**Figure S13.** Forest plot for hepatic events.


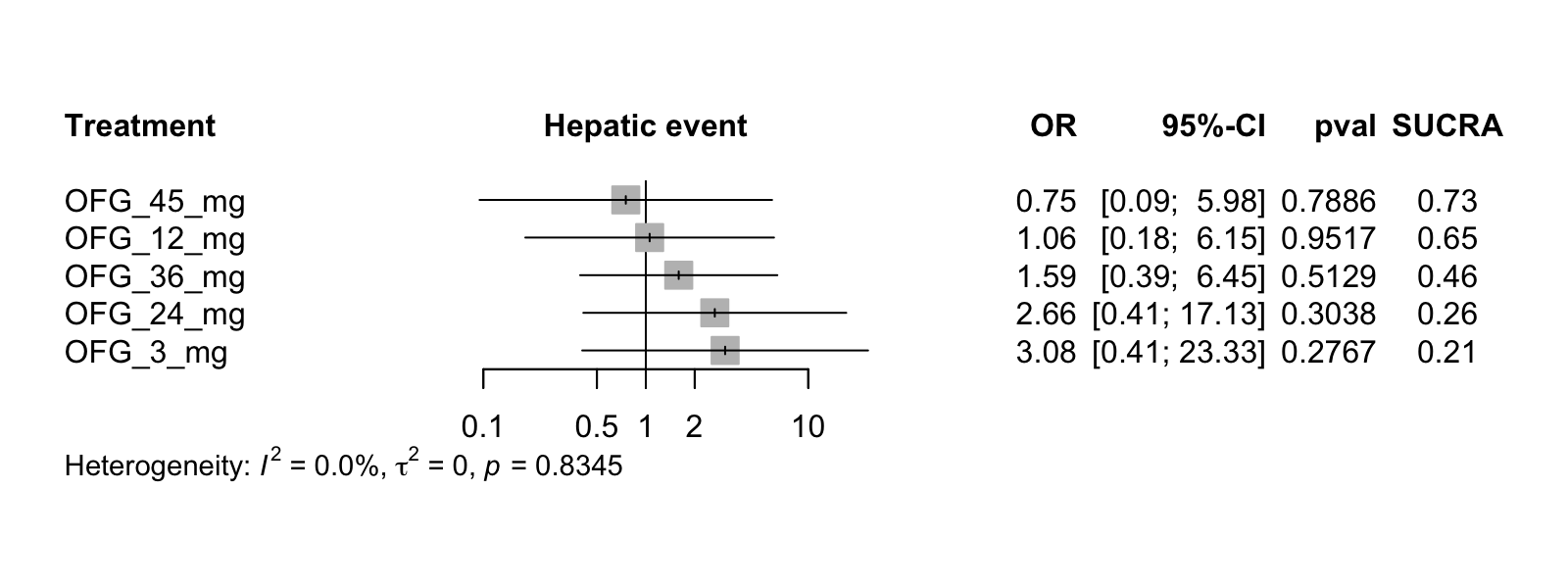


**Figure S14.** Net graphs for safety outcomes: **(A)** GI AEs leading to discontinuation, **(B)** nausea, **(C)** vomiting,**(D)** diarrhea, **(E)** eructation, **(F)** constipation, **(J)** abdominal pain, **(H)** decreased appetite, **(I)** GERD, **(J)** dyspepsia, **(K)** abdominal distension, **(L)** hepatic events, **(M)** pancreatitis.


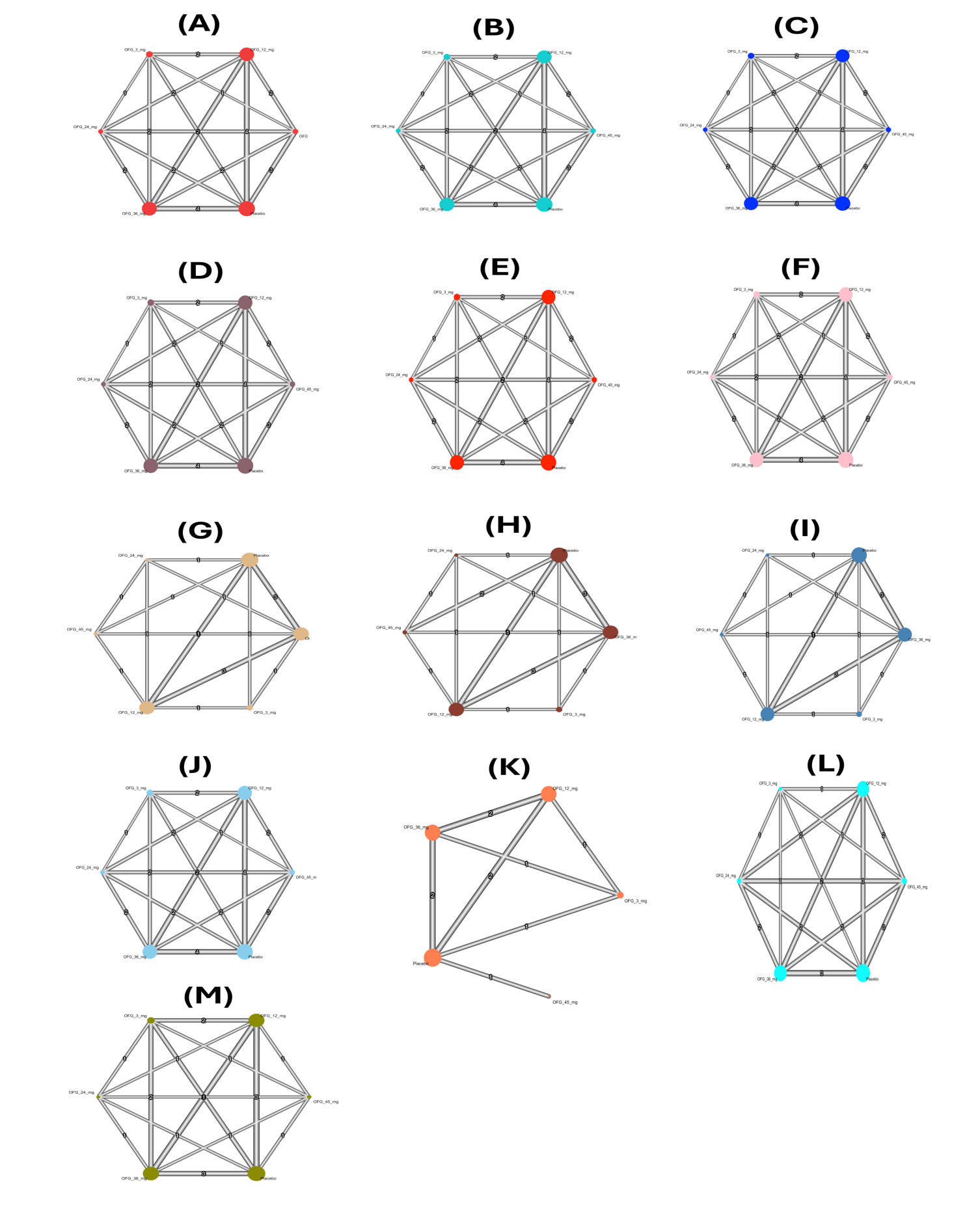


**Figure S15.** Subgroup analysis for GI AEs leading to discontinuation.


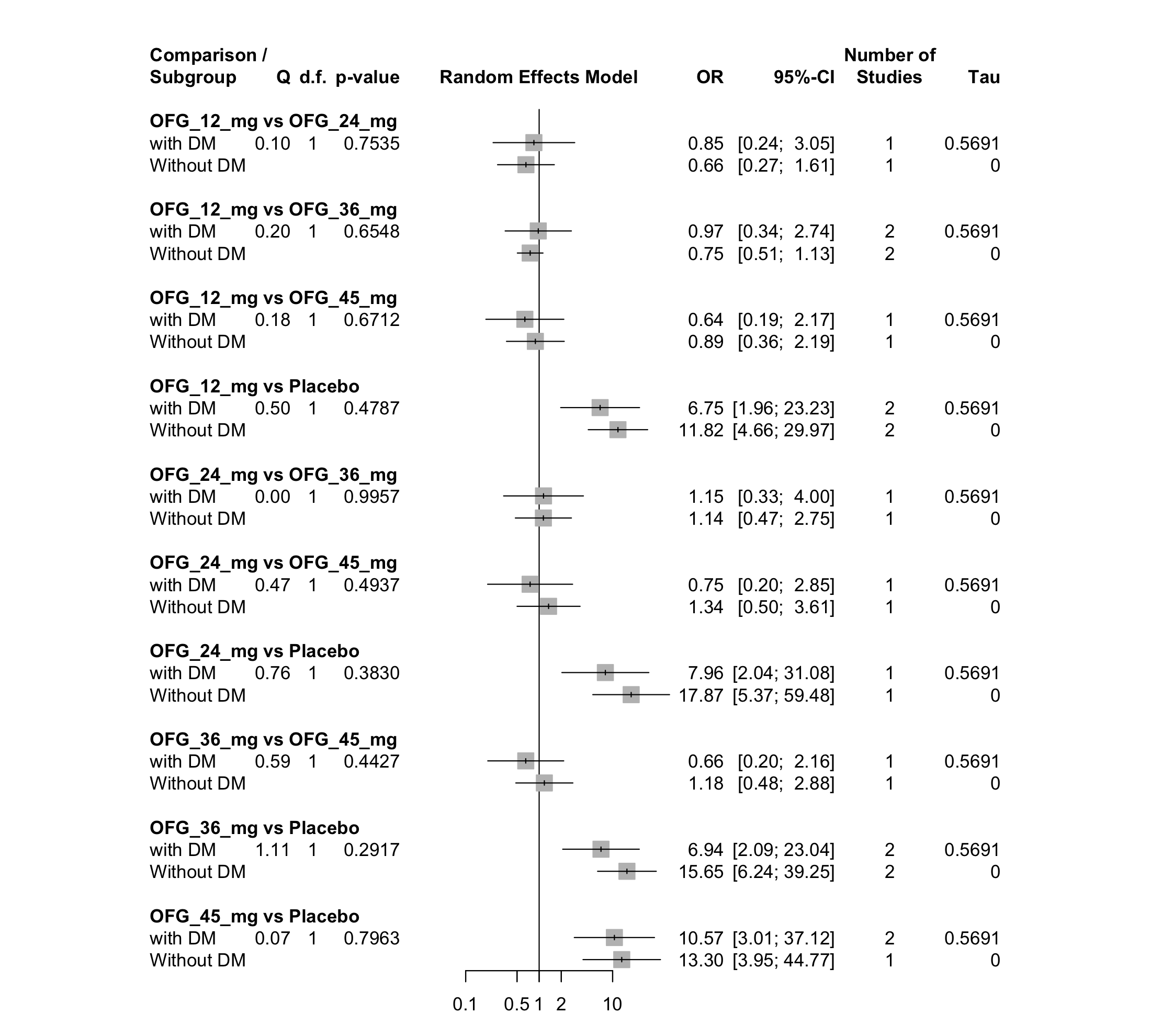


**Figure S16.** Subgroup analysis for nausea.


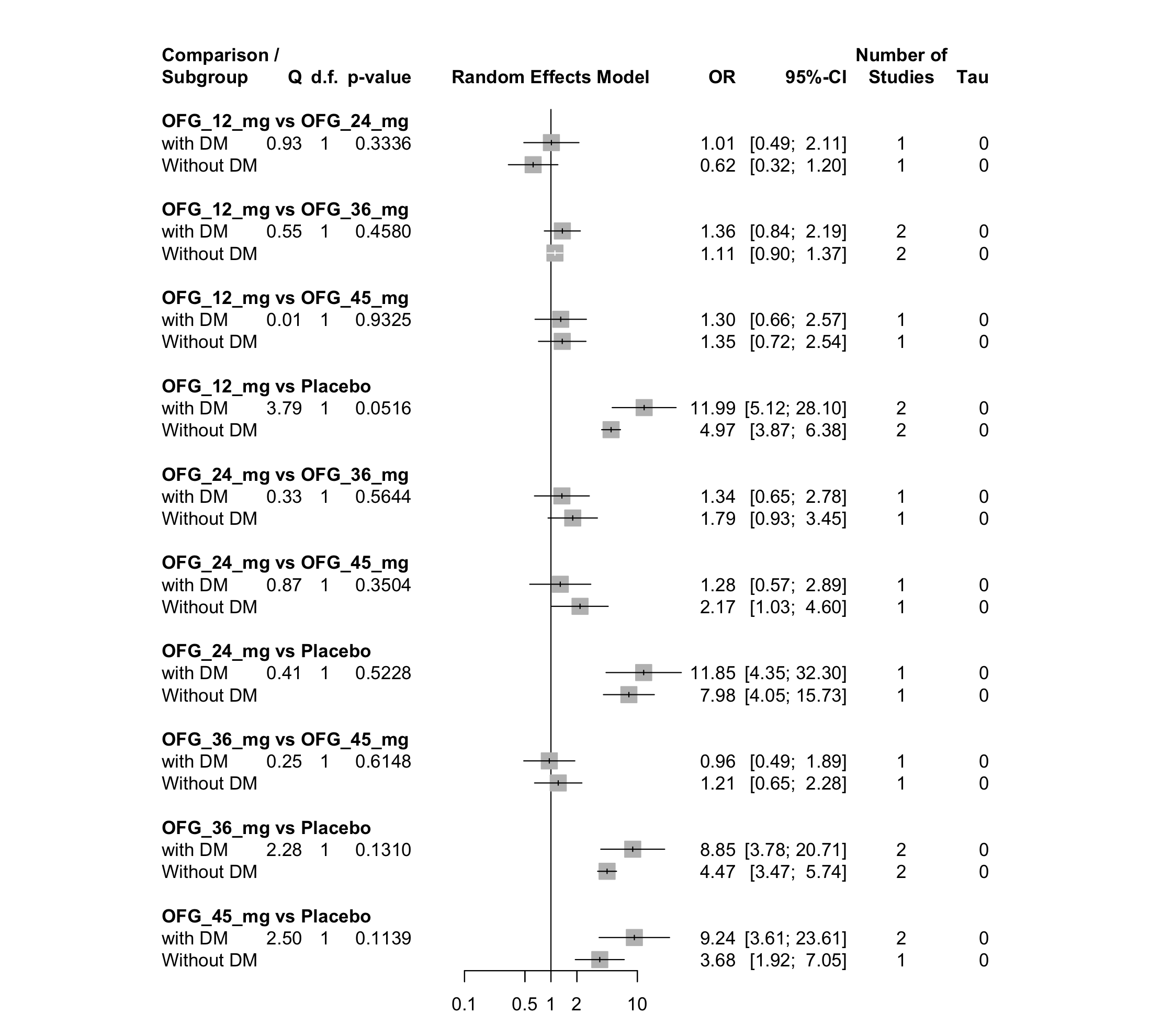


**Figure S17.** Subgroup analysis for vomiting.


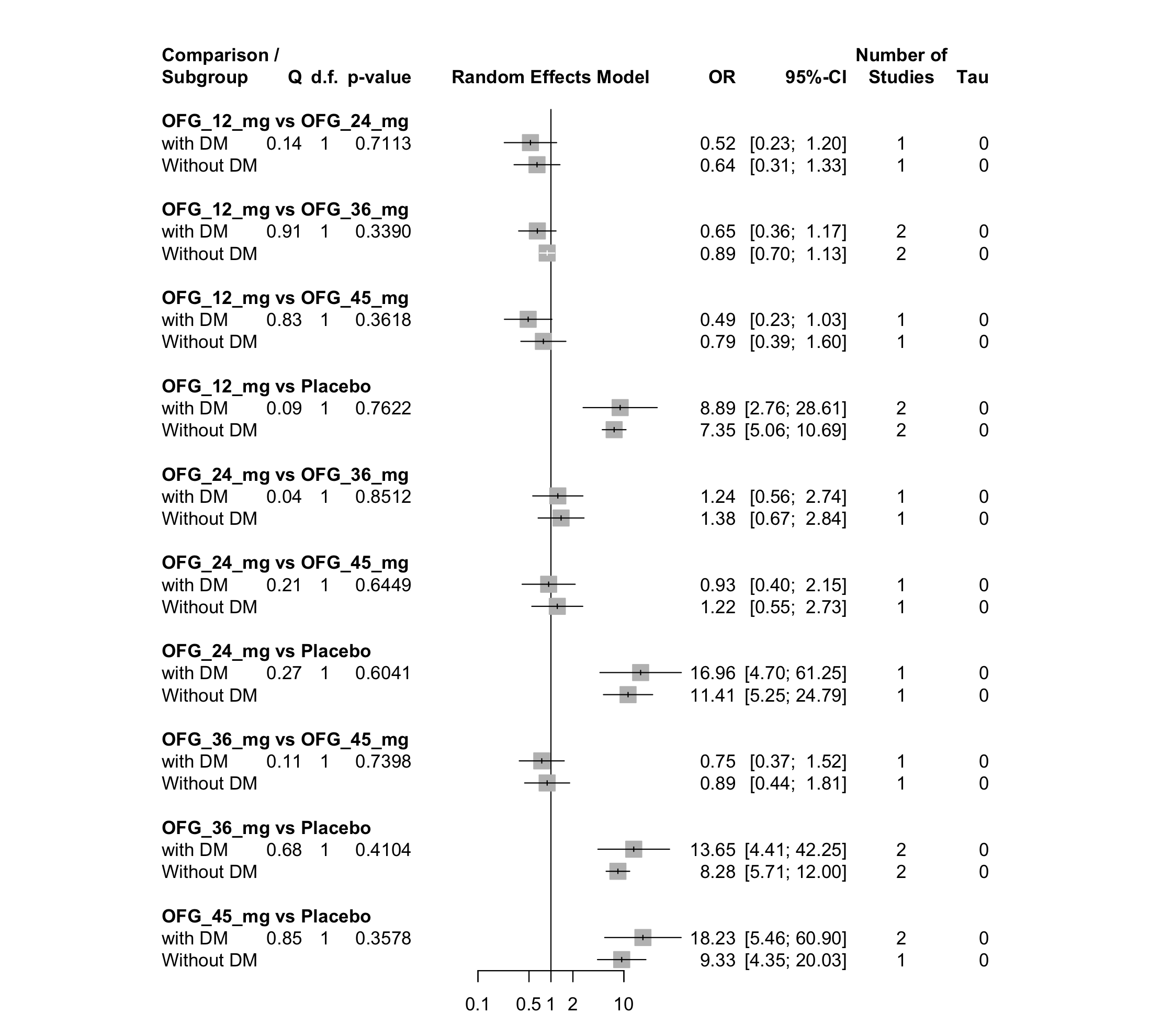


**Figure S18.** Subgroup analysis for diarrhea.


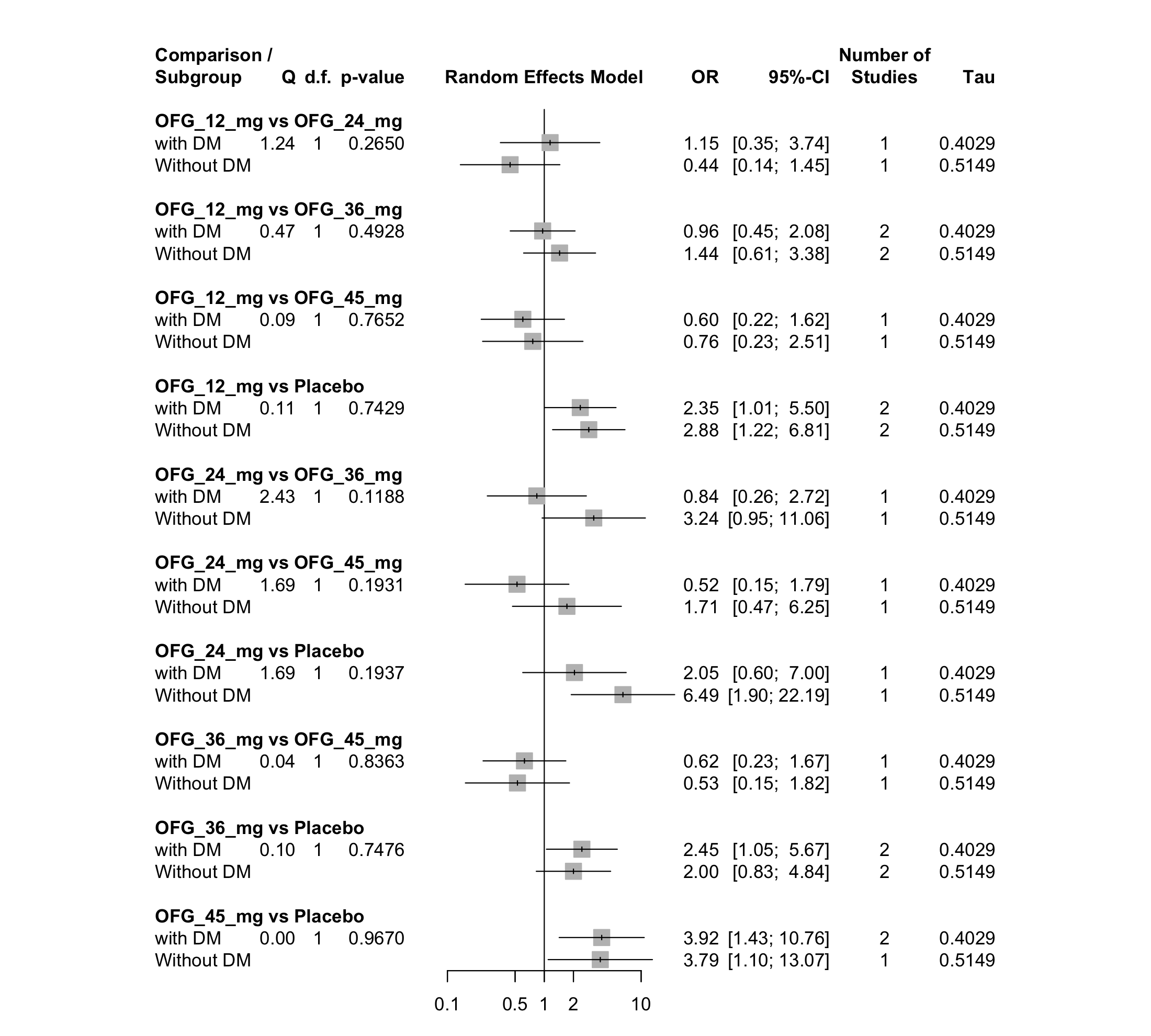


**Figure S19.** Subgroup analysis for dyspepsia.


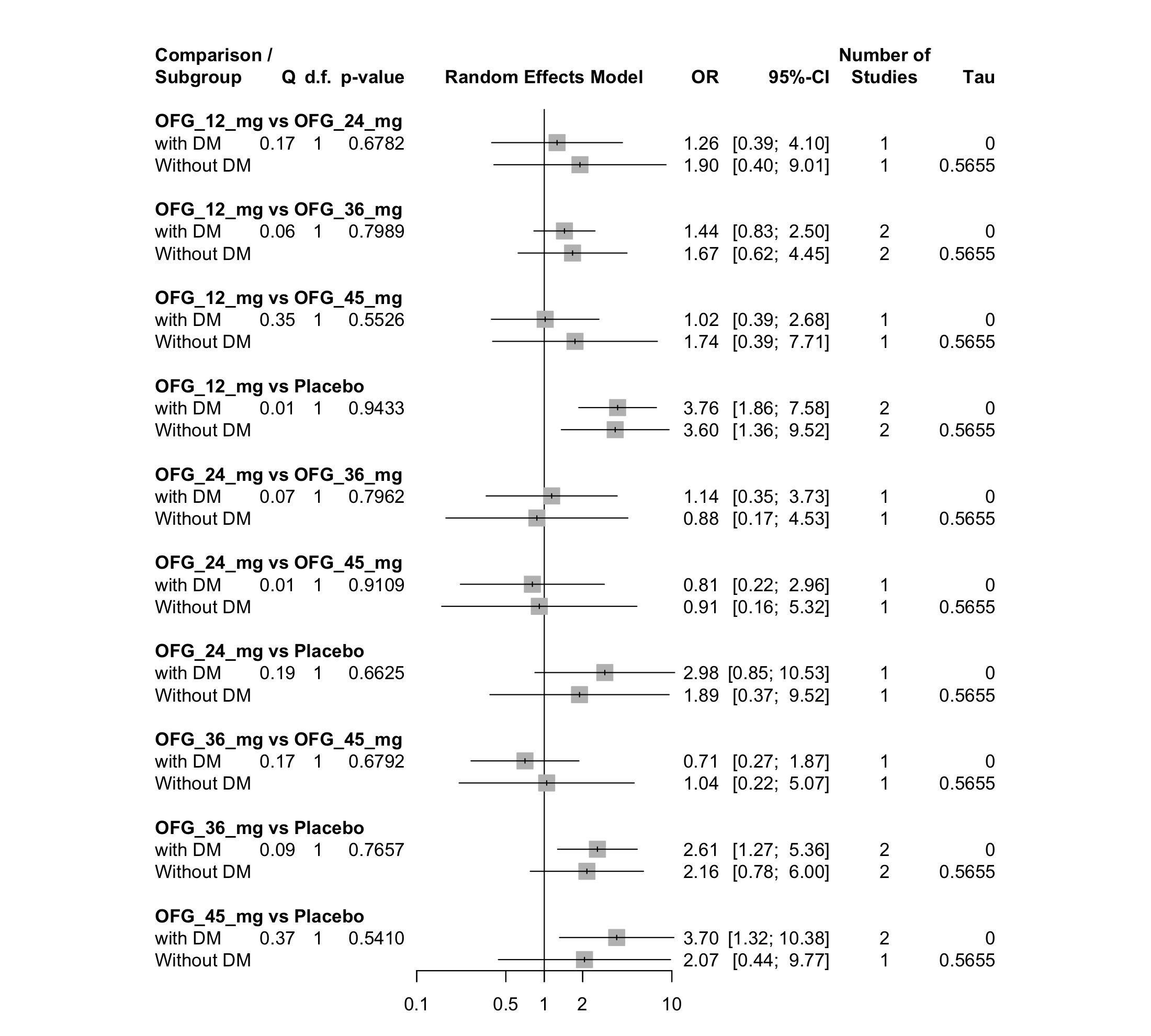


**Figure S20.** Subgroup analysis for eructation.


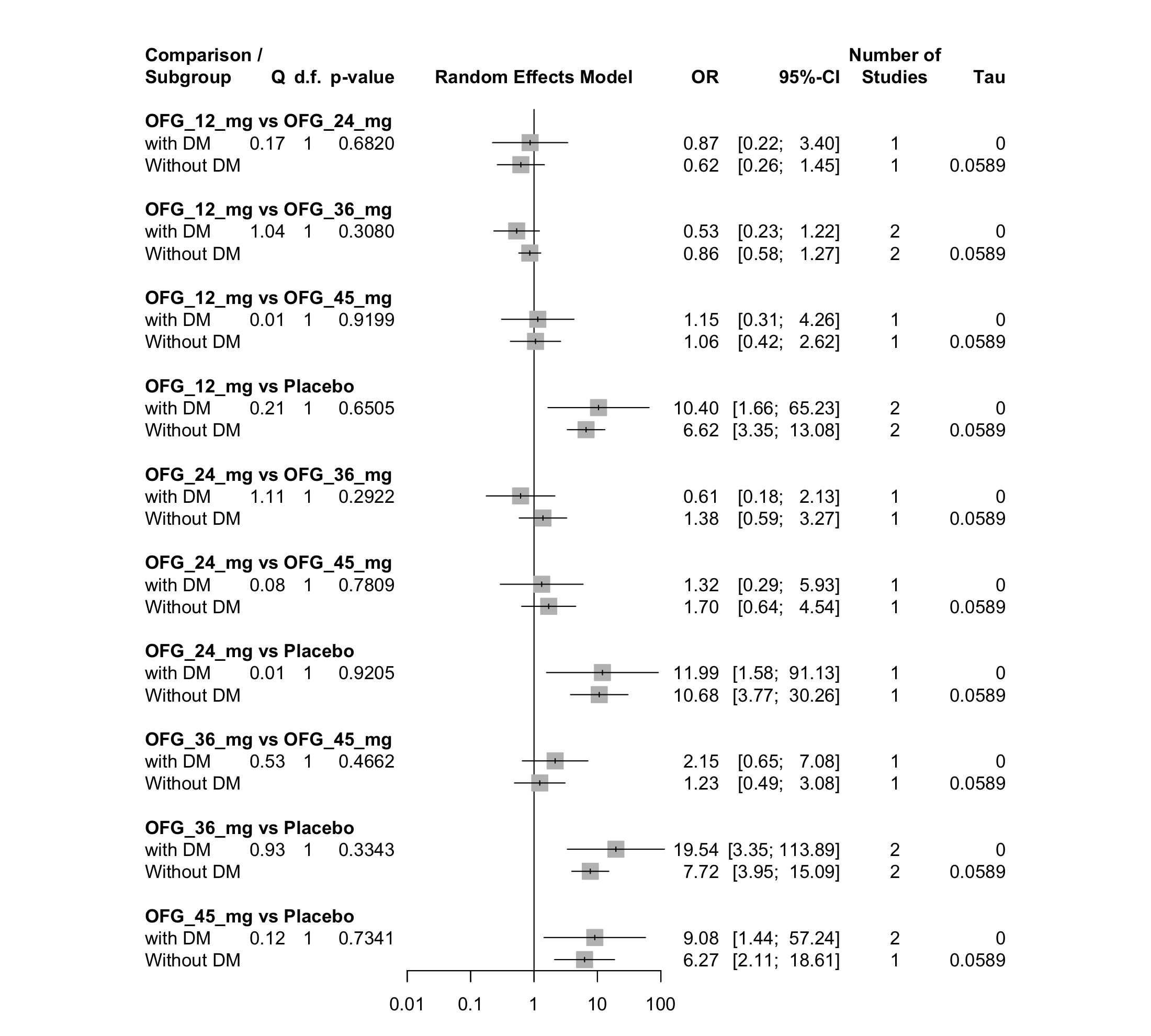


**Figure S21.** Subgroup analysis for constipation.


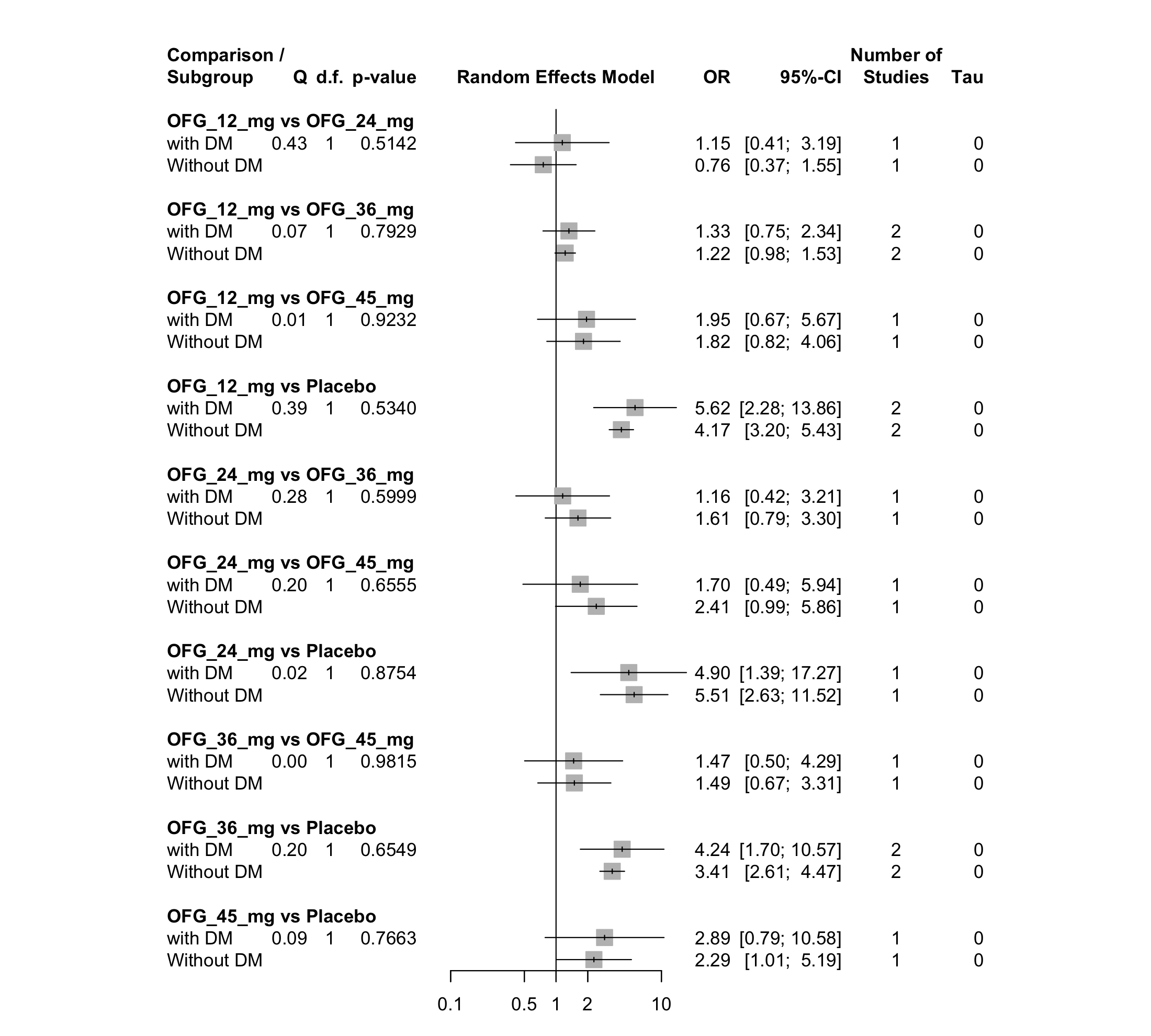


**Figure S22.** Subgroup analysis for decreased appetite.


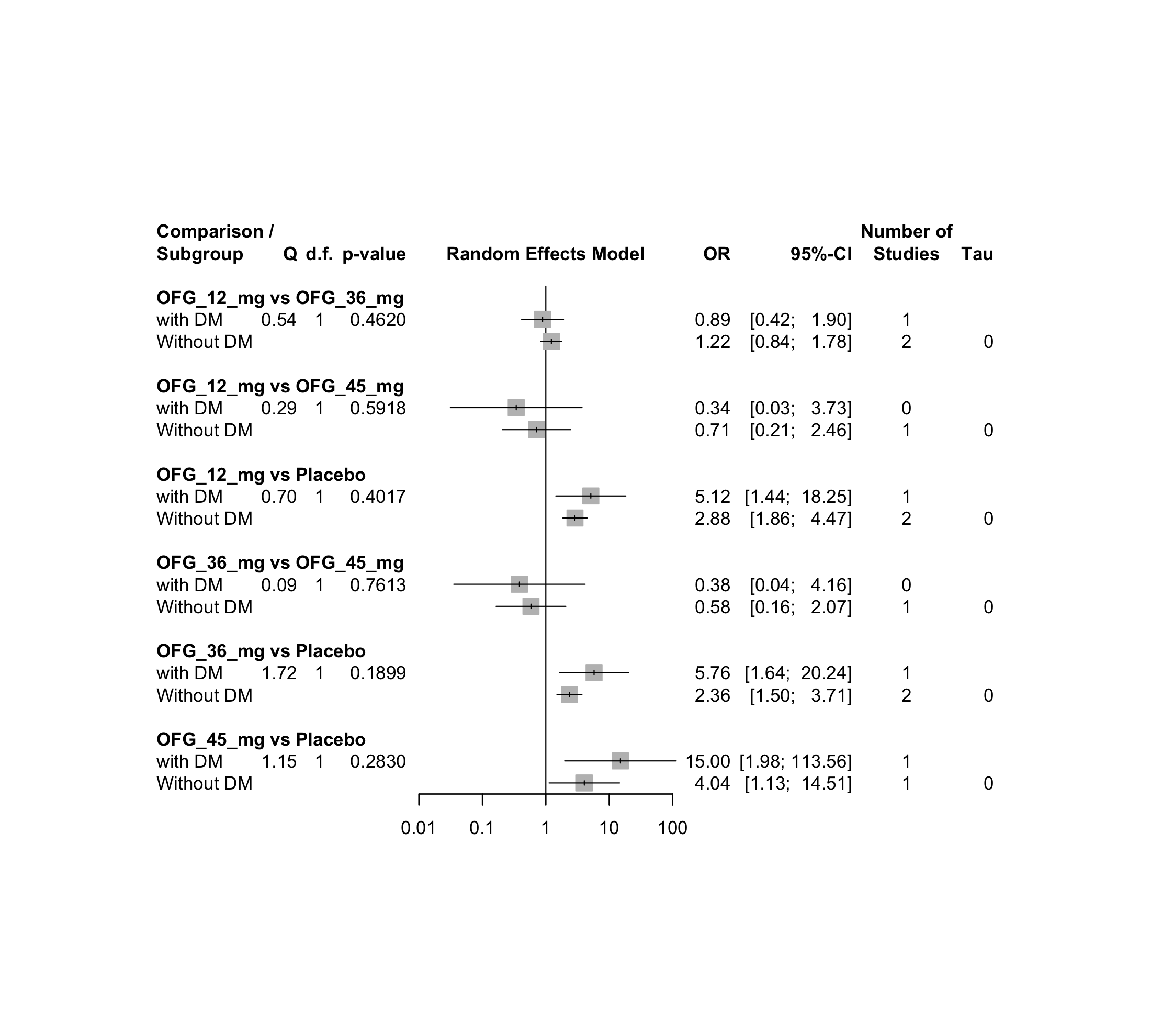


**Figure S23.** Subgroup analysis for hepatic events.


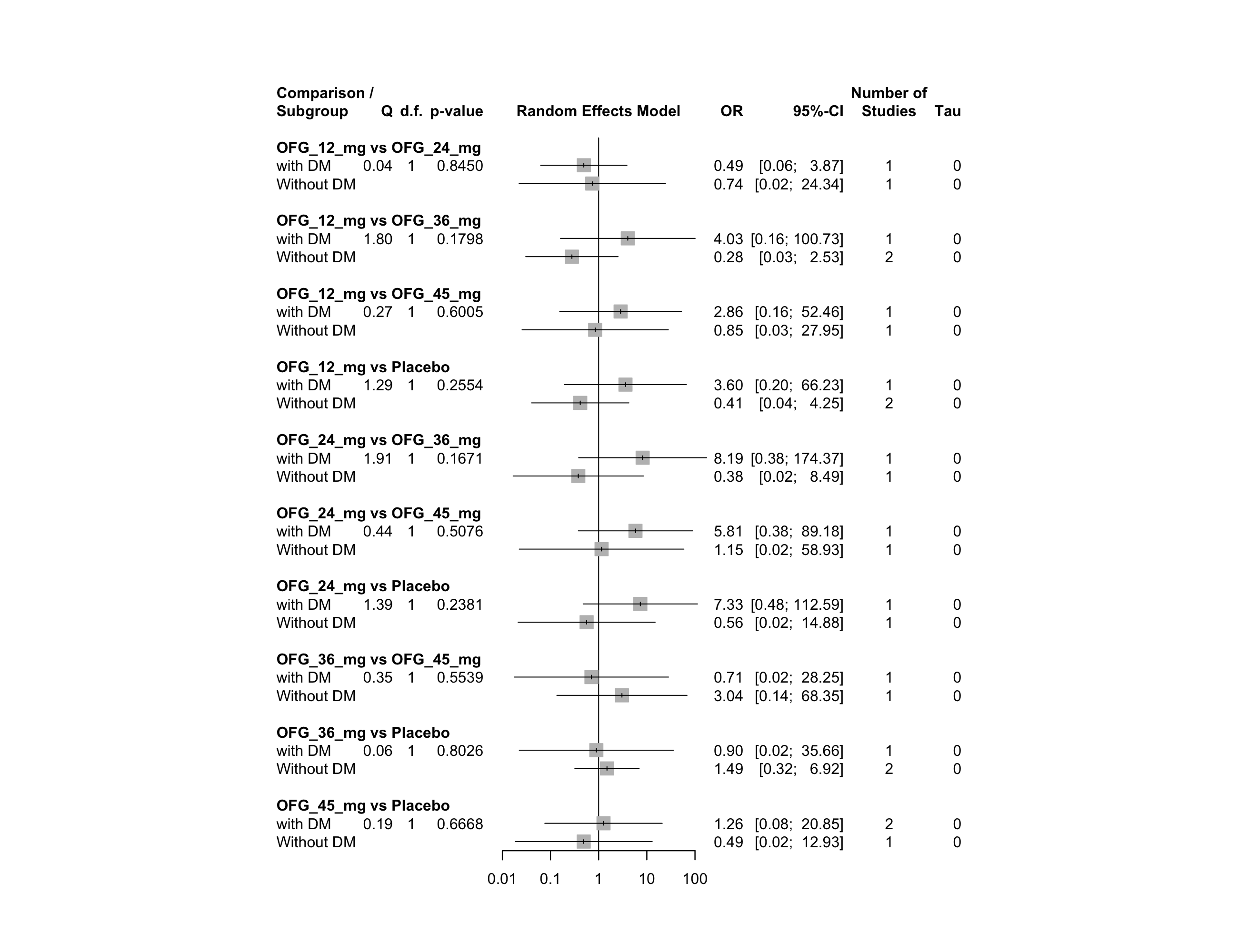


**Figure S24.** Subgroup analysis for GERD.


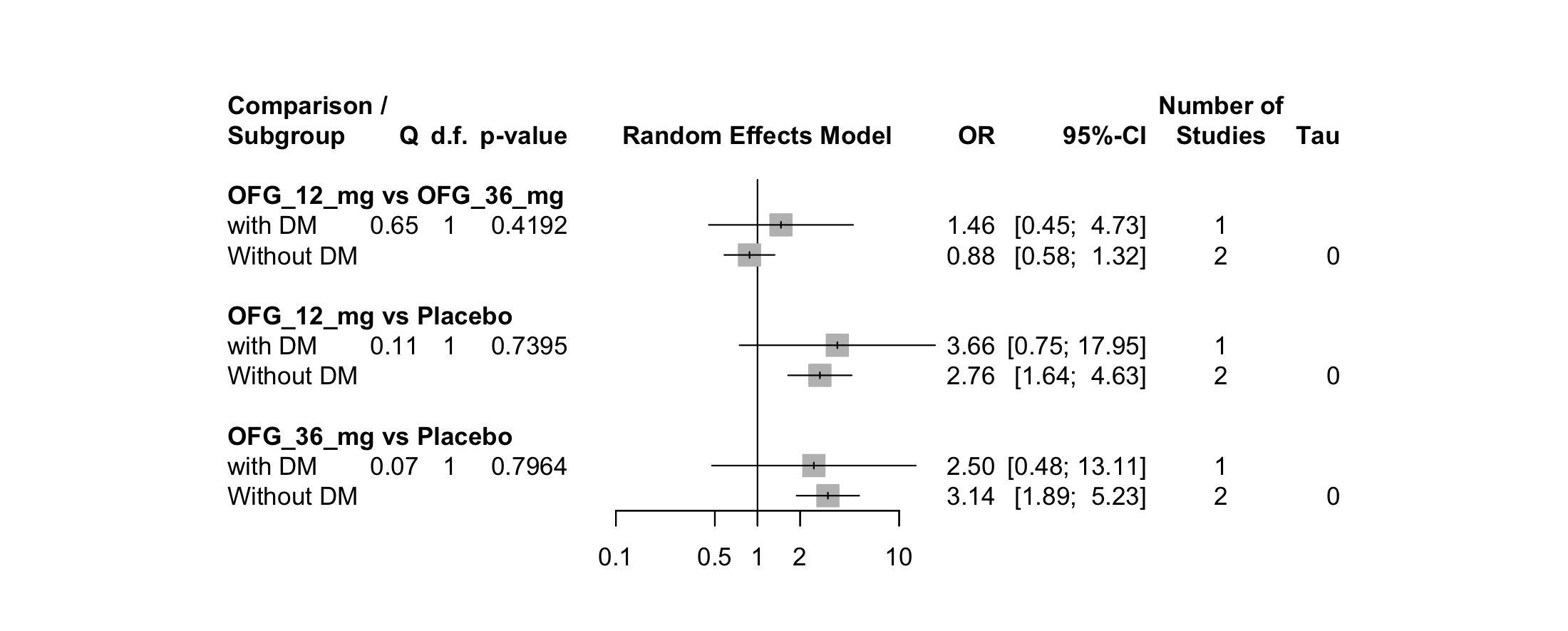


**Figure S25.** Subgroup analysis for abdominal pain.


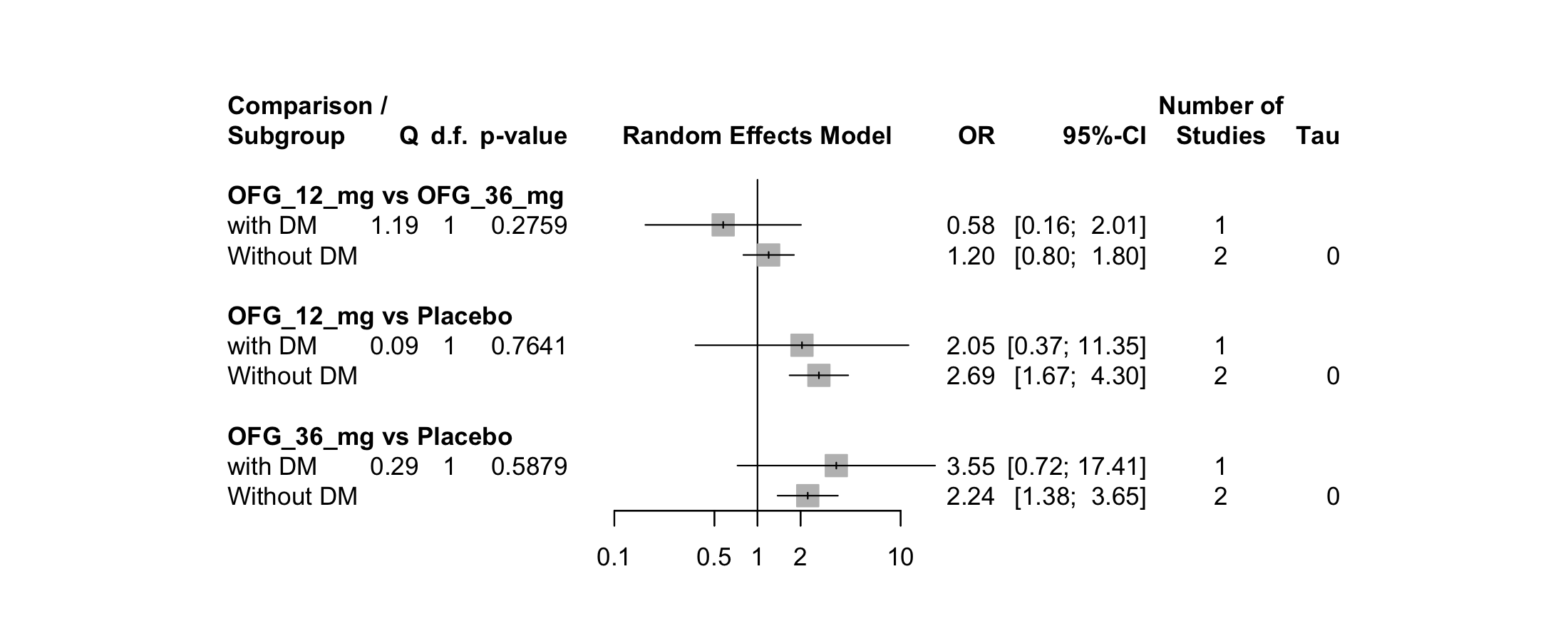


**Figure S26.** Subgroup analysis for pancreatitis.


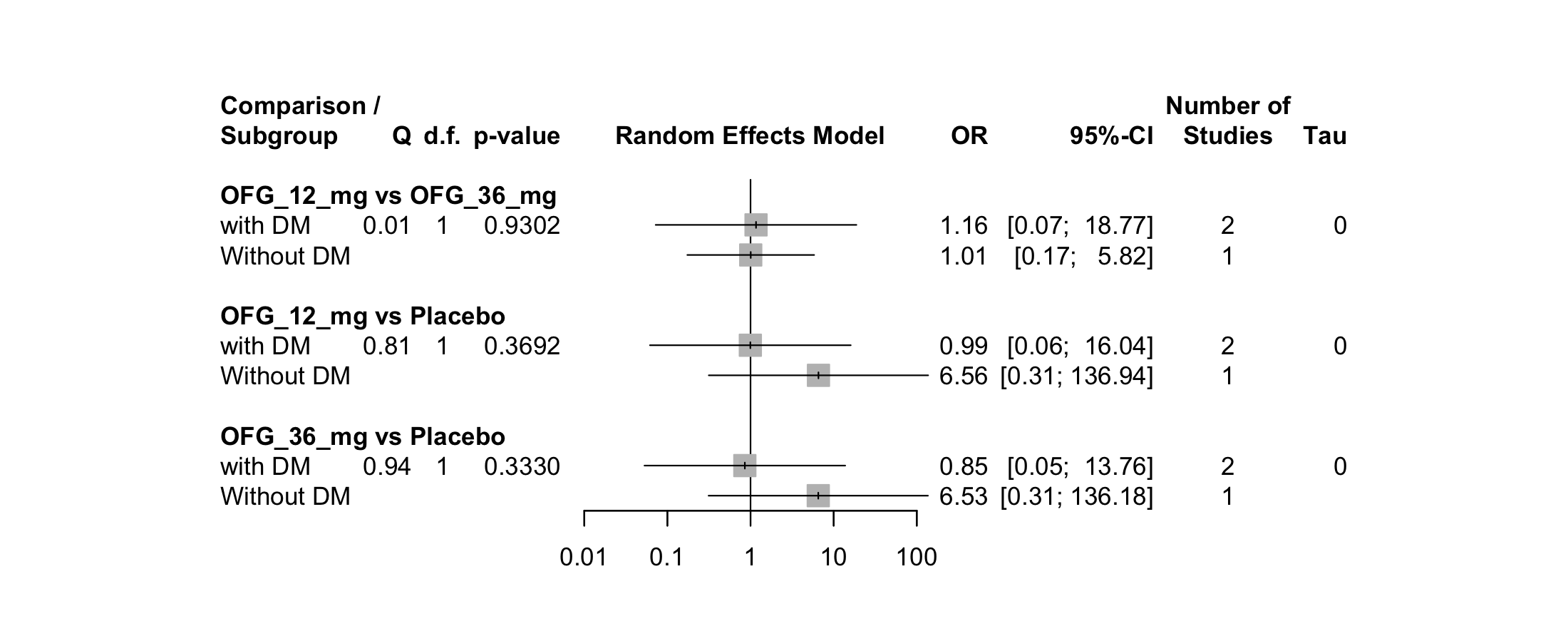


**Figure S27.** Subgroup analysis for abdominal distension.


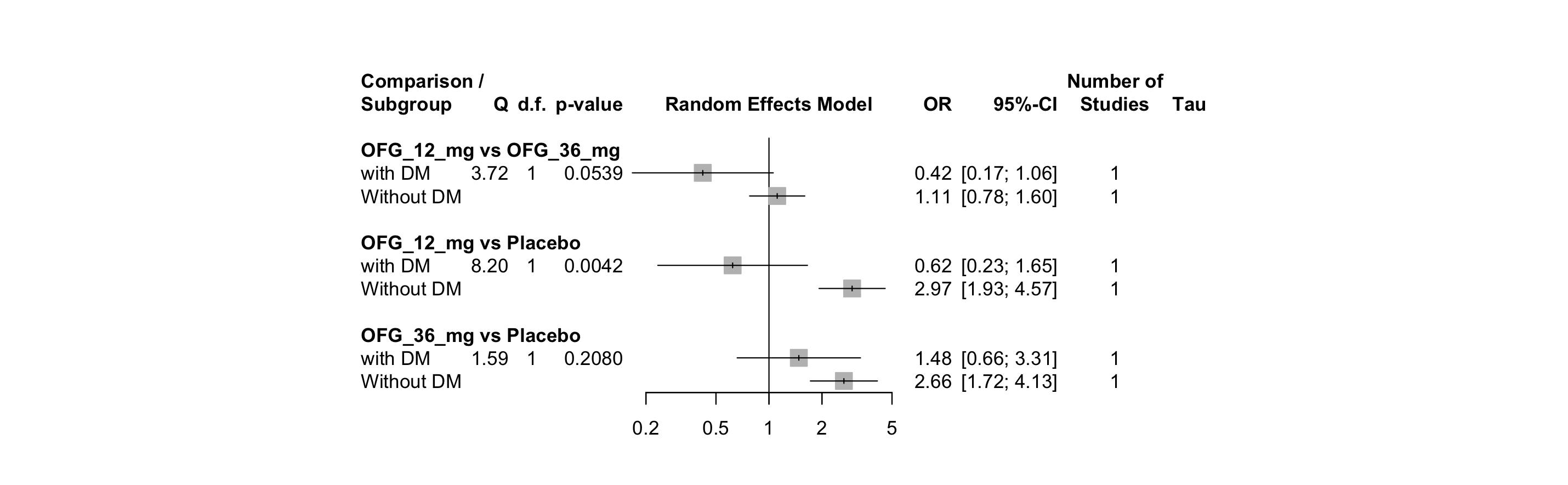


**Figure S28.** Subgroup analysis for % change from baseline in ALT at week 26.


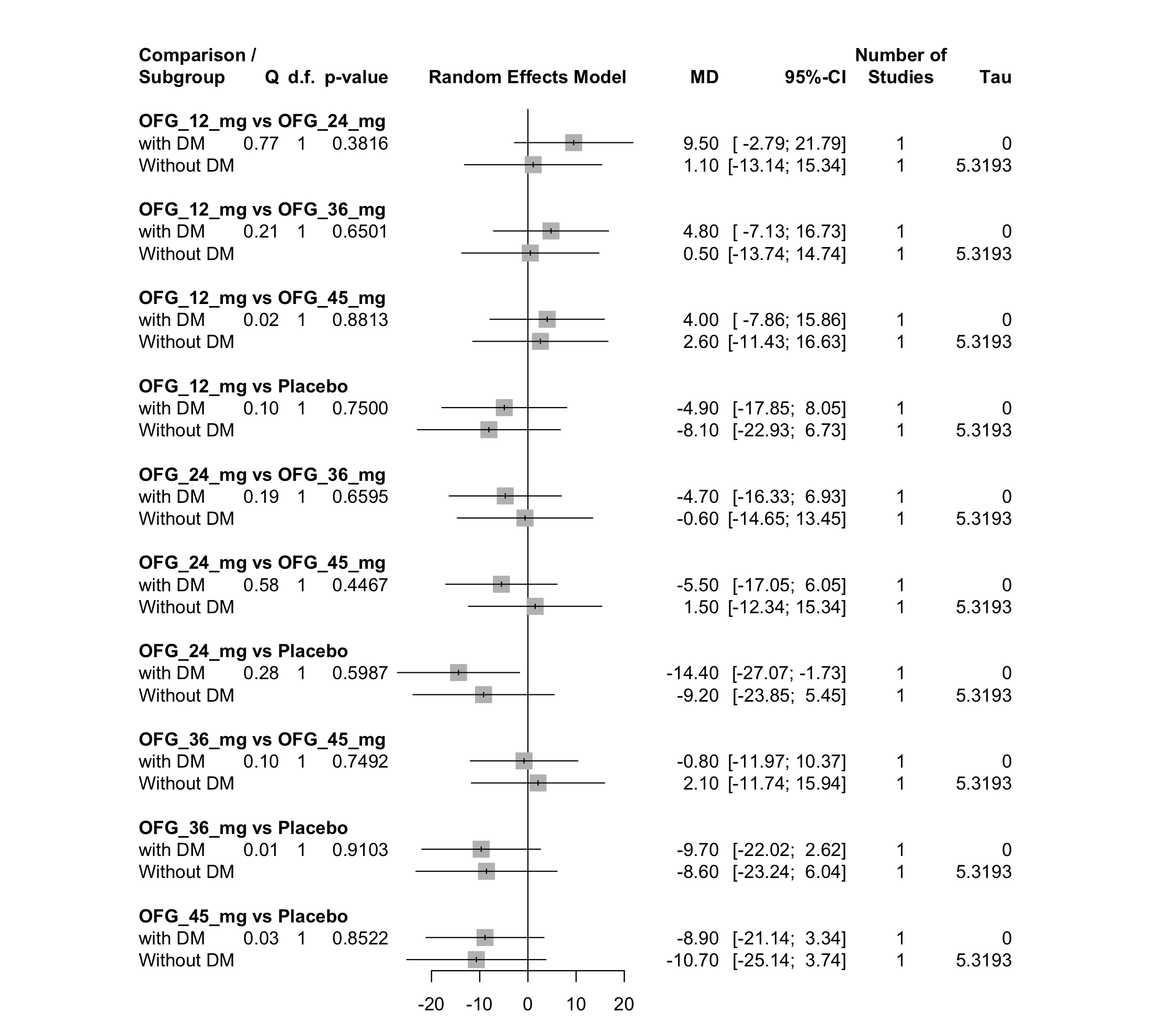


**Figure S29.** Subgroup analysis for % change from baseline in AST at week 26.


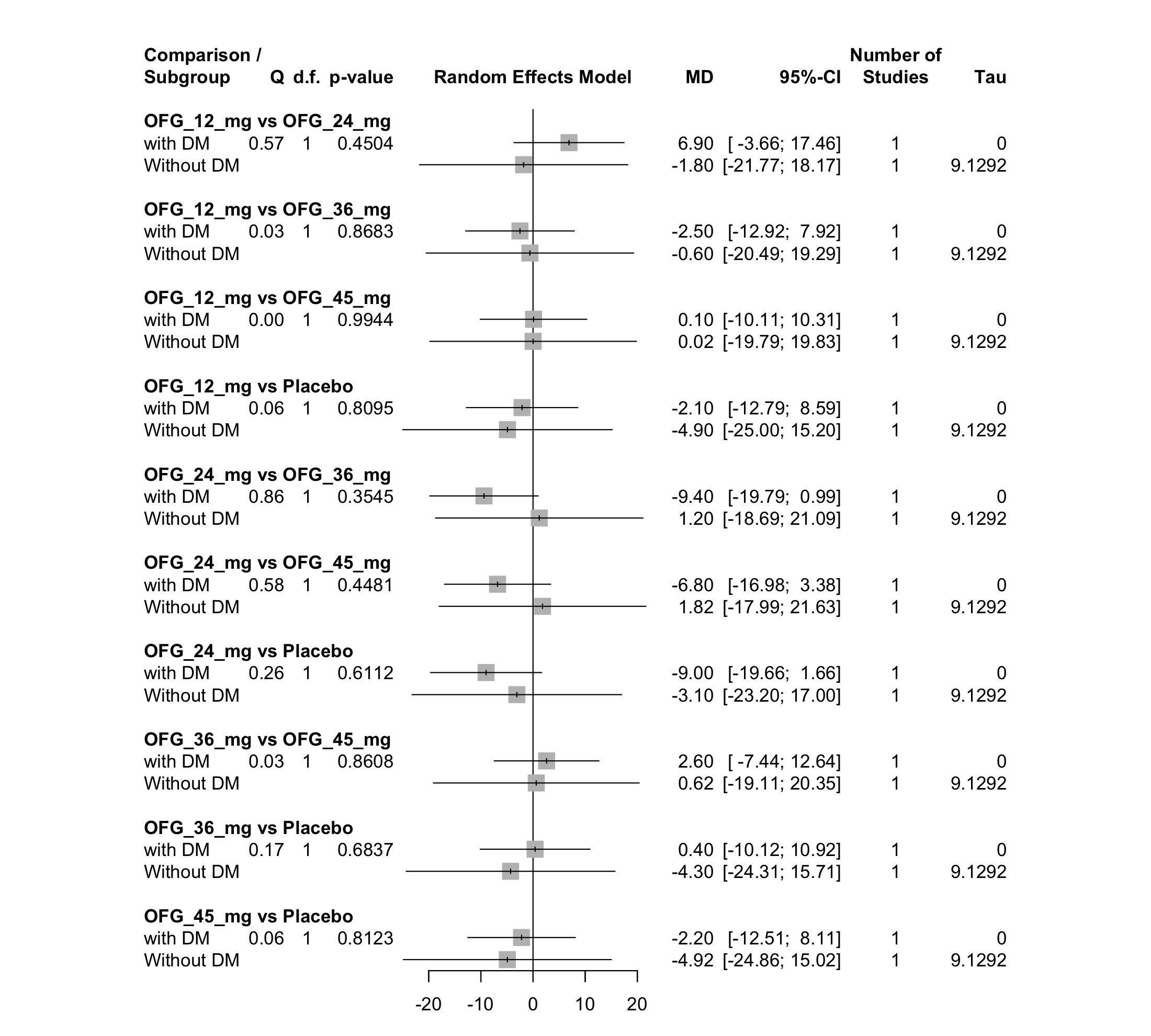


**Figure S30.** Subgroup analysis for % change from baseline in pancreatic amylase at week 26.


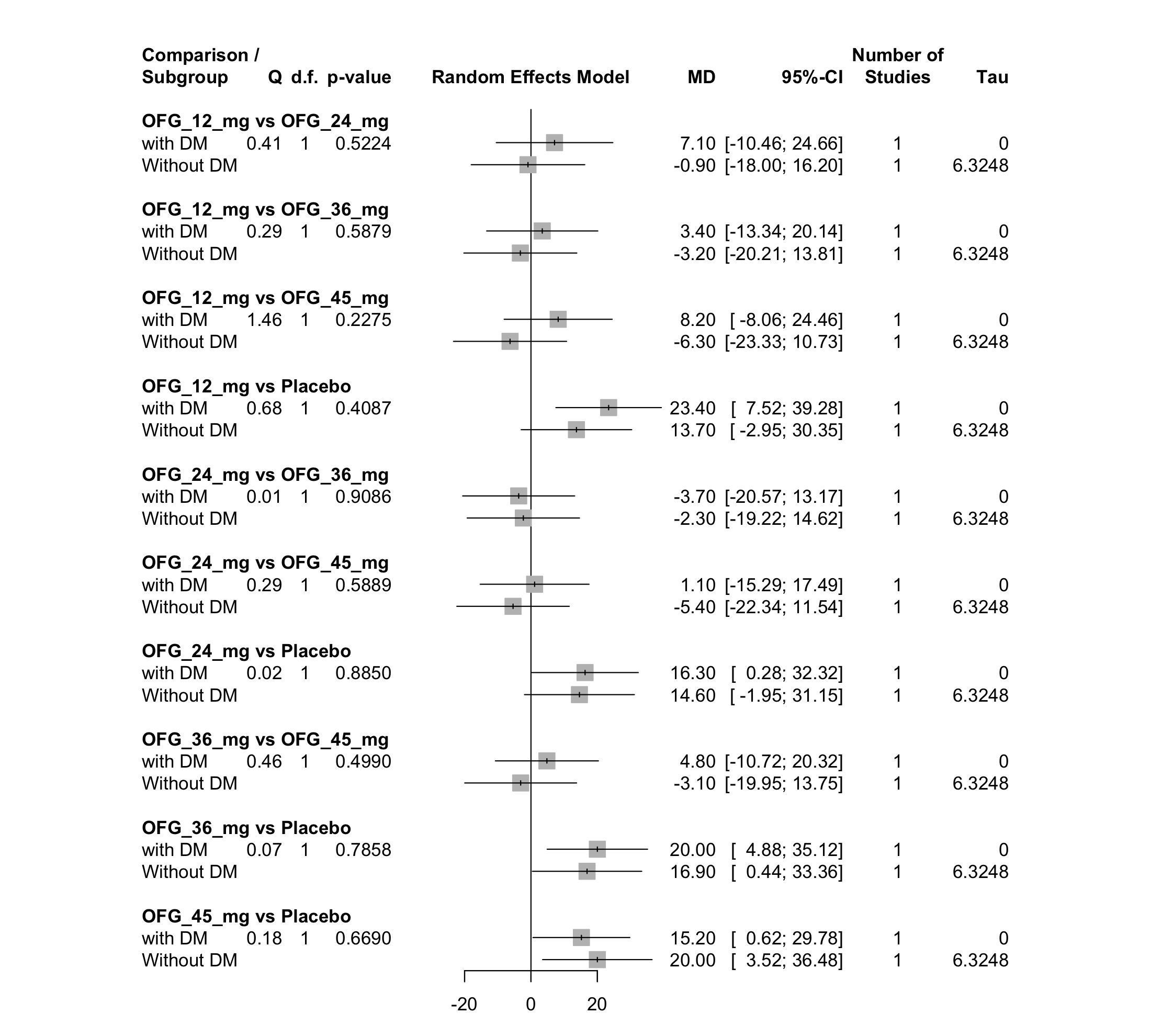


**Figure S31.** Subgroup analysis for % change from baseline in pancreatic lipase at week 26.


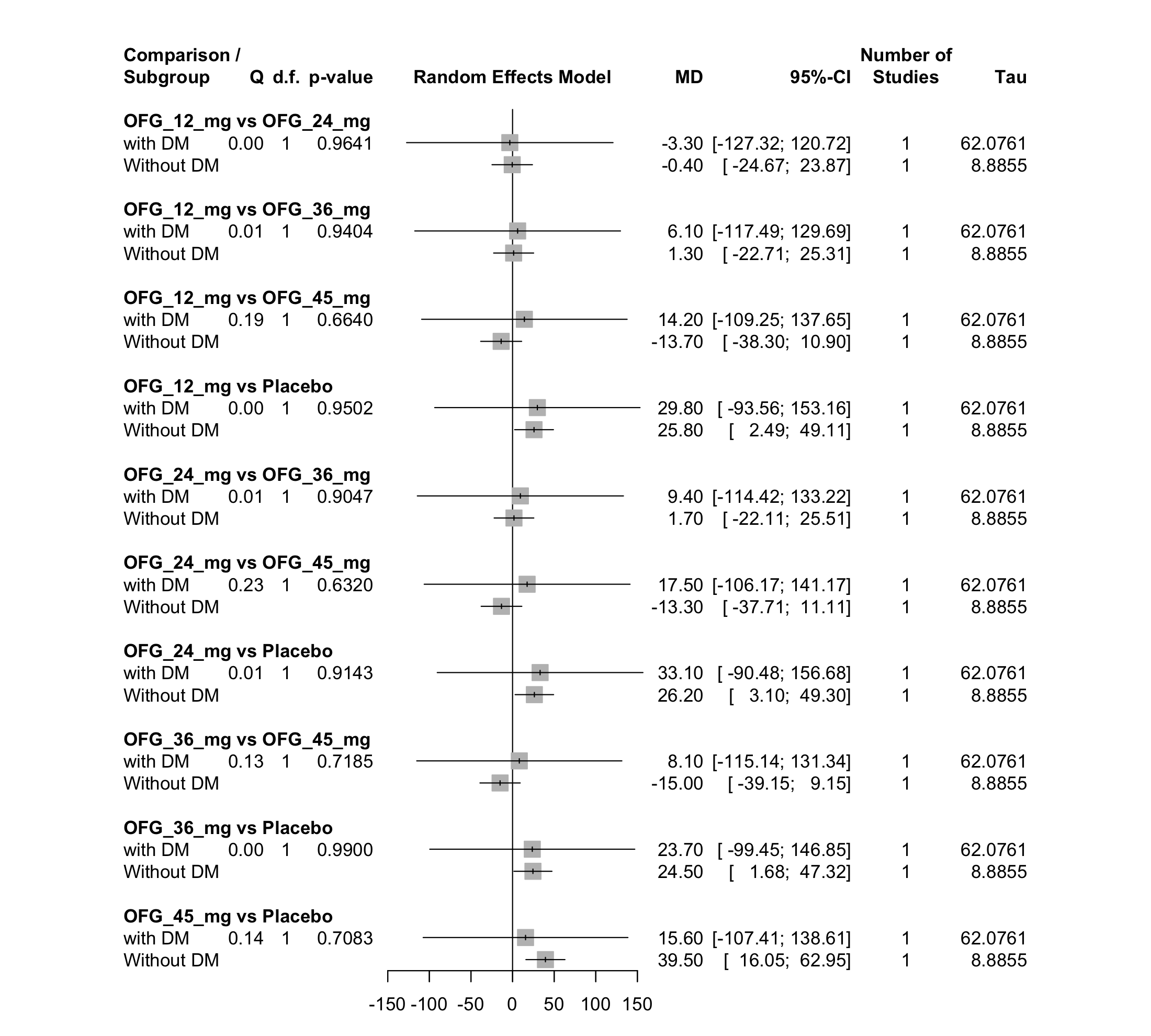


**Figure S32.** Subgroup analysis for % change from baseline in ALP at week 26.


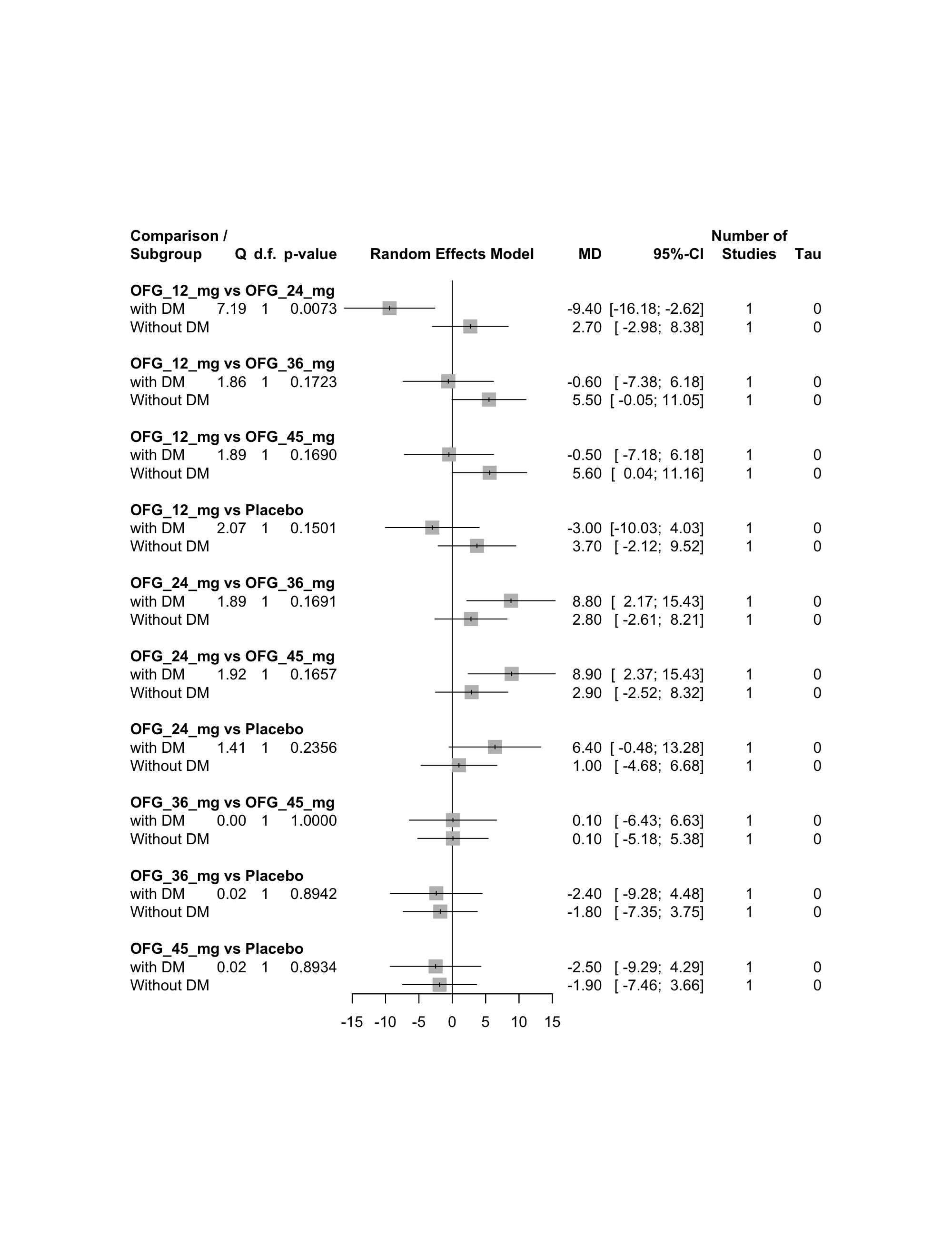


**Figure S33.** Side-splitting method for GI AEs leading to discontinuation.


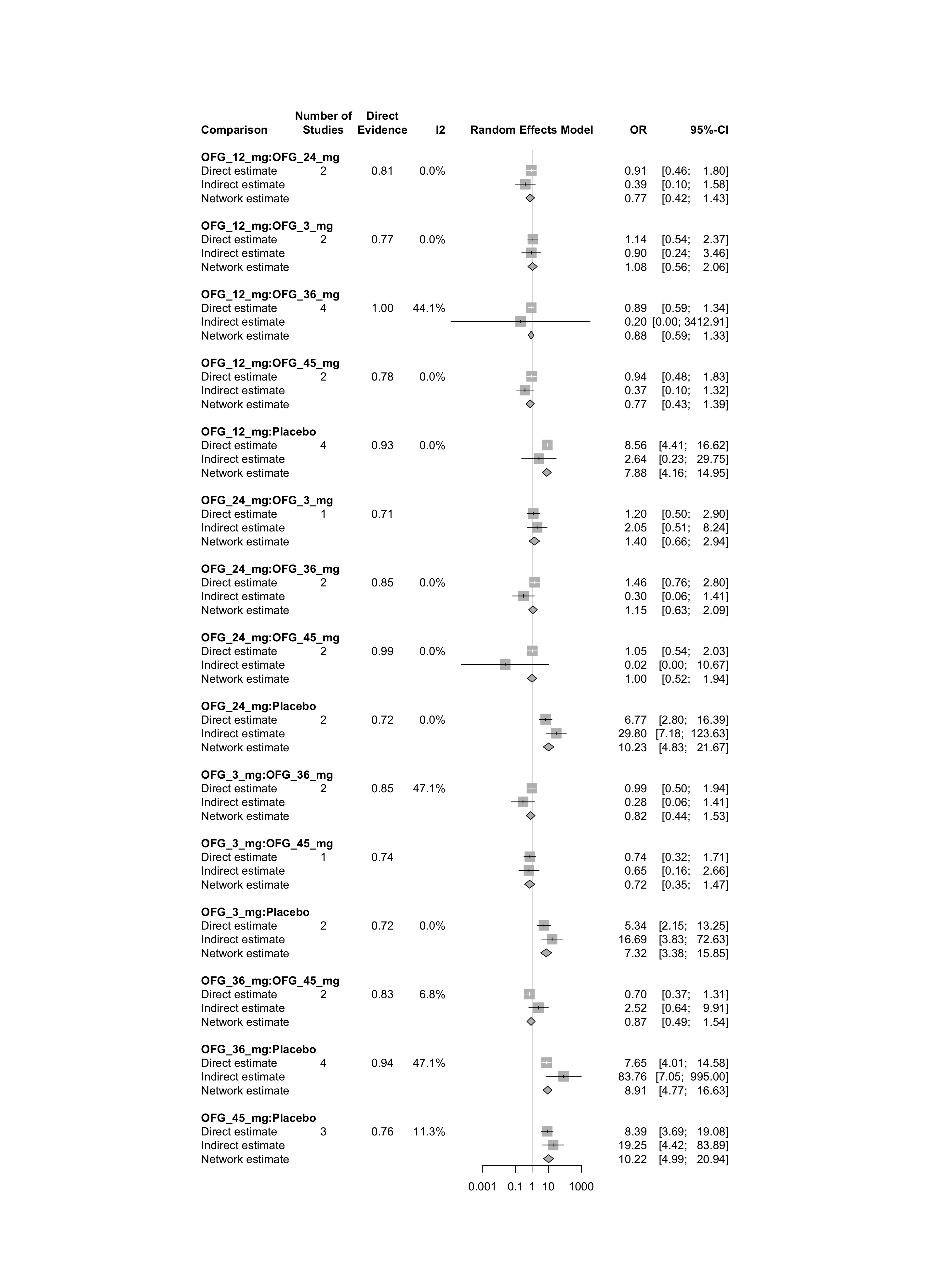


**Figure S34.** Side-splitting method for nausea.


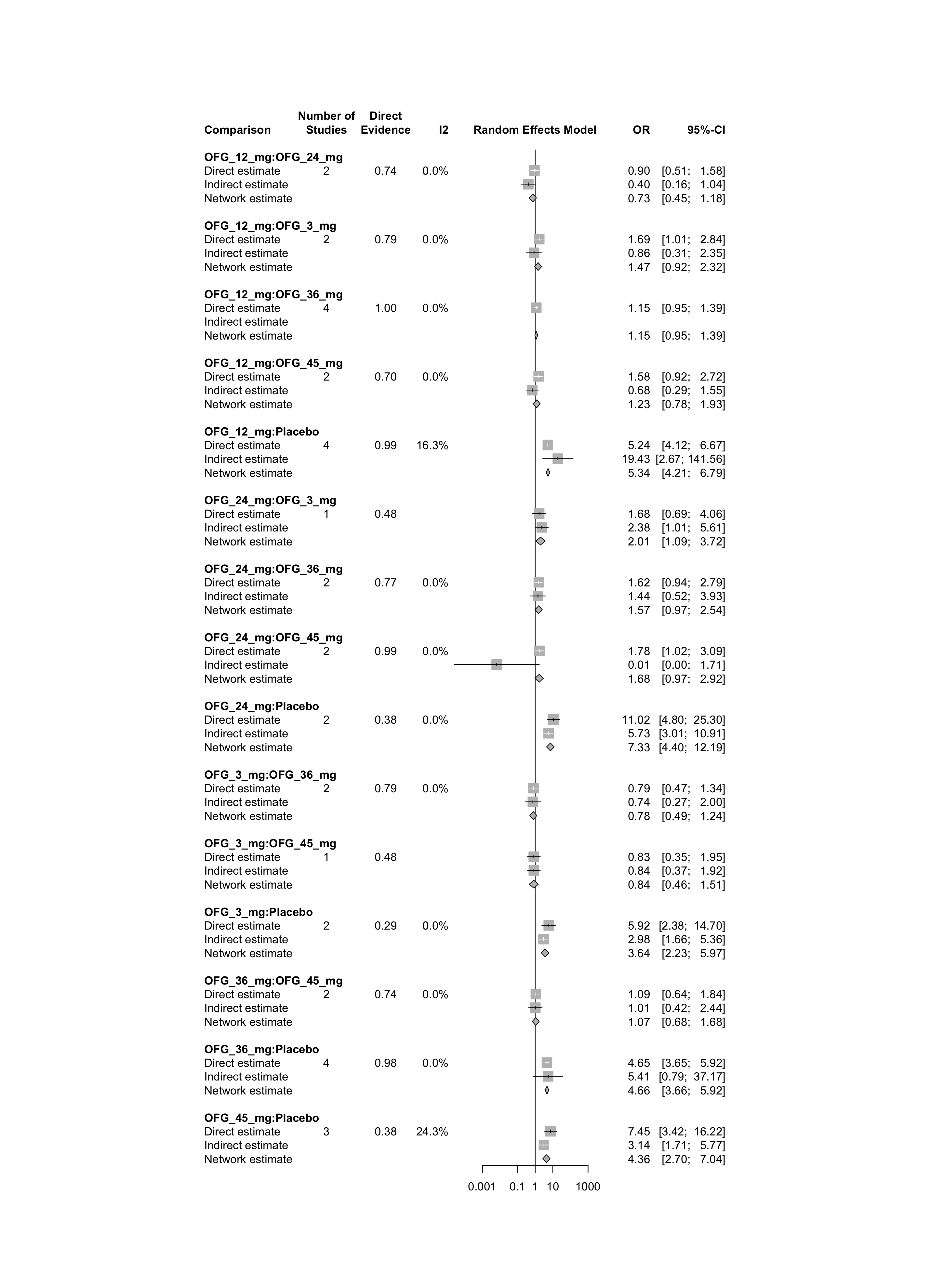


**Figure S35.** Side-splitting method for vomiting.


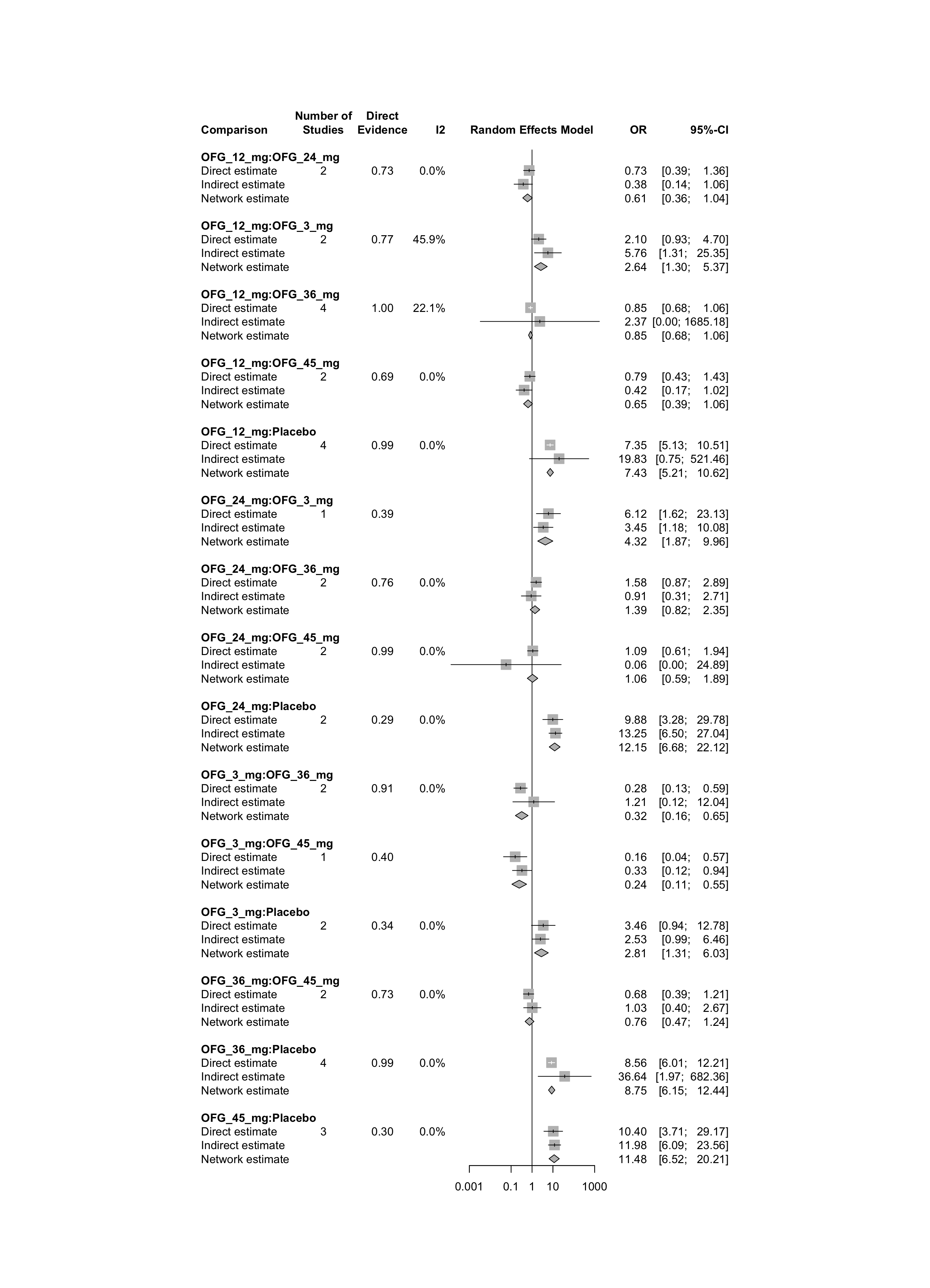


**Figure S36.** Side-splitting method for diarrhea.


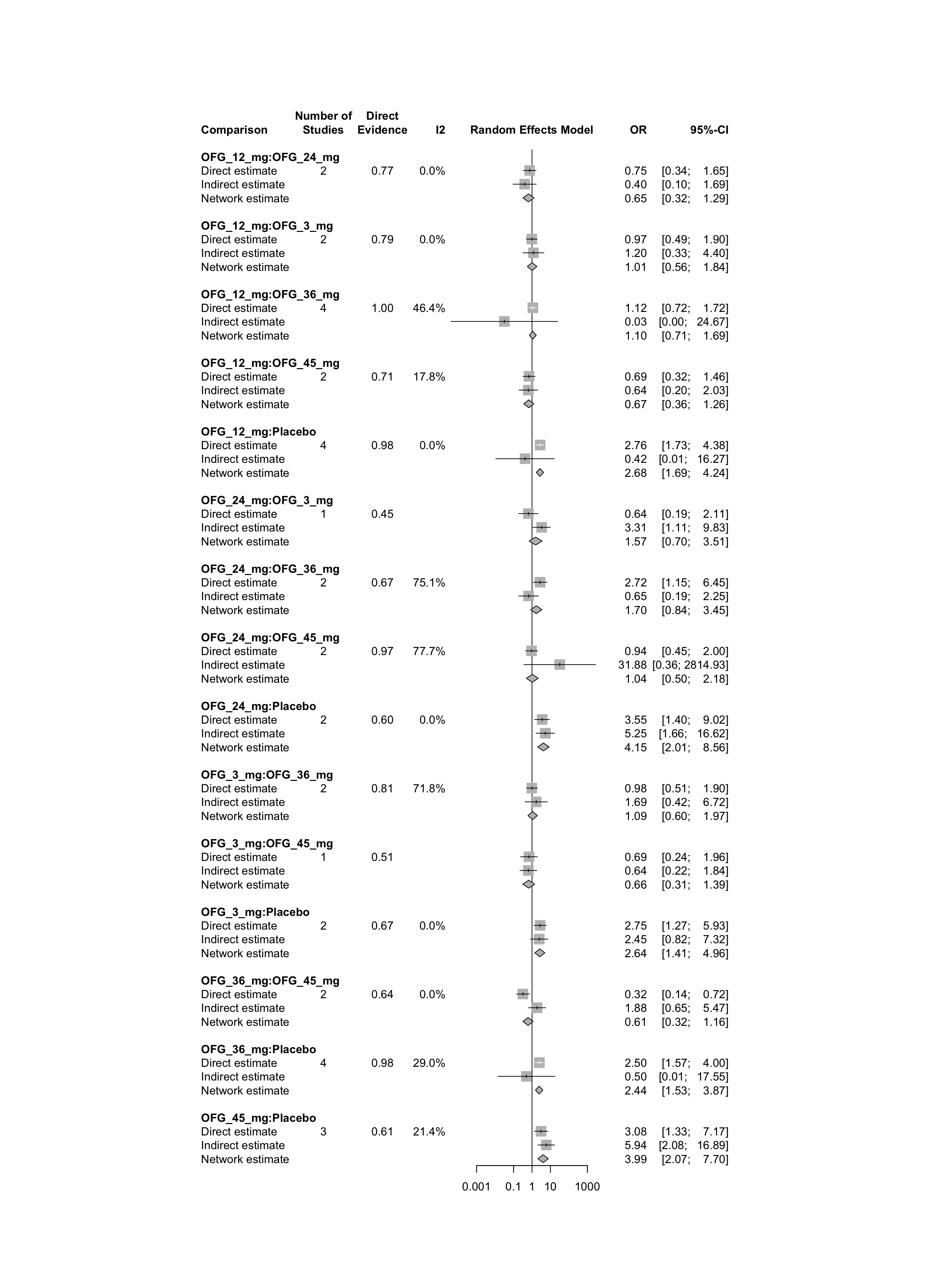


**Figure S37.** Side-splitting method for dyspepsia.


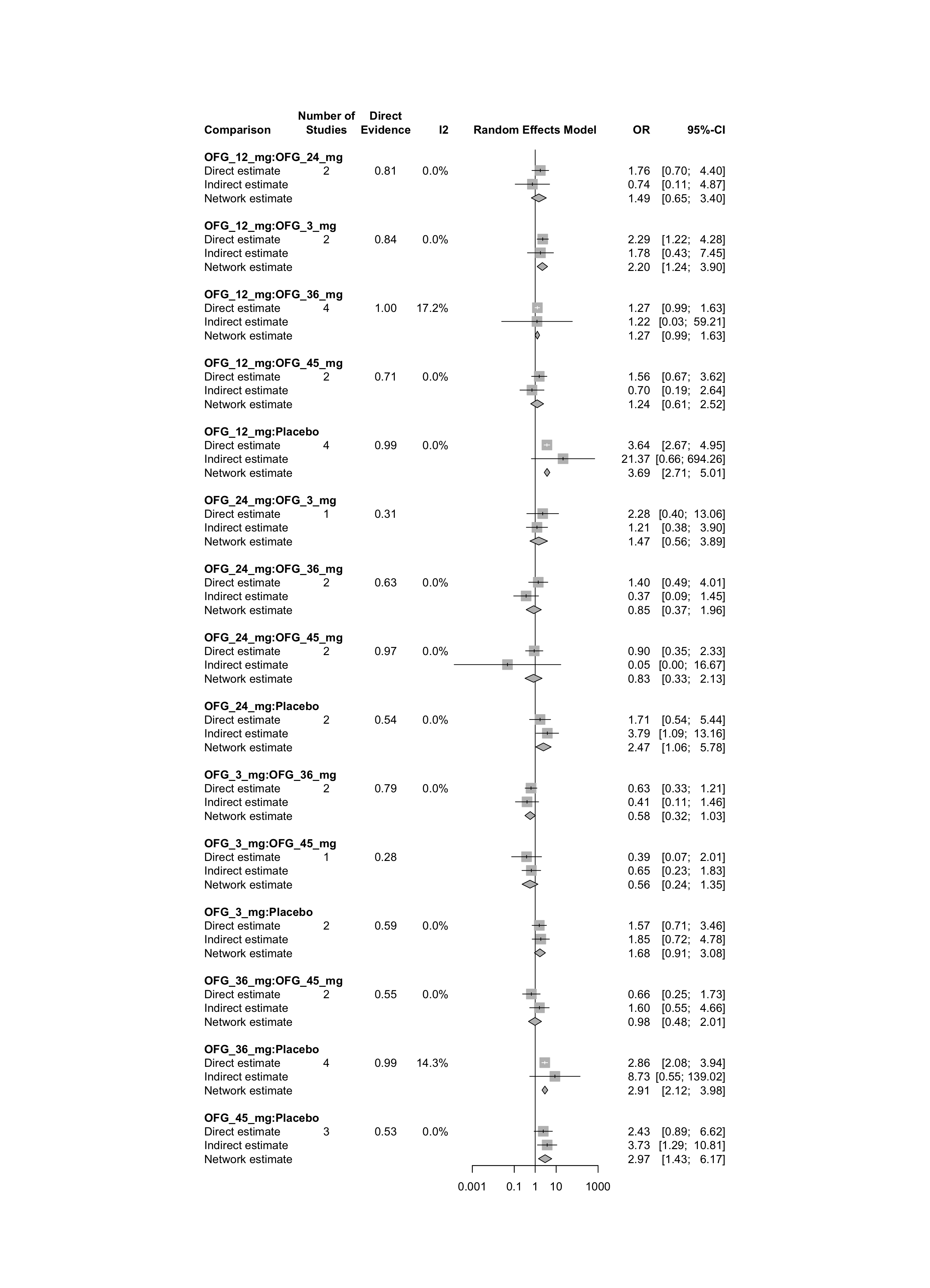


**Figure S38.** Side-splitting method for eructation.


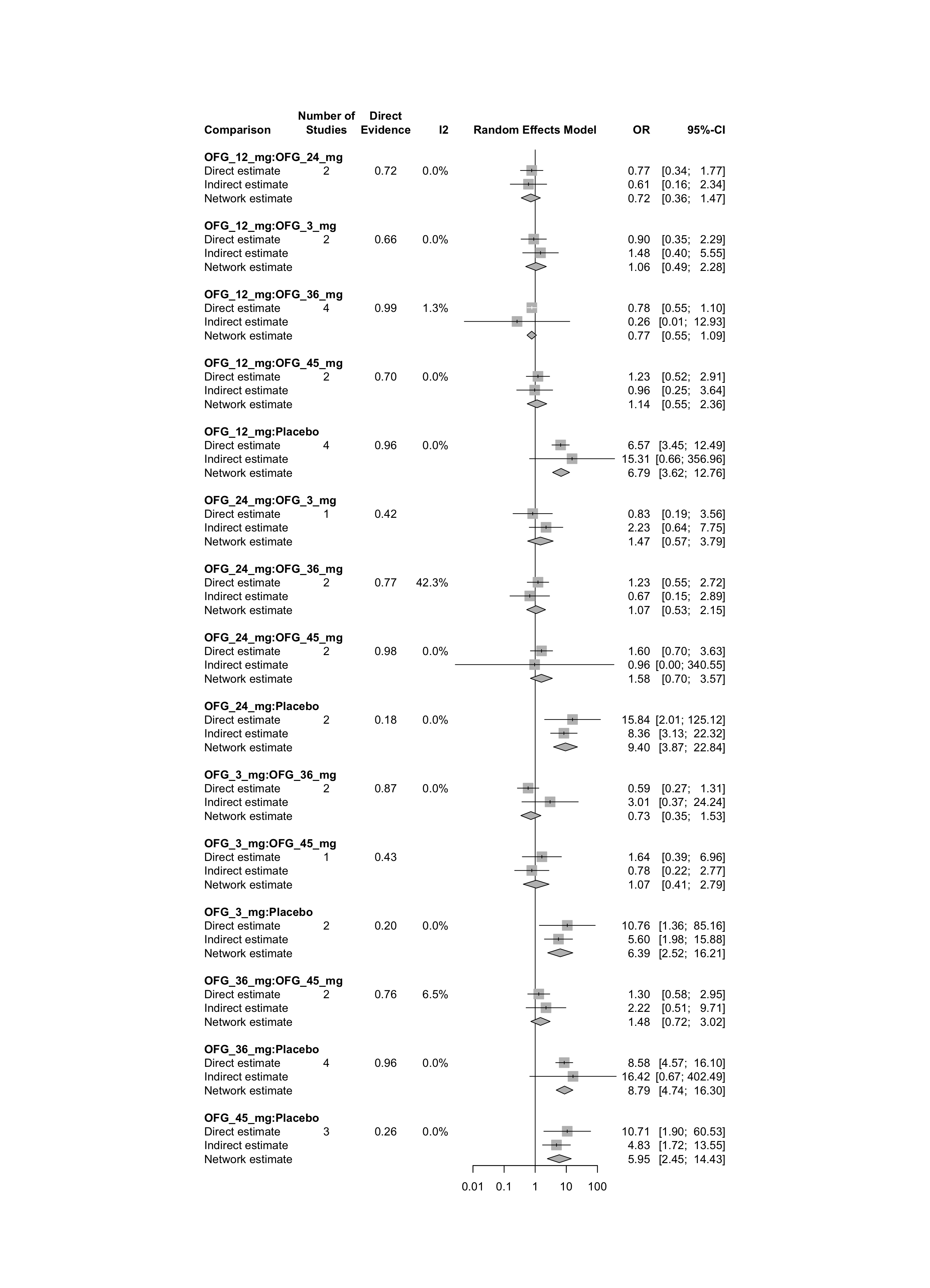


**Figure S39.** Side-splitting method for constipation.


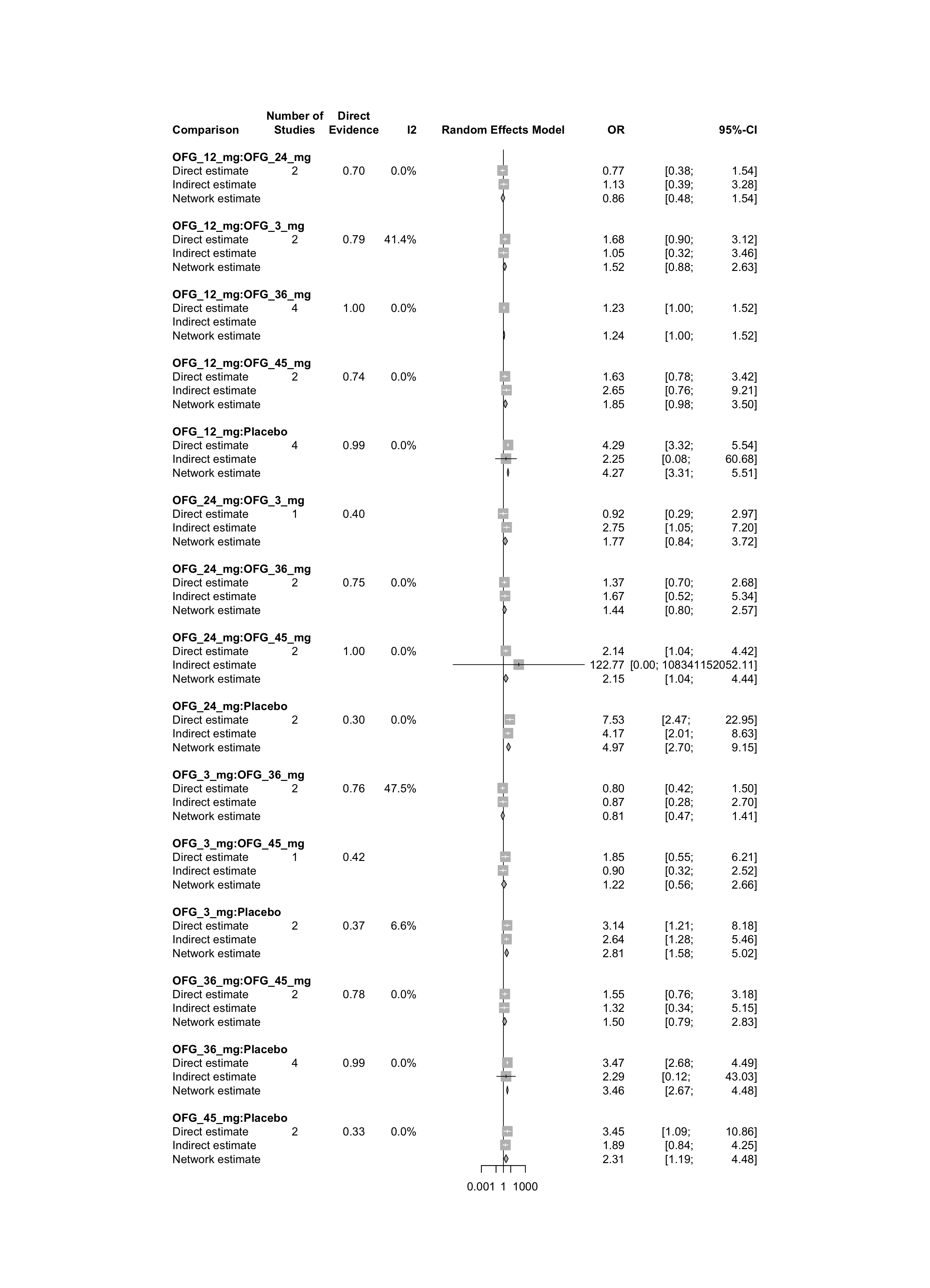


**Figure S40.** Side-splitting method for decreased appetite.


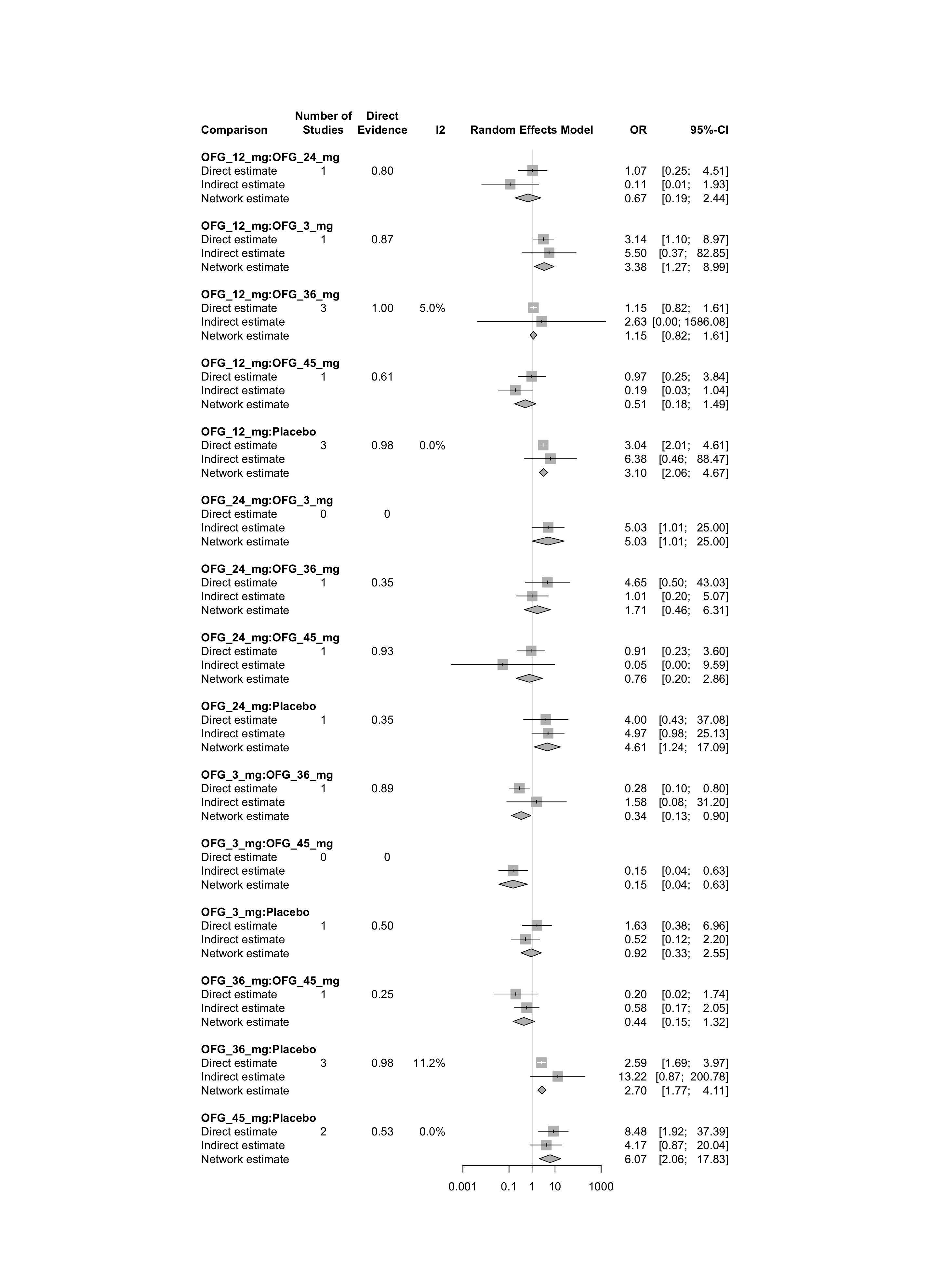


**Figure S41.** Side-splitting method for hepatic events.


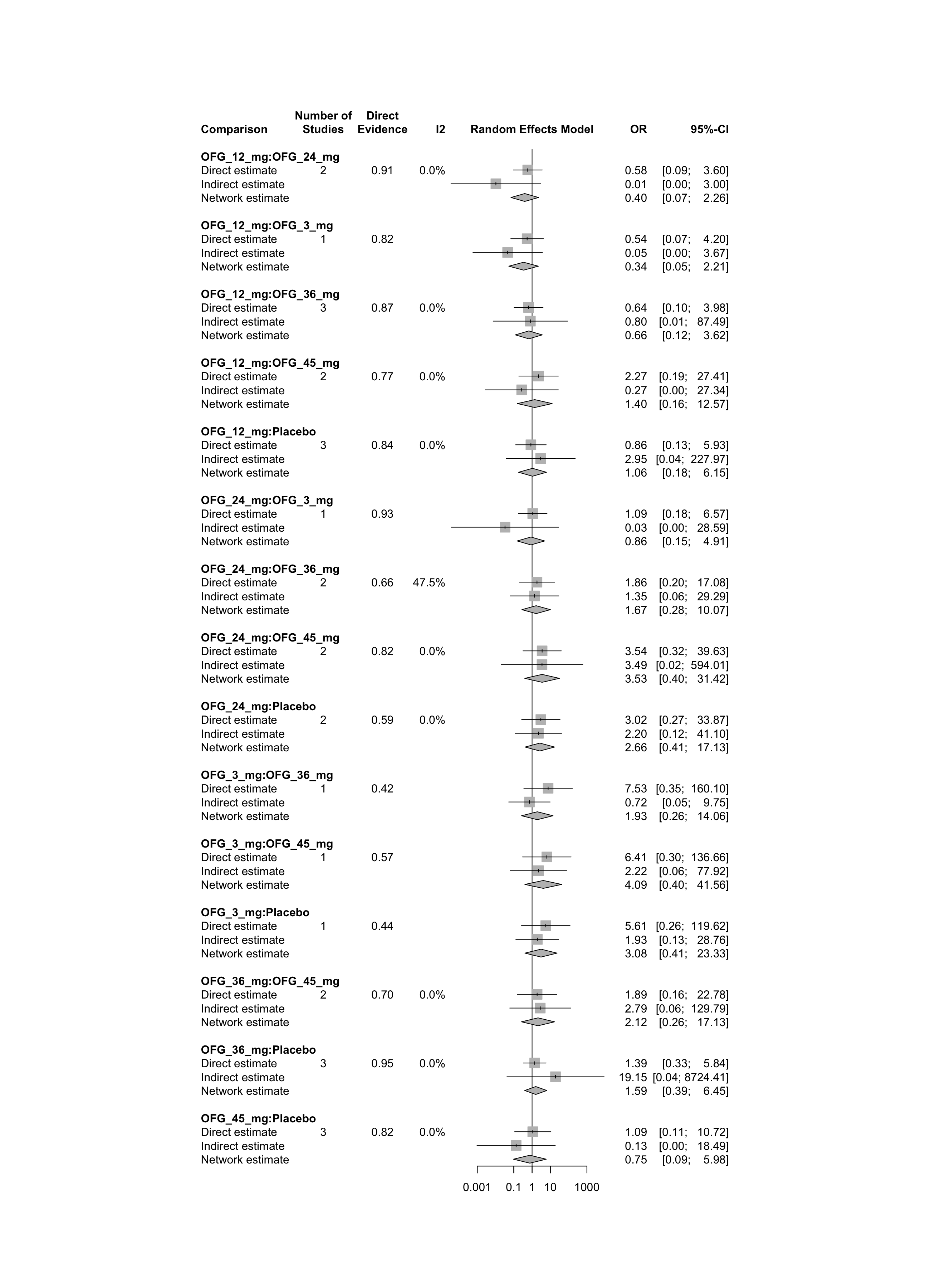


**Figure S42.** Side-splitting method for GERD.


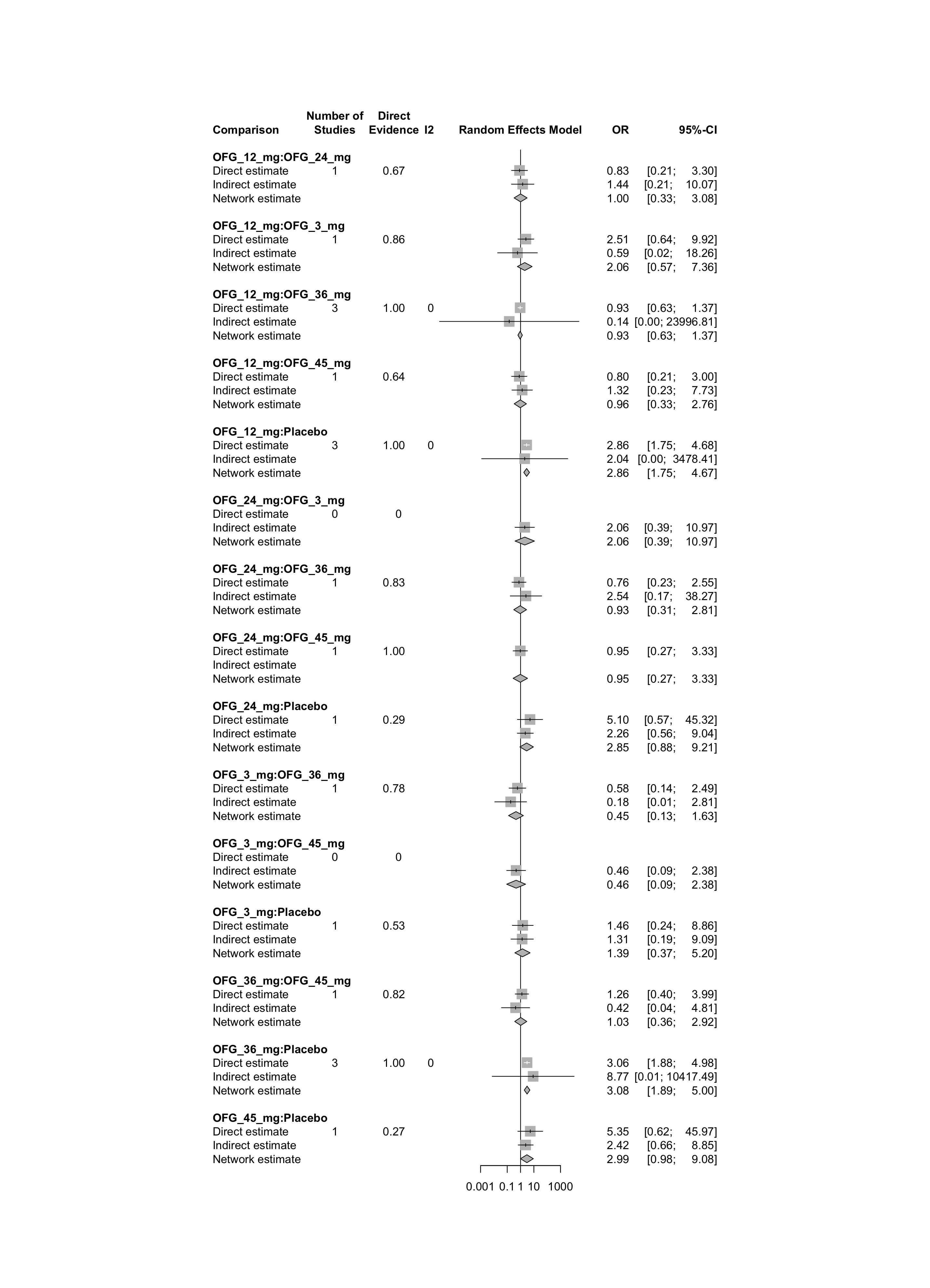


**Figure S43.** Side-splitting method for abdominal pain.


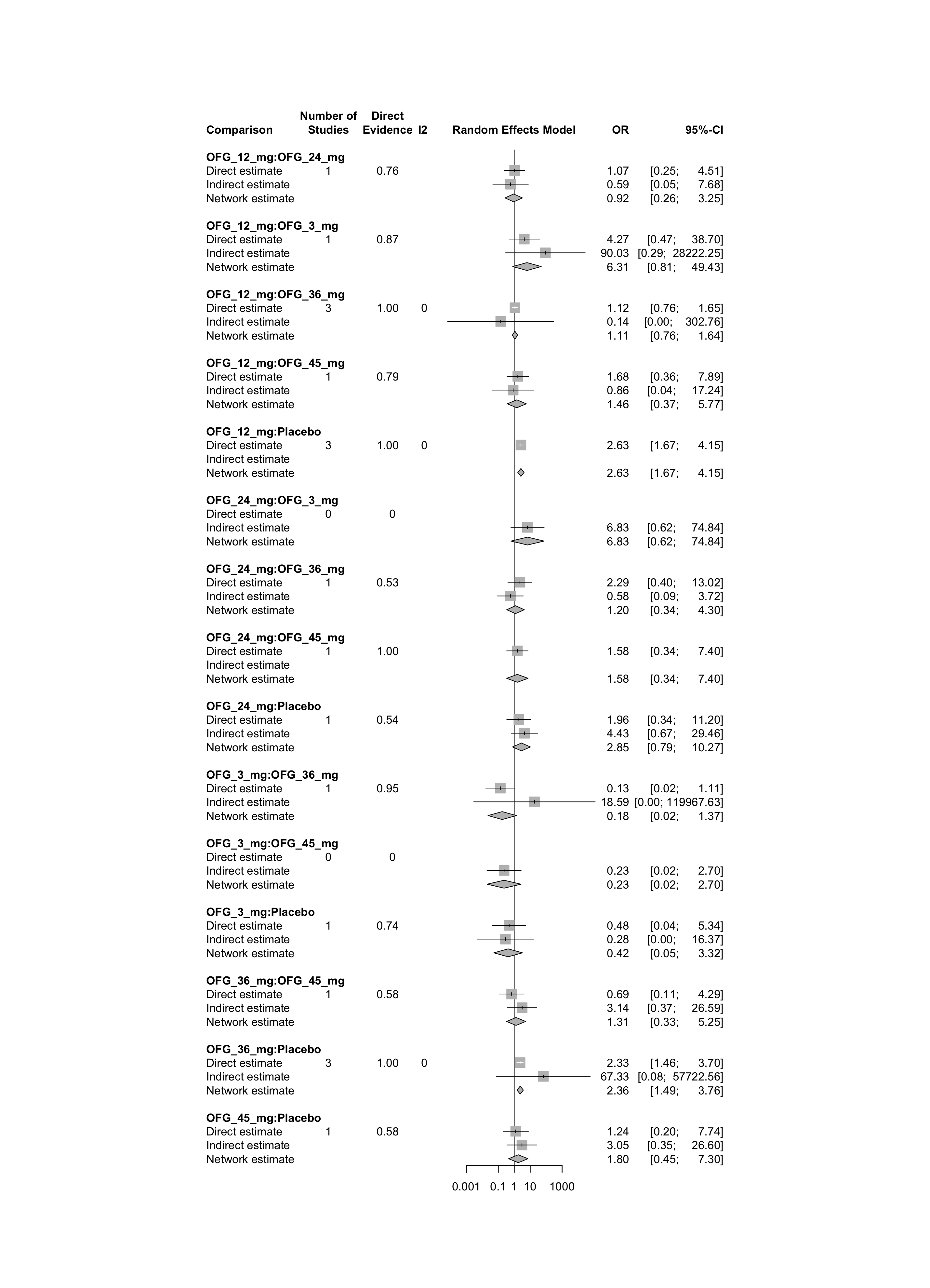


**Figure S44.** Side-splitting method for pancreatitis.


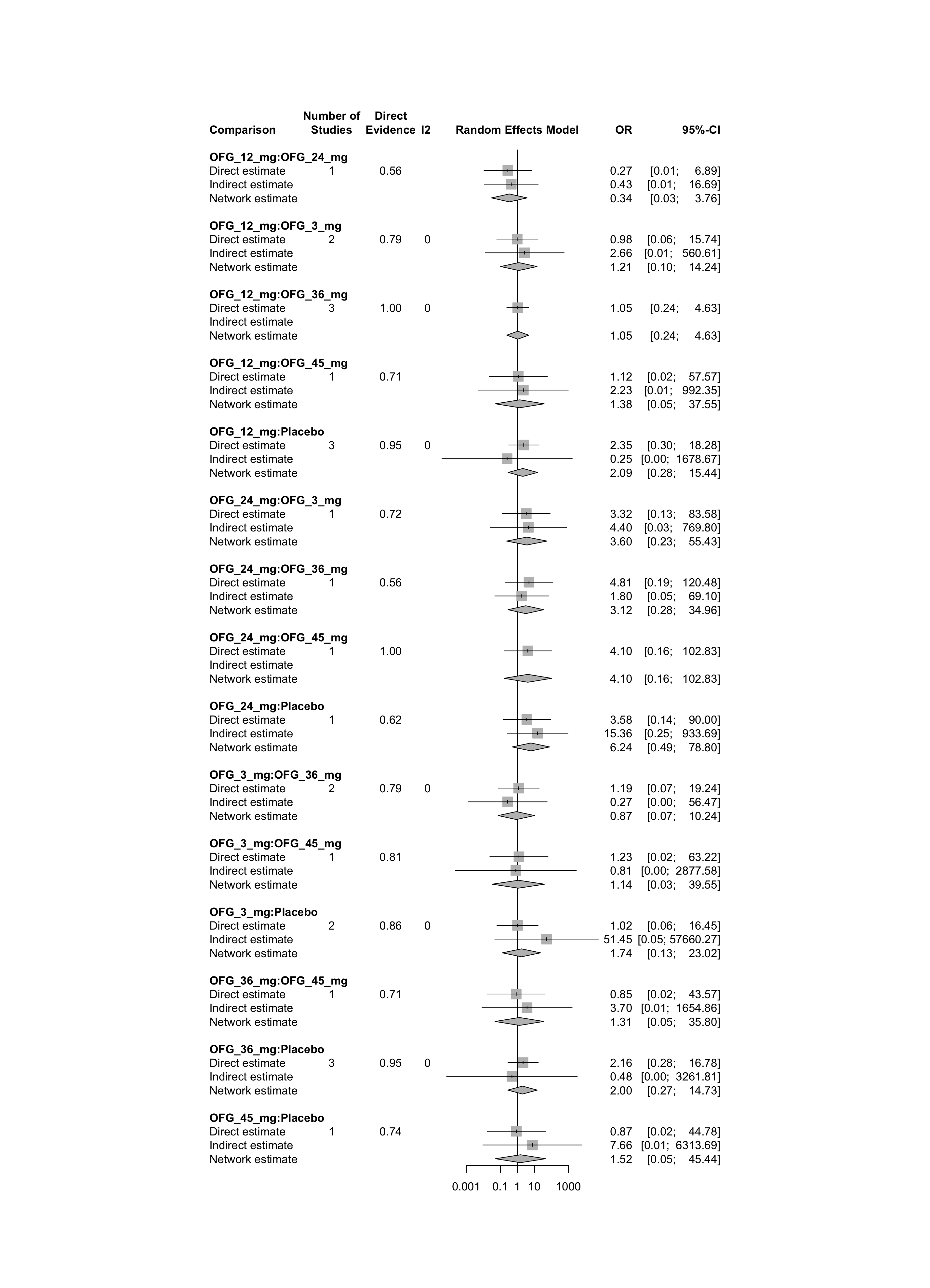


**Figure S45.** Side-splitting method for % change from baseline in ALT at week 26.


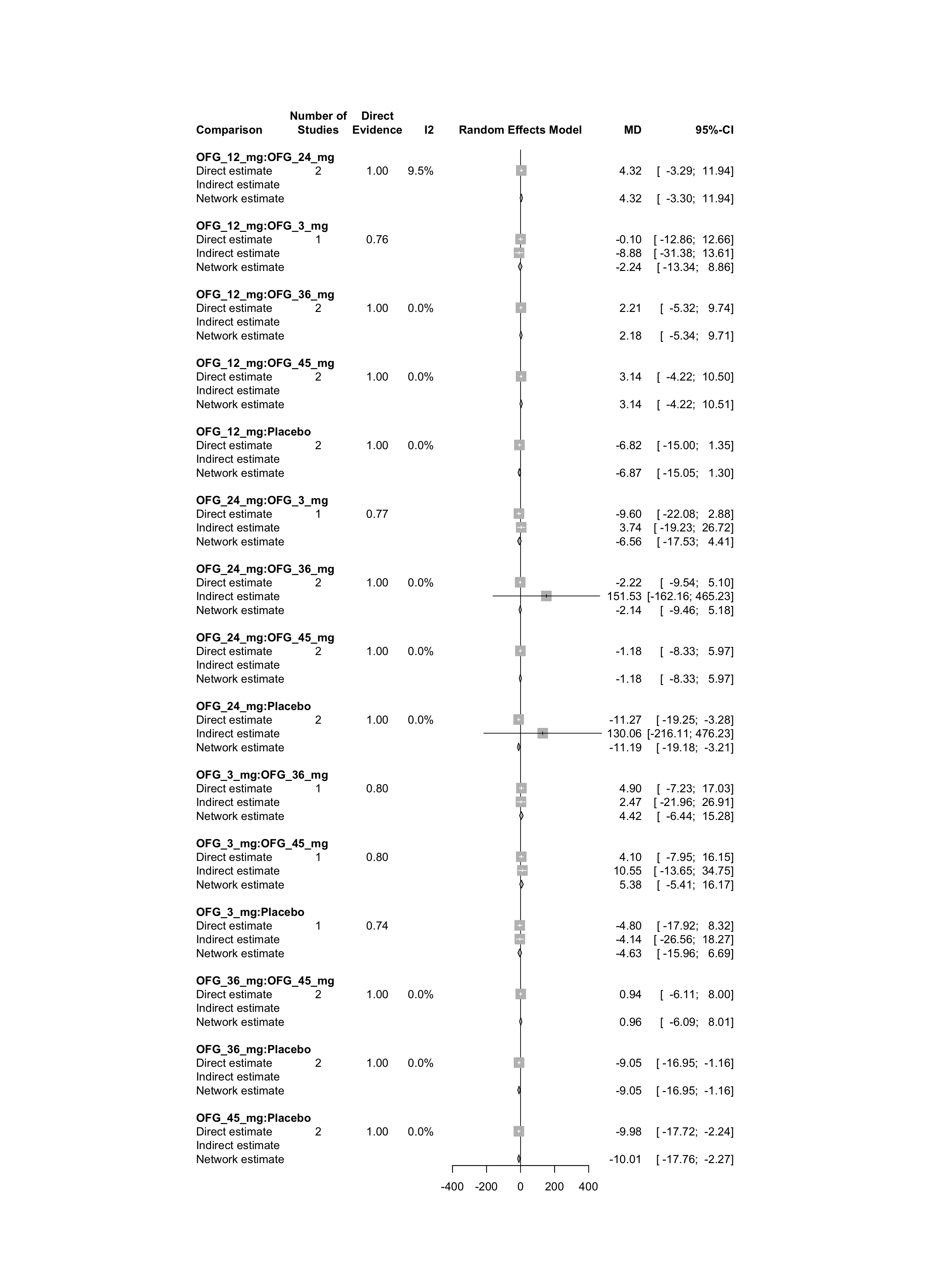


**Figure S46**. Side-splitting method for % change from baseline in AST at week 26.


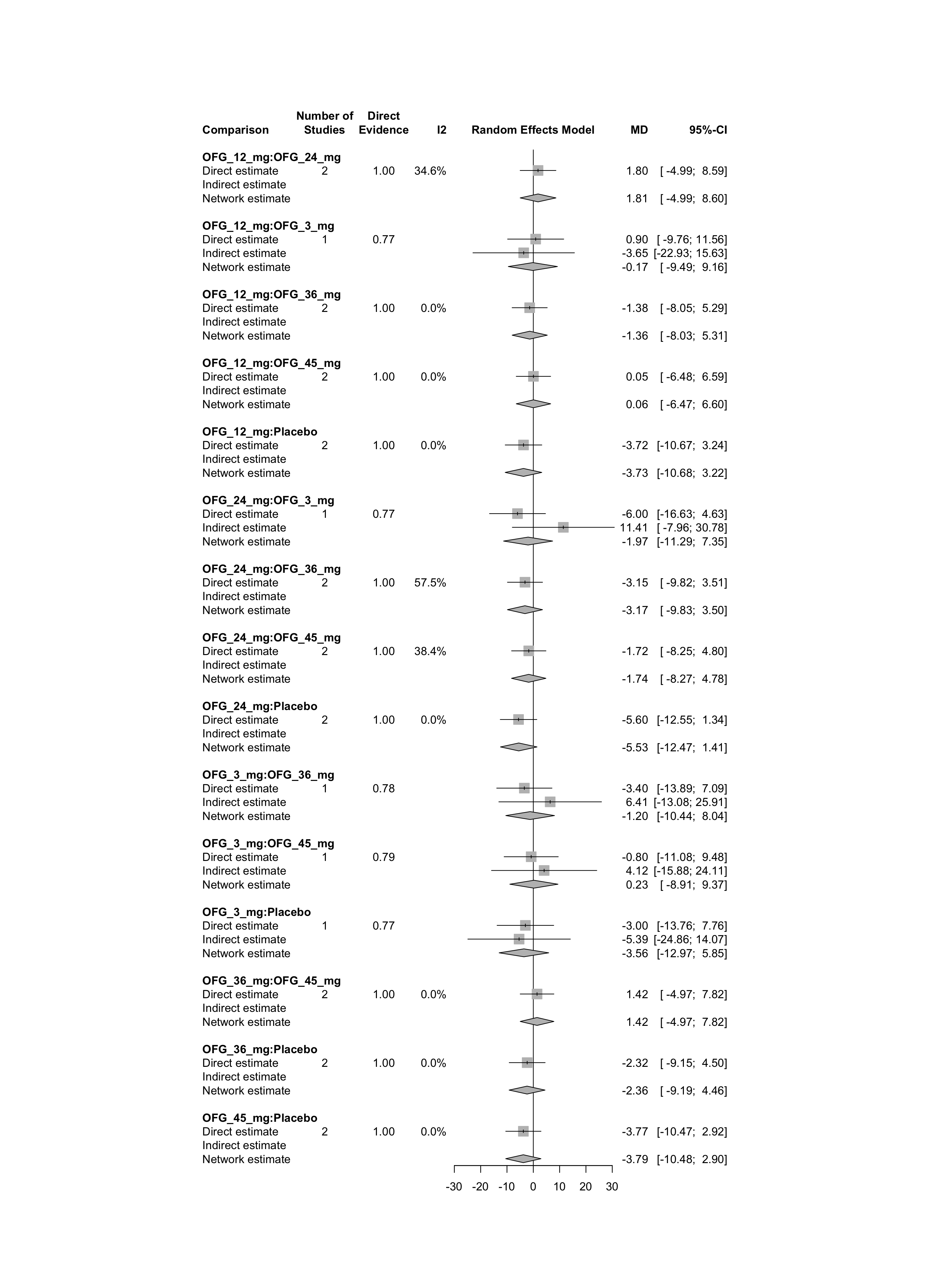


**Figure S47.** Side-splitting method for % change from baseline in pancreatic amylase at week 26.


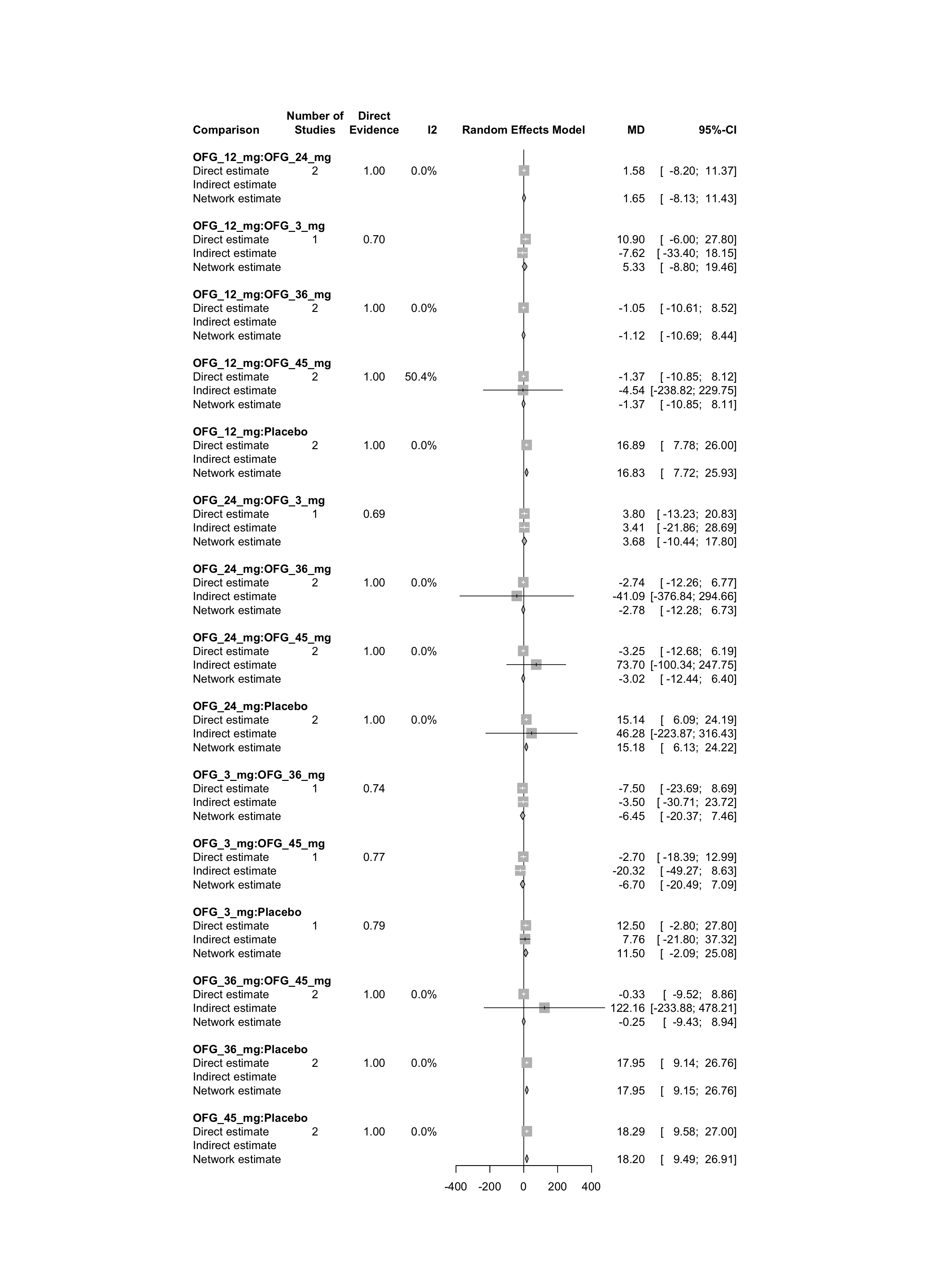


**Figure S48.** Side-splitting method for % change from baseline in total serum lipase at week 26.


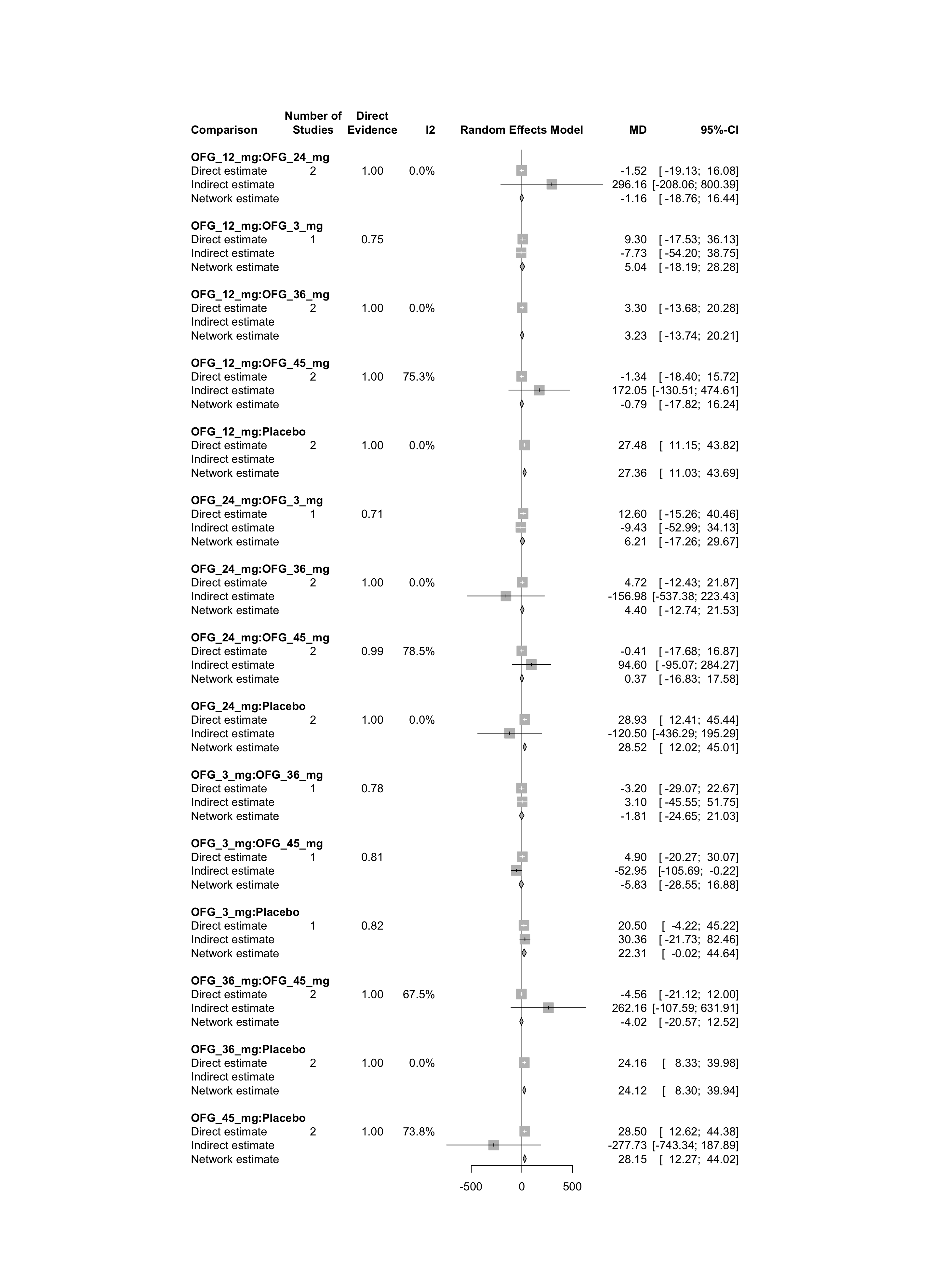


**Figure S49.** Side-splitting method for % change from baseline in ALP at week 26.


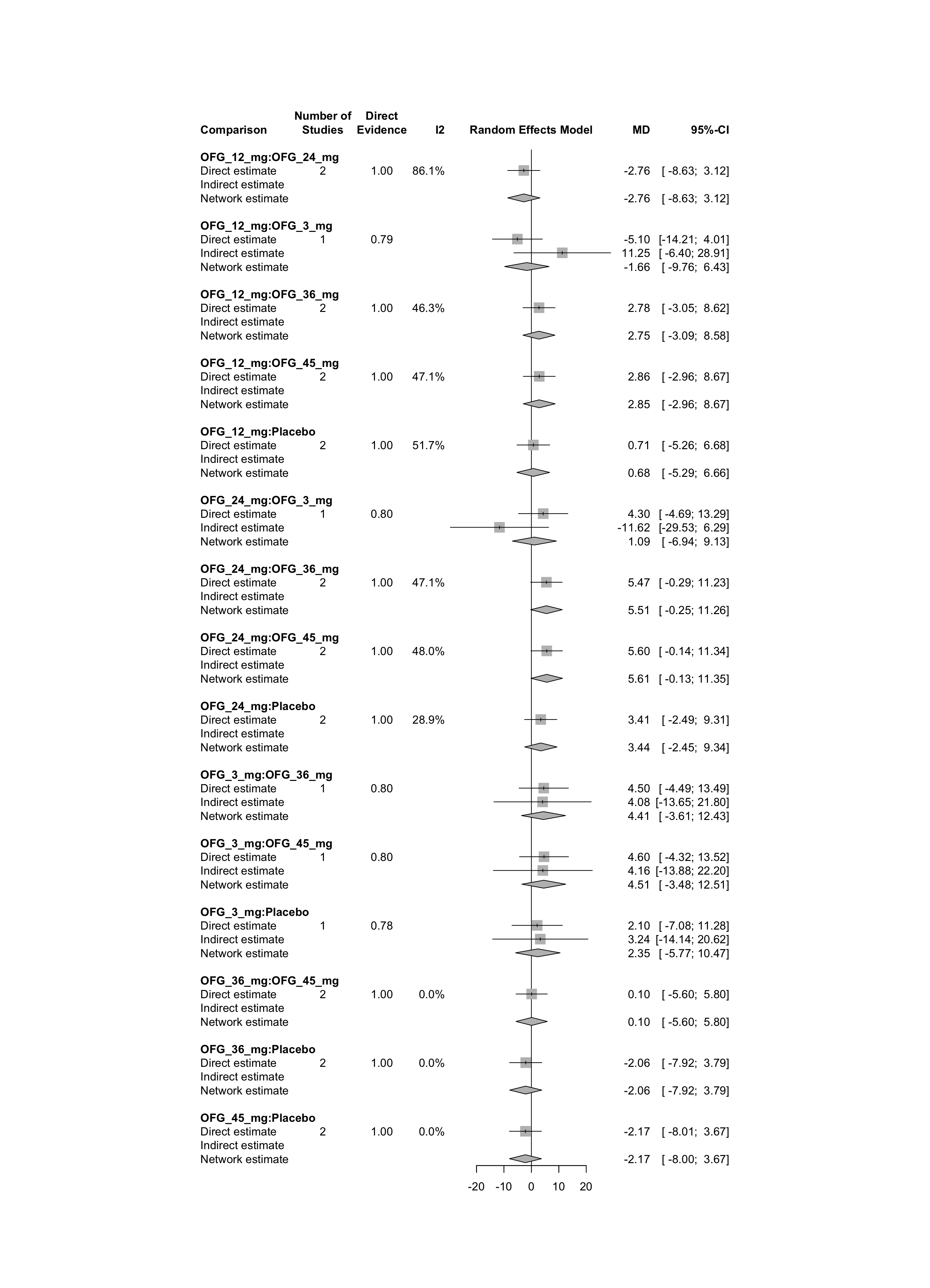

Supplement: Supplementary file 1 — Figure S1: Forest plot for GI AEs leading to discontinuation. Figure S2: Forest plot for nausea. Figure S3: Forest plot for vomiting. Figure S4: Forest plot for diarrhoea. Figure S5: Forest plot for eructation. Figure S6: Forest plot for constipation. Figure S7: Forest plot for abdominal pain. Figure S8: Forest plot for decreased appetite. Figure S9: Forest plot for GERD. Figure S10: Forest plot for dyspepsia. Figure S11: Forest plot for pancreatitis. Figure S12: Forest plot for abdominal distension. Figure S13: Forest plot for hepatic events. Figure S14: Net graphs for safety outcomes: (A) GI AEs leading to discontinuation, (B) nausea, (C) vomiting, (D) diarrhoea, (E) eructation, (F) constipation, (J) abdominal pain, (H) decreased appetite, (I) GERD, (J) dyspepsia, (K) abdominal distension, (L) hepatic events, (M) pancreatitis. Figure S15: Subgroup analysis for GI AEs leading to discontinuation. Figure S16: Subgroup analysis for nausea. Figure S17: Subgroup analysis for vomiting. Figure S18: Subgroup analysis for diarrhoea. Figure S19: Subgroup analysis for dyspepsia. Figure S20: Subgroup analysis for eructation. Figure S21: Subgroup analysis for constipation. Figure S22: Subgroup analysis for decreased appetite. Figure S23: Subgroup analysis for hepatic events. Figure S24: Subgroup analysis for GERD. Figure S25: Subgroup analysis for abdominal pain. Figure S26: Subgroup analysis for pancreatitis. Figure S27: Subgroup analysis for abdominal distension. Figure S28: Subgroup analysis for % change from baseline in ALT at Week 26. Figure S29: Subgroup analysis for % change from baseline in AST at Week 26. Figure S30: Subgroup analysis for % change from baseline in pancreatic amylase at Week 26. Figure S31: Subgroup analysis for % change from baseline in pancreatic lipase at Week 26. Figure S32: Subgroup analysis for % change from baseline in ALP at Week 26. Figure S33: Side‐splitting method for GI AEs leading to discontinuation. Figure S34: Side‐splitting method for na [file EDM2-9-e70222-s001.docx]
